# Oxidative Desymmetrization Enables the Concise Synthesis of a *trans*‐Cyclooctene Linker for Bioorthogonal Bond Cleavage

**DOI:** 10.1002/chem.202203069

**Published:** 2022-11-24

**Authors:** Walter Kuba, Barbara Sohr, Patrick Keppel, Dennis Svatunek, Viktoria Humhal, Berthold Stöger, Marion Goldeck, Jonathan C. T. Carlson, Hannes Mikula

**Affiliations:** ^1^ Institute of Applied Synthetic Chemistry TU Wien Getreidemarkt 9/163 1060 Vienna Austria; ^2^ X-ray Center TU Wien Getreidemarkt 9/164 1060 Vienna Austria; ^3^ Center for Anatomy and Cell Biology Medical University of Vienna 1090 Vienna Austria; ^4^ Center for Systems Biology & Department of Medicine Massachusetts General Hospital Harvard Medical School Boston MA 02114 USA

**Keywords:** bioorthogonal chemistry, cleavable linkers, click chemistry, oxidation, prodrugs

## Abstract

Modified *trans*‐cyclooctenes (TCO) are capable of highly efficient molecular manipulations in biological environments, driven by the bioorthogonal reaction with tetrazines (Tz). The development of click‐cleavable TCO has fueled the field of in vivo chemistry and enabled the design of therapeutic strategies that have already started to enter the clinic. A key element for most of these approaches is the implementation of a cleavable TCO linker. So far, only one member of this class has been developed, a compound that requires a high synthetic effort, mainly to fulfill the multilayered demands on its chemical structure. To tackle this limitation, we developed a dioxolane‐fused cleavable TCO linker (dcTCO) that can be prepared in only five steps by applying an oxidative desymmetrization to achieve diastereoselective introduction of the required functionalities. Based on investigation of the structure, reaction kinetics, stability, and hydrophilicity of dcTCO, we demonstrate its bioorthogonal application in the design of a caged prodrug that can be activated by in‐situ Tz‐triggered cleavage to achieve a remarkable >1000‐fold increase in cytotoxicity.

## Graphical abstract

**Bioorthogonal cleavage** of *trans*‐cyclooctene (TCO) linkers triggered by a click reaction with tetrazines has enabled new concepts in chemical biology and in vivo chemistry. We introduce a new dioxolane‐fused cleavable TCO linker (dcTCO) with high stability and reduced lipophilicity that can be prepared in five steps and demonstrate its application in bioorthogonal prodrug activation, achieving a remarkable >1000‐fold “turn‐on” in cytotoxicity.

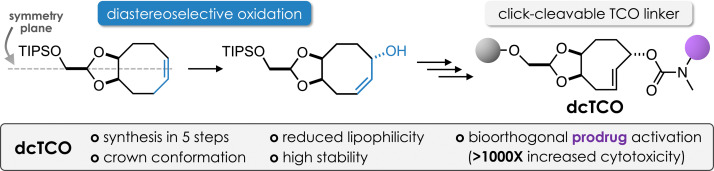

## Introduction

The emergence of biocompatible bond‐cleavage reactions has fueled the design of strategies to spatiotemporally manipulate (bio)molecules and elucidate their functions in complex biological milieus.^[1]^ Thereby, the scope of bioorthogonal chemistry expanded beyond in vivo ligation, enabling methods for the activation of caged proteins^[2]^ and prodrugs,^[3]^ and the controlled cleavage of ligand‐drug^[4]^ and antibody‐fluorophore^[5]^ conjugates.

**Figure 1:**
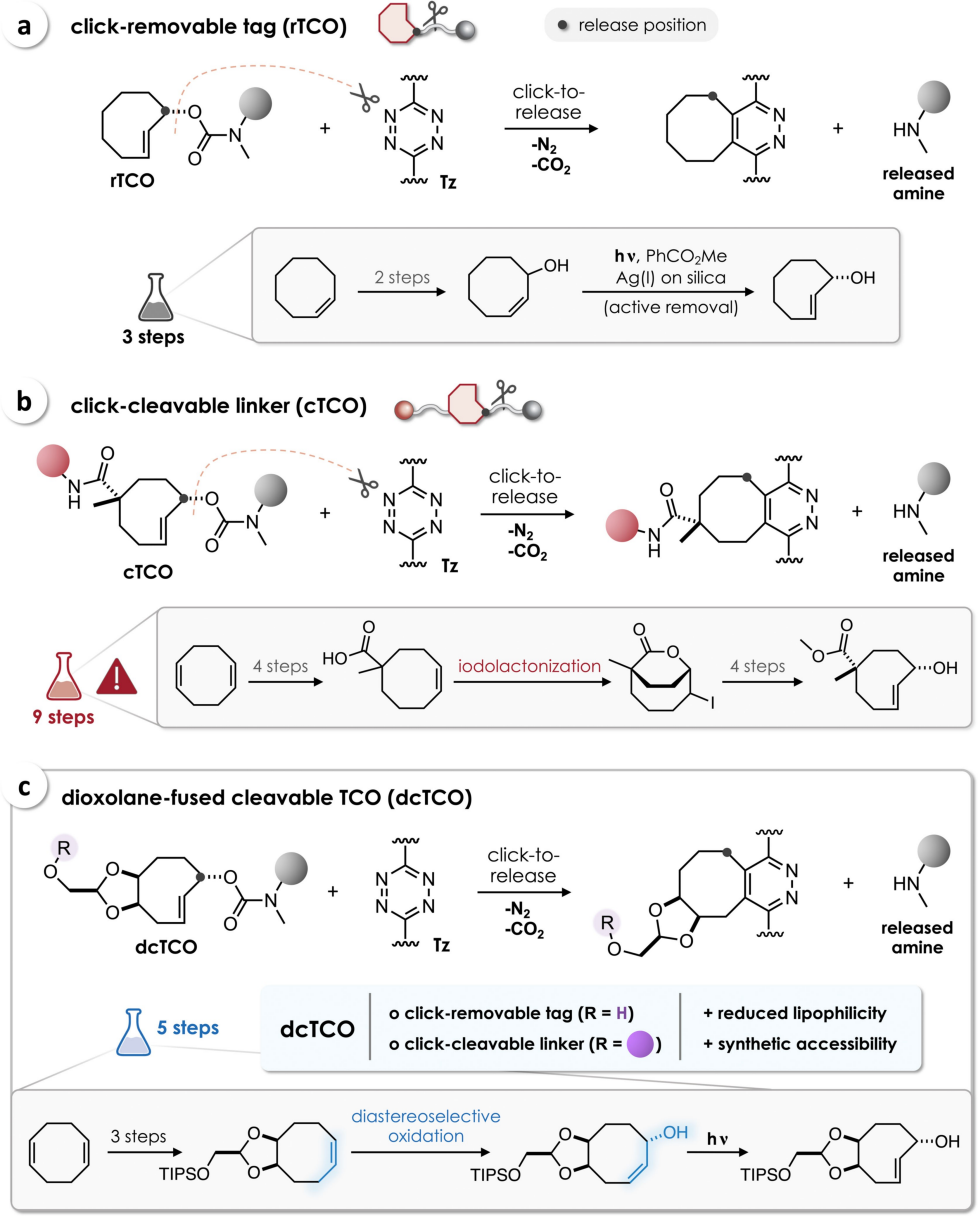
a) Tz‐triggered release of amines with the click‐removable tag rTCO, which can be prepared in 3 synthetic steps. b) cTCO enables bioorthogonal cleavage of conjugates, but is only accessible from a demanding nine‐step procedure that includes a mid‐synthesis iodolactonization. c) The click‐cleavable linker dcTCO can be prepared in five synthetic steps including an oxidative desymmetrization to diastereoselectively introduce the OH group in release position.

The tetrazine (Tz)‐triggered cleavage reaction of *trans*‐cyclooctenes (TCO) stands out from the set of bioorthogonal bond‐cleavage reactions due to its extraordinary click reaction kinetics, tunability, and high versatility.^[6]^ Robillard and co‐workers pioneered the concept of Tz/TCO click‐to‐release (Figure 1a) by introducing a carbamate group in an allylic position of the TCO (release‐TCO, rTCO).^[7]^ Upon bioorthogonal Tz/rTCO ligation through inverse electron demand Diels‐Alder (iEDDA) cycloaddition, subsequent post‐click tautomerization and 1,4‐elimination result in the cleavage of the carbamate, ultimately leading to release of a free amine.^[8]^ As it contains only one functionality for chemical modification, rTCO was previously used as a click‐removable tag, predominantly for the design of caged drugs^[9]^ and proteins.^[2a]^

Aiming for the design of bioorthogonally activatable antibody‐drug conjugates (ADC), Robillard and co‐workers developed a click‐cleavable TCO linker (cTCO, Figure 1b) to enable chemically controlled release of the drug in an in vivo reaction of Tz with cTCO‐linked ADC pretargeted to non‐internalizing cell surface receptors.^[4]^ In contrast, Mejía Oneto and co‐workers injected a Tz‐modified hydrophilic biopolymer into solid tumors to create a bioorthogonal cleavage site for systemically administered TCO‐caged prodrugs.[^9^, ^10^] This approach highlights a second crucial facet of cTCO as it allows pharmacokinetic tuning by attaching an additional hydrophilic moiety, which was used in the design of prodrugs with improved solubility and reduced lipophilicity^[10]^ in comparison to first‐generation rTCO‐caged drugs.^[9]^ This approach is currently being tested in phase I clinical trials,^[11]^ representing the first bioorthogonal chemistry done in humans, and thus narrowing the long‐standing gap between in vivo chemistry as ambitious hope and a useful reality.

The main hurdle for the development and application of click‐cleavable *trans*‐cyclooctene linkers is the lack of efficient synthetic routes given the multi‐layered demands on the chemical structure of the TCO. Firstly, due to the planar chirality of the *trans* double bond, photoisomerization (Figure 1a) generally converts a *cis*‐cyclooctene (CCO) precursor into two TCO diastereomers (except for strained annelated TCOs).^[12]^ For instance, the synthesis of rTCO yields two isomers with the allylic OH in axial or equatorial position.^[7]^ It is thus crucial to design a diastereoselective synthetic strategy to install all functionalities in a fixed stereochemical relationship. Furthermore, to allow stepwise modification of the TCO linker, the allylic OH (release position) and the functional group required for additional conjugation must be chemically orthogonal or distinguishable from each other (e. g., by using different functional groups or introducing a protecting group at an earlier stage of the sequence). Based on the first synthesis developed by Robillard et al.,^[4a]^ an optimized procedure has recently been reported to obtain cTCO in 9 steps starting from 1,4‐cyclooctadiene (Figure 1b).^[13]^ An iodolactonization represents the key step to achieve the *cis* configuration of the CO_2_Me moiety and the allylic OH. The methyl group in α‐position to the ester prevents undesired epimerization during further steps of the synthesis and subsequent modification of cTCO.^[4a]^ Although it provides access to the requisite single CCO diastereomer (as a racemic mixture), the preparation of cTCO demands a substantially higher effort (9 steps)^[13]^ than the synthesis of rTCO (3 steps, Figure 1a).^[7]^ To tackle this limitation, we aimed to directly introduce an OH group in release position by diastereoselective oxidation of functionalized cyclooctenes.

Here we report the development of a dioxolane‐fused cleavable TCO (dcTCO) that can be prepared in only five steps, including stereocontrolled introduction of an allylic OH by oxidative desymmetrization of a known TCO precursor (Figure 1c). Despite the *cis*‐fused dioxolane we show that dcTCO exists in crown conformation, enabling superb stability while also achieving efficient cleavage upon reaction with Tz. Bioorthogonal application of dcTCO as a click‐cleavable linker is demonstrated by the design of a prodrug with >1000‐fold reduced cytotoxicity that was fully restored upon in‐situ click‐to‐release.

## Results and Discussion

**Figure 2:**
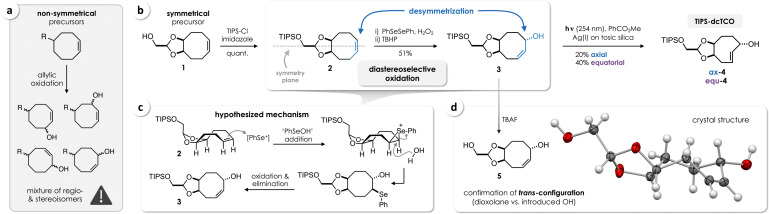
a) Allylic oxidation of nonsymmetrical cyclooctenes resulted in the formation of isomeric mixtures. b) Oxidative desymmetrization of the TIPS‐protected dioxolane‐fused cyclooctene **2** enabled stereocontrolled introduction of an allylic OH group to obtain TCO precursor **3**, which upon photoisomerization provided access to TIPS‐dcTCO (**4**) in five steps from 1,4‐cyclooctadiene. c) Hypothesized mechanism of the diastereoselective oxidation of **2** (shown for one enantiomer). d) The crystal structure of TIPS‐deprotected (racemic) **5** confirmed the *trans*‐configuration of the allylic OH relative to the fused dioxolane.

Initial attempts to install an OH group in release position by allylic oxidation^[14]^ of nonsymmetrical substituted cyclooctenes resulted in the formation of complex mixtures of regio‐ and stereoisomers (Figure 2a). Introduction of sterically demanding groups to control the stereochemistry of the oxidation, for instance by triisopropylsilyl (TIPS) protection of 1‐hydroxycyclooct‐4‐ene,^[12a]^ had no effect on the outcome of the reaction. This observation prompted us to use a symmetric precursor to effectively restrict the number of possible products. Therefore, we turned to the known TCO precursor **1** (Figure 2b), a key intermediate in the synthesis of a strained dioxolane‐fused TCO (d‐TCO) that has previously been designed for accelerated Tz/TCO ligation.^[12c]^ TIPS‐protection of **1** (which is accessible in 2 steps from 1,4‐cyclooctadiene) afforded the symmetric cyclooctene derivative **2**. In the following key step, we aimed for oxidation of **2** mediated by diphenyl diselenide and phenylselenic acid formed in situ, to introduce an allylic OH group.^[14a]^ Notably, we observed diastereoselective formation of **3**, with the OH in *trans*‐position relative to the fused dioxolane (Figure 2b). The outcome of this oxidative desymmetrization is in line with the hypothesized mechanism encompassing 1) the electrophilic addition of PhSe^+^ (i. e., “PhSeOH” or other selenium(II) electrophiles) forging the *cis* adduct given the boat‐like conformation of **2** (Figure 2c, supported by conformational analysis, see the Supporting Information, Figure S4), 2) subsequent ring‐opening through a backside attack of water resulting in a β‐hydroxyphenyl selenide adduct, and 3) oxidation with *tert*‐butyl hydroperoxide (TBHP) followed by thermal elimination to yield **3** (Figure 2c). Furthermore, this mechanism accounts for the altered position of the double bond in relation to the fused dioxolane, which in addition to the relative configuration of **3** could be confirmed by single crystal X‐ray diffraction upon removal of the TIPS protecting group (Figure 2d).

We tested both AgNO_3_ impregnated silica[^12a^, ^12b^, ^12c^] and Ag^I^ immobilization on sulfonated silica gel (tosic silica)^[12d]^ for active removal of TCO during the photoisomerization process. The two TIPS‐dcTCO isomers **ax‐4** and **equ‐4** (Figure 2b) were obtained in a substantially higher yield with Ag(I)/tosic silica (60 %) than with AgNO_3_/silica (10 %). While an additional synthetic step has previously been used after photo‐isomerization to obtain both stereoisomers of cTCO,^[13]^ **ax‐4** and **equ‐4** could readily be separated by column chromatography.

**Figure 3:**
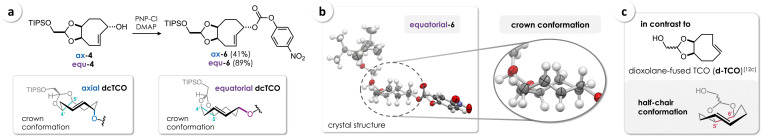
a) Synthesis of the *p*‐nitrophenyl (PNP) carbonates **ax‐6** and **equ‐6** to activate the release position for further modification. Both axial and equatorial dcTCO (dioxolane‐fused in 4’/5’‐position relative to the *trans* double bond) adopt crown conformation. b) Crystal structure of **equ‐6** (one enantiomer shown). c) In contrast, the 5’/6’‐dioxolane‐fused click‐tag d‐TCO is locked in a more strained half‐chair conformation.

To activate the allylic OH for further modification of the release position, the *p*‐nitrophenyl (PNP) carbonates **ax‐6** and **equ‐6** were prepared (Figure 3a). We were able to crystallize and determine the crystal structure of **equ‐6** by single crystal X‐ray diffraction, revealing the crown conformation of dcTCO (Figure 3b). This finding is in agreement with the results of computational investigations (conformational analyses) suggesting that dcTCO exists in aqueous solution predominantly (>95 %) in crown conformation (see the Supporting Information, Table S3). In contrast, the previously developed click‐tag d‐TCO is locked in a half‐chair conformation (Figure 3c).^[12c]^ Hence, shifting the fused dioxolane from 5’/6’ (d‐TCO) to the 4’/5’ position (dcTCO) relative to the double bond allows the TCO to adopt the less strained crown conformation (Figure 3a), suggesting similar iEDDA reactivity of dcTCO compared to rTCO, which is known to exist in crown conformation,^[7]^ and high stability of dcTCO in aqueous solution.

**Figure 4:**
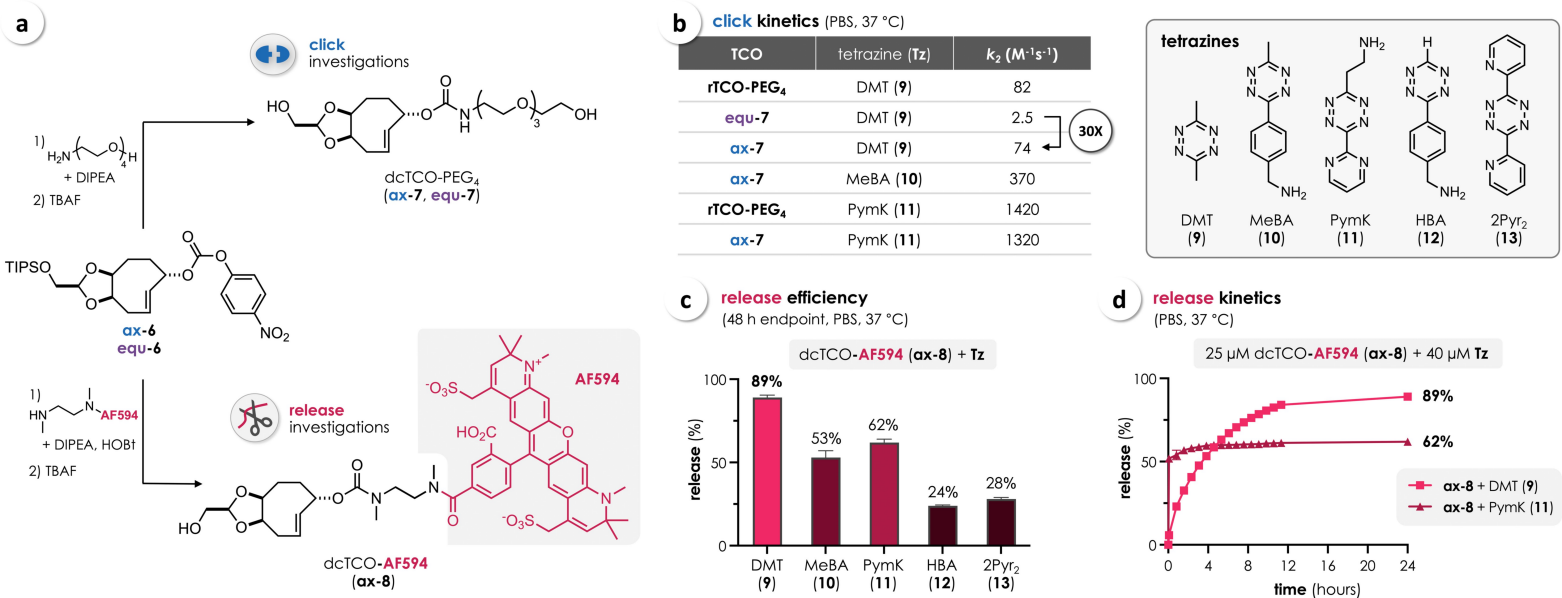
a) Synthesis of the water‐soluble dcTCO‐PEG_4_ isomers **ax‐7** and **equ‐7** for kinetic investigations, and dcTCO‐AF594 (**ax‐8**) as a fluorescently labeled release probe. b) Second‐order rate constants (*k*_2_) for the reactions of **ax‐7**, **equ‐7**, and rTCO‐PEG_4_ with tetrazines **9–13** in PBS at 37 °C (see Table S1 for the results of all performed measurements). c) Tz‐triggered release of **ax‐8** in PBS at 37 °C as determined by HPLC after a reaction time of 48 h (endpoint, mean±SD, *n*=3). d) Release kinetics of the reactions of **ax‐8** (25 μM) with DMT (**9**) and PymK (**11**) in PBS at 37 °C (serial HPLC measurements, mean±SD, *n*=3).

To test these assumptions, we prepared the water‐soluble dcTCO‐PEG_4_ isomers **ax‐7** and **equ‐7** (Figure 4a). Second‐order rate constants (*k*_2_) of click reactions with selected Tz (**9**–**13**) were determined by stopped‐flow spectrophotometry in PBS at 37 °C (Figure 4b, Table S1). As expected, we observed nearly identical iEDDA reactivities for **ax‐7** and rTCO‐PEG_4_ ^[8]^ (axial isomer), with *k*_2_ of approximately 80 and 1400 M^−1^ s^−1^ for the reactions with 3,6‐dimethyl‐1,2,4,5‐tetrazine (DMT, **9**) and the ammonium‐functionalized pyrimidyl‐substituted tetrazine PymK (**11**),^[15]^ respectively (Figure 4b). As previously described for the two stereoisomers of rTCO,^[7]^ the equatorial dcTCO derivative **equ‐7** was found to be considerably less reactive (33‐fold) than **ax‐7**, due to increased steric demand of the carbamate in the transition state when being in equatorial release position.

To investigate the stability of dcTCO in buffered aqueous solution, **ax‐7** was incubated in PBS at 37 °C and the TCO‐concentration was determined over the course of several days by click titration with bis(2‐pyridyl)‐Tz (2Pyr_2_, **13**), revealing 99 % intact dcTCO after 96 h, thus even slightly exceeding the high stability of rTCO (93 %, Figure S2).

Given the higher iEDDA reactivity of axial dcTCO we focused on this isomer to investigate Tz‐triggered bioorthogonal release. Therefore, **ax‐6** was conjugated with AlexaFluor 594 (AF594) via an *N*,*N’*‐dimethylethylenediamine (DMEDA) linker to afford the fluorescently labeled dcTCO probe **ax‐8** (Figure 4a). An *N*‐methyl group was incorporated into the carbamate at the release position to prevent potential post‐click intramolecular cyclization, which we have previously identified as an undesired pathway diminishing overall release when using bis‐alkyl‐Tz such as DMT (**9**).^[8]^ For initial assessment of the release efficiency, **ax‐8** was reacted with selected Tz (**9**–**13**) in PBS at 37 °C and the reaction mixtures were analyzed after 48 h by HPLC (Figure 4c). We observed a dcTCO‐release of 89 % with DMT (**9**), 53 % with the alkyl‐aryl‐Tz MeBA (**10**), and 62 % with PymK (**11**), similar to previous findings^[15]^ and matching very recently reported data for the release of rTCO‐DMEDA‐conjugates.^[16]^ As indicated by LCMS data, and in line with previous reports,[^5a^, ^8^, ^16^] the non‐released by‐products were assigned to an oxidized pyridazine dead‐end, formed upon click with DMT (**9**), and stable dihydropyridazine tautomers with the aryl‐substituent of MeBA (**10**) and PymK (**11**), respectively, in a head‐to‐head position[^5a^, ^8^] relative to the dcTCO‐carbamate. In contrast, the reaction of **ax‐8** with the monosubstituted aryl‐Tz HBA (**12**) and 2Pyr_2_ (**13**) resulted in only <30 % release (Figure 4c), consistent with reported data for rTCO and other cleavable TCOs.[^5a^, ^7^, ^13^, ^15^] Release kinetics of the reactions of **ax‐8** with DMT (**9**) and PymK (**11**) were studied by serial HPLC measurements. Considering the concentrations of both reactants (25 μM **ax‐8**, 40 μM Tz) and the respective *k_2_* values (Figure 4b), complete click (>99 %) with DMT (**9**) and PymK (**11**) is achieved in <1 h and <3 min, respectively. The click‐triggered elimination of AF594‐DMEDA upon reaction with DMT (**9**) displayed exponential characteristics reaching >80 % release after 10 h and 89 % after 24 h (Figure 4d). In contrast, bond cleavage upon reaction with PymK (**11**) is notably biphasic (due to the known directing effect of the ammonium substituent on post‐click tautomerization^[15]^), showing instantaneous elimination of around 50 % (too fast to be resolved by HPLC) and a slow second phase, contributing less to the overall release of 62 % after 24 h (Figure 4d).

To estimate the lipophilicity of dcTCO relative to rTCO and cTCO, log *P* values at pH 7.4 were calculated (c log *P*_7.4_, Chemicalize) for a range of TCO derivatives and conjugates of hydrophobic and hydrophilic compounds including selected cytotoxic drugs (see the Supporting Information). The obtained results indicate an approx. 55‐fold reduced lipophilicity of dcTCO as a click‐removable tag in comparison to rTCO and cTCO, with an average difference in c log *P*_7.4_ values of −1.74 and −1.67, respectively. As a click‐cleavable linker dcTCO is still estimated to be significantly less lipophilic (ca. fourfold) compared to cTCO, as indicated by an average Δc log *P*_7.4_ of −0.57 (Figure S3).

**Figure 5:**
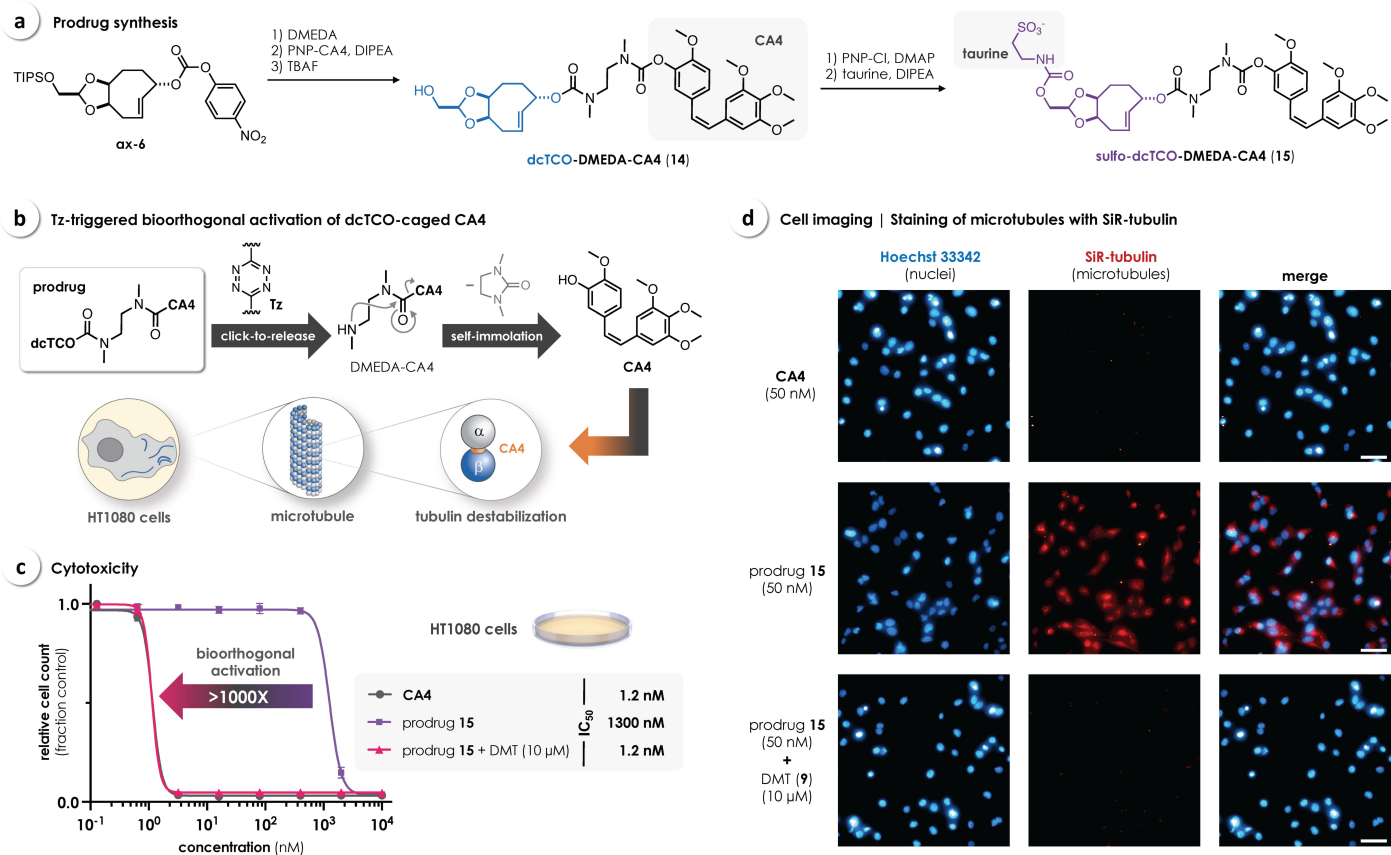
a) Synthesis of the dcTCO‐caged prodrug **15** by incorporation of *N*,*N*’‐dimethylethylenediamine (DMEDA) as a self‐immolative linker. b) Mechanism of the Tz‐triggered release of **CA4** and its binding to microtubules leading to cell death. c) A concentration of prodrug **15** >1000 times higher than for **CA4** was needed to reduce the cell count (mean±SD, *n*=3) of HT1080 fibrosarcoma cancer cells by 50 % (IC_50_) over the course of 72 h of treatment. Drug cytotoxicity was fully restored upon an in‐situ click‐to‐release reaction of **15** with DMT (**9**). d) Fluorescence microscopy of HT1080 cells stained with Hoechst 33342 and SiR‐tubulin^[18]^ after 6 h of treatment with i) **CA4**, ii) **15** alone, and iii) **15**+**DMT** confirmed the antimitotic effect of released **CA4** at the cellular level; scale bars: 50 μm.

To demonstrate bioorthogonal cleavage of a dcTCO linker in a biological environment, we designed a prodrug of the antimitotic agent combretastatin A‐4 (**CA4**).^[17]^ Analogous to our recently reported approach for the Tz‐triggered release of phenols,^[16]^ we conjugated dcTCO to **CA4** through DMEDA as a self‐immolative linker. dcTCO‐DMEDA‐CA4 (**14**) was obtained by reacting **ax‐6** with DMEDA and PNP‐CA4, followed by removal of the TIPS protecting group (for details see the Supporting Information). The additional OH‐functionality of **14** allowed further modification by carbamate coupling with 2‐aminoethanesulfonic acid (taurine) to obtain the water‐soluble sulfo‐dcTCO‐caged prodrug **15** (Figure 5a). Mechanistically, Tz/dcTCO click‐to‐release of **15** leads to formation of a labile DMEDA‐CA4 intermediate that, upon self‐immolation, spontaneously liberates the active drug. Binding of released **CA4** to β‐tubulin (at the interface with α‐subunits, i. e., colchicine binding site) induces depolymerization of microtubules leading to cell death (Figure 5b).^[17]^

Prior to cell experiments, we investigated the bioorthogonal release of **CA4** in solution. Therefore, we monitored the reaction of **14** with DMT (**9**) by serial HPLC measurements. In comparison to rTCO‐DMEDA‐CA4 (**16**), we observed a moderately faster elimination, leading to 85 % release of **CA4** within 4 h (Figure S1). We then investigated prodrug **15** in HT1080 human fibrosarcoma cancer cells, revealing a remarkable >1000‐fold reduced cytotoxicity compared to the parent drug. While we have recently reported a 750‐fold reduced cytotoxicity of an analogous cTCO‐caged **CA4** prodrug, the observed “turn‐on” in toxicity upon reaction with DMT (**9**) was limited to a factor of 220.^[16]^ In contrast, cytotoxicity could be fully restored via bioorthogonal activation of dcTCO‐prodrug **15** by in‐situ reaction with DMT (**9**), thus providing a therapeutic window of three orders of magnitude (>1000‐fold, Figure 5c). Moreover, this number notably exceeds the reported data for cTCO‐caged SQP33 (a doxorubicin‐prodrug with 83‐fold reduced cytotoxicity), currently being evaluated in a phase I study.^[11]^

To investigate drug action at the cellular level, differentially treated HT1080 cells were stained with SiR‐tubulin, a fluorescent probe that selectively binds to intact microtubules.^[18]^ Cell imaging by fluorescence microscopy showed depletion of the signal upon in‐situ reaction of prodrug **15** and DMT (**9**) comparable to the parent drug (Figure 5d), confirming the antimitotic effect of released **CA4**. In contrast, no significant effect on microtubule staining was observed after treatment with the prodrug only (see also Figure S4 for further control experiments).

## Conclusion

Motivated by the limited synthetic access to cleavable *trans*‐cyclooctene linkers, we developed a concise procedure for the diastereoselective preparation of dcTCO, applying an oxidative desymmetrization in the key step. Despite the *cis*‐fused dioxolane, dcTCO adopts the less‐strained crown conformation, as confirmed by computational studies and single‐crystal X‐ray diffraction. Furthermore, this structural insight is in agreement with the observed iEDDA reactivity and release efficiency of dcTCO as well as its high stability (99 % after 96 h in PBS at 37 °C), which is essential for the design of effective prodrug strategies. In addition, log *P* calculations indicate a significantly reduced lipophilicity of dcTCO in comparison to that of the known click‐removable tag rTCO and the click‐cleavable linker cTCO. Exploiting these findings, we designed the dcTCO‐caged CA4 prodrug **15**, making use of the additional OH functionality of dcTCO to introduce a sulfonate moiety and thereby further reduce lipophilicity. Cell experiments revealed a >1000‐fold reduced cytotoxicity of **15** compared to **CA4** that was fully restored upon Tz‐triggered bioorthogonal cleavage of the dcTCO linker.

Deposition Numbers 2108332 (for **5**), and 2108333 (for **equ‐6**) contain the supplementary crystallographic data for this paper. These data are provided free of charge by the joint Cambridge Crystallographic Data Centre and Fachinformationszentrum Karlsruhe Access Structures service.

## Conflict of interest

The authors declare no conflict of interest.

1

## Supporting information

As a service to our authors and readers, this journal provides supporting information supplied by the authors. Such materials are peer reviewed and may be re‐organized for online delivery, but are not copy‐edited or typeset. Technical support issues arising from supporting information (other than missing files) should be addressed to the authors.

Supporting Information

## Data Availability

The data that support the findings of this study are available from the corresponding author upon reasonable request.
