# Supplementary material for: Oxidative Desymmetrization Enables the Concise Synthesis of a trans‐Cyclooctene Linker for Bioorthogonal Bond Cleavage
Source: Chemistry. 2022 Nov 24;29(3):e202203069. doi: 10.1002/chem.202203069 (PMC10098836; doi:10.1002/chem.202203069)
Supplement: Supplementary file 1 — Supporting Information [file CHEM-29-0-s001.pdf]

Chemistry–A European Journal

Supporting Information

Oxidative Desymmetrization Enables the Concise Synthesis of a *trans*-Cyclooctene Linker for Bioorthogonal Bond Cleavage

Walter Kuba, Barbara Sohr, Patrick Keppel, Dennis Svatunek, Viktoria Humhal, Berthold Stöger, Marion Goldeck, Jonathan C. T. Carlson, and Hannes Mikula*

Table of Contents

1)	Synthesis	S2
2)	Click kinetics	S10
3)	Release experiments	S11
4)	Stability measurements	S12
5)	Log*P* calculations	S13
6)	Cell experiments	S14
7)	Single crystal X-ray diffraction	S15
8)	Computational studies	S16
9)	NMR spectra and chromatograms	S19
10)	References	S39

1) Synthesis

General methods

Unless otherwise noted, reactions were carried out under an atmosphere of argon in air-dried glassware with magnetic stirring. Air- and/or moisture-sensitive liquids were transferred via syringe. All reagents were purchased from commercial sources without further purification. Combretastatin A-4 (**CA4**) was obtained from BLD Pharmatech. MBA (**10**) and HBA (**12**) were purchased from Click Chemistry Tools (AZ, USA). $2Pyr_2$ (**13**) was obtained from Sigma Aldrich. Alexa Fluor 594 (AF594)-NHS ester was purchased from Fluoroprobes (AZ, USA). Solvents used for column chromatography were purchased from Donau Chemie AG. DCM, THF and diethyl ether were dried using PURESOLV-columns (Inert Corporation). Dry DMF was obtained from ACROS Organics and stored under argon.

Thin layer chromatography was performed using TLC plates on aluminum support (Merck, silica gel 60, fluorescent indicator 254). Column chromatography was performed using a BUCHI Sepacore Flash System (2 x BUCHI Pump Module C-605, BUCHI Pump Manager C-615, BUCHI UV Photometer C-635, and BUCHI Fraction Collector C-660) and a Reveleris X2 Purification System (BUCHI). Silica gel 60 (40-63 µm) was obtained from Merck. Kinetex AXIA LC columns (C18 or C8; 5 µm, 100 Å, 100 x 30.0 mm, Phenomenex) were used for preparative HPLC. HPLC grade solvents were purchased from VWR (USA).

^{1}H and ^{13}C NMR spectra were recorded on a Bruker AC 200 MHz, Bruker Avance UltraShield 400 MHz or Bruker Ascend 600 MHz spectrometer at 20 °C. Chemical shifts are reported in ppm (δ) relative to tetramethylsilane and calibrated using solvent residual peaks. Data is shown as follows: Chemical shift, multiplicity (s = singlet, d = doublet, t = triplet, q = quartet, quint = quintet, m = multiplet, b = broad signal), coupling constants (*J*, Hz) and integration.

GC-MS analysis was done on a Thermo Finnigan GC 8000 Top gas chromatograph using a BGB5 column (l=30 m, di=0,32 mm, 1 µm coating thickness) coupled to a Voyager Quadrupol mass spectrometer (electron ionization, 70 eV).

HPLC-MS (LCMS) analysis was performed on a Nexera X2 system (Shimadzu) comprised of LC-30AD pumps, a SIL-30AC autosampler, a CTO-20AC column oven, and a DGU-$20A_{5/3}$ degasser module. Detection was done using an SPD-M20A photo diode array, an RF-20Axs fluorescence detector, an ELS-2041 evaporative light scattering detector (JASCO) and an LCMS-2020 mass spectrometer (ESI/APCI). If not stated otherwise, all separations were performed using a Waters XSelect CSH™ C18 2.5 µm (3.0 x 50 mm) column XP at 40 °C and a flowrate of 1.7 mL/min with 0.1 % aqueous formic acid or ammonium formate buffer (2.5 mM, pH 8.4) and acetonitrile (gradient elution). Acidic HPLC conditions (acetonitrile/0.1 % formic acid) 0 min: 5 %, 0.15 min: 5 %, 2.20 min: 98 %, 2.50 min: 98 %; Buffered HPLC conditions (acetonitrile/2.5 mM ammonium formate buffer, pH 8.4) 0 min: 5 %, 0.15 min: 5 %, 2.20 min: 98 %, 2.50 min: 98 %. See section 3 for further details on buffer preparation.

HRMS analysis of aqueous or acetonitrile solutions of the compounds (sample concentration: 10 ppm) was carried out on an Agilent 6545 Q-TOF mass spectrometer equipped with an Agilent Dual AJS ESI source. The mass spectrometer was connected to a liquid chromatography system comprised of an Agilent G7167B multi sampler, an Agilent G7120A binary pump with degasser and an Agilent G7116B oven (Agilent Technologies, Palo Alto, CA, USA). A SecurityGuard Cartridge (Phenomenex) was used as a stationary phase. Data evaluation was performed using Agilent MassHunter Workstation Qualitative Analysis 10.0. Identification was based on peaks obtained from extracted ion chromatograms (extraction width ± 20 ppm).

Synthesis of TIPS-dcTCO-PNP (ax-6, equ-6)

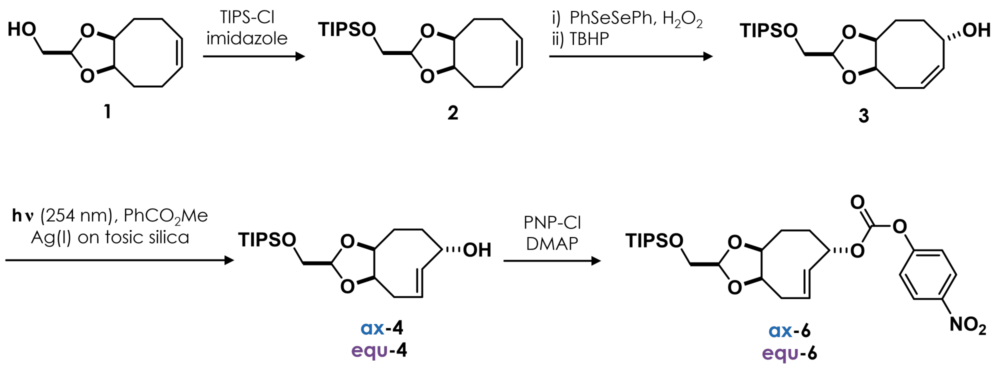

TIPS-dCCO (2)

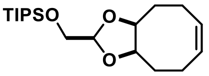

To a solution of dioxolane-fused *cis*-cyclooctene **1**[1] (*syn*-diastereomer, 0.55 g, 3 mmol) in anhydrous DCM (20 mL) was added imidazole (0.49 g, 7.2 mmol) followed by triisopropylsilyl chloride (TIPS-Cl, 0.69 g, 3.6 mmol). The resulting reaction mixture was stirred for 24 h at rt before the precipitate (imidazolium chloride) was removed by filtration. The filtrate was washed with a saturated aqueous NH_4Cl, dried with Na_2SO_4, and concentrated. Column chromatography (90 g SiO_2, hexanes/EtOAc gradient elution, 1-5% EtOAc) afforded **2** as a colorless oil (1.02 g, quant.). At a larger scale (~15X), starting from 8 g (43.4 mmol) of **1**, 11.3 g (76%) of **2** were obtained after repeated column chromatography. **^{1}H NMR** (400 MHz, $CDCl_3$) δ 5.67 – 5.51 (m, 1H), 4.91 (t, *J* = 4.1 Hz, 1H), 4.21 – 4.09 (m, 2H), 3.74 (d, *J* = 4.1 Hz, 2H), 2.55 – 2.43 (m, 2H), 2.14 – 2.00 (m, 4H), 1.99 – 1.90 (m, 2H), 1.17 – 1.02 (m, 21H); **^{13}C NMR** (101 MHz, $CDCl_3$) δ 129.3, 102.6, 79.1, 66.1, 28.6, 23.6, 18.0, 12.1; **HRMS** $[M+H]^+$ calcd. 341.2507 for $C_{19}H_{37}O_3Si^+$, found 341.2506.

TIPS-dcCCO (3)

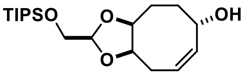

Similar to a procedure described by Hori *et al.*,[2] diphenyl diselenide (0.55 g, 1.76 mmol) was dissolved in anhydrous DCM (6 mL) and cooled to 0 °C. To the orange-colored solution was added dropwise H_2O_2 (0.17 g of a 35% solution, 1.76 mmol) and the reaction mixture was stirred vigorously for 30 min (precipitation of white crystals). After addition of Na_2SO_4 (0.35 g, 2.5 mmol) the reaction mixture was stirred for 30 min before the ice bath was removed and **2** (0.4 g, 1.17 mmol) was added. The mixture was stirred rapidly for 24 h at rt, cooled to 0 °C and after addition of *tert*-butyl hydroperoxide (1.2 mL of a 5.5 M solution in decane, 6.6 mmol) stirred at rt for another 24 h. The white precipitate was removed by filtration and the filtrate was diluted with EtOAc (20 mL) before washing with 5% aqueous Na_2CO_3 (10 mL), water (10 mL), 10% aqueous $FeSO_4$ (10 mL), water (10 mL), saturated aqueous $NaHCO_3$ (10 mL), water (10 mL) and saturated aqueous NaCl (10 mL). The organic layer was dried over Na_2SO_4, filtered and concentrated. The orange-colored residue was purified by column chromatography (SiO_2, hexanes/EtOAc, 1-25% EtOAc gradient elution) to obtain **3** as a pale-yellow solid (0.215 g, 51%). At a larger scale (10X), starting from 4 g (11.7 mmol) of **2**, 1.35 g (32%) of **3** were obtained following the same procedure. **^{1}H NMR** (400 MHz, $CDCl_3$) δ 5.63 (dd, *J* = 10.9, 5.1 Hz, 1H), 5.62 – 5.50 (m, 1H), 4.93 (t, *J* = 4.1 Hz, 1H), 4.57 – 4.47 (m, 1H), 4.05 (dd, *J* = 11.1, 5.5 Hz, 1H), 4.01 – 3.92 (m, 1H), 3.69 (dd, *J* = 4.2, 2.4 Hz, 2H), 2.47 – 2.33 (m, 2H), 1.97 – 1.86 (m, 2H), 1.72 – 1.52 (m, 2H), 1.10 – 1.02 (m, 21H); **^{13}C NMR** (101 MHz, $CDCl_3$) δ 139.1, 123.4, 103.8, 80.1, 79.9, 71.0, 66.1, 36.7, 29.9, 27.9, 18.0, 12.0; **HRMS** $[M+Na]^+$ calcd. 379.2275 for $C_{19}H_{36}NaO_4Si^+$, found 379.2265.

TIPS-dcTCO (ax-4, equ-4)

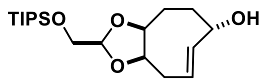

A mixture of Et_2O and n-heptane (v/v = 1:1, 700 mL) was degassed with argon and sonicated for 15 minutes before it was filled in a continuous flow photochemistry apparatus consisting of a 1 L three-necked, round-bottomed flask with a reflux condenser, metering pump, flow photoreactor (quartz glass tube with one 55 W low-pressure mercury lamps emitting a dominant wavelength of 254 nm) and a cartridge.[3] The cartridge was filled with 8.9 g Ag(I)-tosic silica gel between two small layers of silica gel.[4] Precursor **3** (1.2 g, 3.4 mmol) and methyl benzoate (0.92 g, 6.7 mmol) were dissolved in Et_2O/n-heptane (v/v = 1:1, 15mL) and then added to the system. The flowrate was set to 100 mL/min. To equilibrate the system the solution was circulated for 20 min before the UV lamp was turned on. After irradiation for 20 h the cartridge was flushed with MTBE (200 mL) before saturated methanolic ammonia (150 mL) was passed through the column to elute the *trans*-cyclooctene product. The solution was concentrated and the residue was purified by column chromatography (90 g SiO_2, hexanes/EtOAc, 0-60% EtOAc gradient elution) to afford **ax-4** (241 mg, 20%) and **equ-4** (477 mg, 40%).

Note: The yield of **ax-4/equ-4** substantially increased upon recycling and reusing the Ag(I)-tosic silica gel.

ax-4: **^{1}H NMR** (400 MHz, CD_2Cl_2) δ 6.11 – 6.00 (m, 1H, a), 5.94 (dd, J = 16.8, 2.6 Hz, 1H, b), 4.82 (t, J = 3.9 Hz, 1H, i), 4.53 (s, 1H, c), 4.18 (td, J = 6.1, 3.8 Hz, 1H, g), 4.08 – 4.02 (m, 1H, f), 3.83 (dd, J = 3.9, 0.6 Hz, 2H, j), 2.62 – 2.49 (m, 2H, h,h’), 2.09 – 1.97 (m, 1H, d), 1.86 – 1.77 (m, 2H, e,e’), 1.71 – 1.63 (m, 1H, d’), 1.48 (s, 1H, m), 1.20 – 1.09 (m, 3H, k), 1.11 – 0.99 (m, 18H, l); **^{13}C NMR** (101 MHz, CD_2Cl_2) δ 138.1 (=CH, B), 125.2 (=CH, A), 102.9 (OCO, I), 84.4 (O-C̲H, G), 77.9 (O-C̲H, H), 70.2 (C̲HOH, C), 65.6 (OC̲H$_2$, J), 34.1 (C̲H$_2$, D), 32.5 (C̲H$_2$, H), 24.8 (C̲H$_2$, E), 18.2 (C̲H$_3$, L), 12.5 (C̲H, K); **HRMS** $[M+Na]^+$ calcd. 379.2275 for $C_{19}H_{36}NaO_4Si^+$, found 379.2283.

equ-4: ^{1}H NMR (400 MHz, CD_2Cl_2) δ 5.95 (ddd, J = 16.1, 9.6, 6.0 Hz, 1H, a), 5.42 (ddd, J = 16.8, 9.3, 1.3 Hz, 1H, b), 4.84 (t, J = 4.2 Hz, 1H, i), 4.36 (dt, J = 7.4, 4.3 Hz, 1H, g), 4.16 (td, J = 9.5, 5.5 Hz, 1H), 3.78 (ddd, J = 10.4, 4.5, 2.2 Hz, 1H, f), 3.68 (d, J = 4.2 Hz, 2H, j), 2.68 (dddd, J = 14.5, 8.9, 7.3, 1.2 Hz, 1H, h), 2.48 (ddd, J = 14.7, 6.0, 4.2 Hz, 1H, h‘), 2.14 (ddd, J = 13.4, 7.6, 5.4 Hz, 1H, d), 1.87 (s, 1H, m), 1.69 (ddd, J = 14.7, 7.6, 2.2 Hz, 1H e), 1.53 – 1.45 (m, 1H, e‘), 1.44 – 1.31 (m, 1H, d‘), 1.16 – 1.10 (m, 3H, k), 1.10 – 1.04 (m, 18H, l); **^{13}C NMR** (101 MHz, CD_2Cl_2) δ 138.7 (=CH, B), 128.2 (=CH, A), 103.1 (OCO, I), 85.7 (O-C̲H, G), 82.4 (O-C̲H, F), 75.6 (C̲HOH, C), 66.6 (OC̲H$_2$, J), 37.6 (C̲H$_2$, D), 33.2 (C̲H$_2$, E), 32.9 (C̲H$_2$, H), 18.2 (C̲H$_3$, L), 12.5 (C̲H$_2$, K); **HRMS** $[M+Na]^+$ calcd. 379.2275 for $C_{19}H_{36}NaO_4Si^+$, found 379.2284.

Assignment of signals according to 2D NMR (see *9. NMR spectra and chromatograms*)

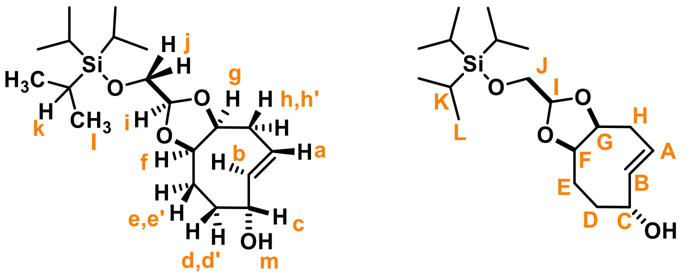

dcCCO (5)

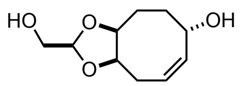

To a solution of **3** (400 mg, 1.12 mmol) in anhydrous THF (20 mL) was added TBAF (1.0 M in THF, 1.46 mL, 1.5 mmol) and the reaction mixture was stirred for 1 h at room temperature. The solvent was evaporated and the residue was purified by column chromatography (SiO_2, DCM/EtOAc gradient elution) to afford **5** as a white solid (193 mg, 86%). Single crystals of **5** were obtained by crystallization from a saturated solution in DCM/EtOAc. **^{1}H NMR** (400 MHz, DMSO-d_6) δ 5.56 (dd, *J* = 10.9, 5.3 Hz, 1H), 5.52 – 5.42 (m, 1H), 4.84 – 4.79 (m, 2H), 4.76 (t, *J* = 4.1 Hz, 1H), 4.36 – 4.26 (m, 1H), 3.99 (dt, *J* = 11.8, 5.0 Hz, 1H), 3.90 – 3.81 (m, 1H), 3.39 – 3.28 (m, 2H), 2.43 – 2.23 (m, 2H), 1.91 – 1.75 (m, 2H), 1.55 – 1.45 (m, 1H), 1.38 (tdd, *J* = 13.3, 11.2, 1.9 Hz, 1H); **^{13}C NMR** (101 MHz, DMSO-d_6) δ 140.5, 121.7, 103.1, 79.4, 79.2, 69.1, 63.4, 36.6, 29.6, 27.2.

TIPS-dcTCO-PNP (ax-6, equ-6)

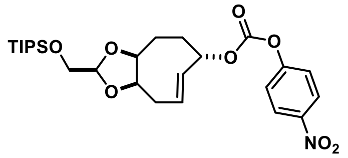

To a solution of **ax-4** (180 mg, 0.505 mmol) in DCM (2 mL) cooled to 0 °C was added 4-dimethylaminopyridine (DMAP, 188 mg, 1.54 mmol) in DCM (1 mL), followed by 4-nitrophenyl chloroformate (PNP-Cl, 247 mg, 1.23 mmol) in DCM (1 mL). After stirring overnight at room temperature, the reaction mixture was concentrated and the residue was purified by column chromatography (90 g SiO_2, DCM/MTBE, 0-5% MTBE gradient elution) to obtain **ax-6** as a pale-yellow oil (109 mg, 41%).**^{1}H NMR** (400 MHz, CD_2Cl_2) δ 8.31 – 8.23 (m, 2H), 7.42 – 7.37 (m, 2H), 6.08 (ddd, *J* = 17.2, 8.6, 6.5 Hz, 1H), 6.01 (ddd, *J* = 16.8, 2.5, 1.1 Hz, 1H), 5.36 (s, 1H), 4.84 (t, *J* = 3.8 Hz, 1H), 4.24 (td, *J* = 6.1, 3.1 Hz, 1H), 4.11 (t, *J* = 6.2 Hz, 1H), 3.90 – 3.83 (m, 2H), 2.68 (ddd, *J* = 15.2, 8.6, 3.1 Hz, 1H), 2.59 (dt, *J* = 15.1, 6.4 Hz, 1H), 2.25 – 2.18 (m, 1H), 2.03 – 1.95 (m, 2H), 1.86 – 1.78 (m, 1H), 1.18 – 1.11 (m, 3H), 1.11 – 1.08 (m, 18H); **^{13}C NMR** (101 MHz, CD_2Cl_2) δ 156.2, 152.2, 146.0, 132.4, 128.1, 125.8, 122.5, 102.9, 84.4, 78.0, 77.4, 65.3, 32.0, 31.5, 26.1, 18.3, 12.5; **HRMS** $[M+H]^+$ calcd. 522.2518 for $C_{26}H_{41}NO_8Si^+$, found 522.2529.

The synthesis of **equ-6** was conducted accordingly: A solution of **equ-4** (58 mg, 0.16 mmol) in DCM (1.5 mL) was reacted with 4-dimethylaminopyridine (DMAP, 79 mg, 0.65 mmol) in DCM (0.5 mL) and 4-nitrophenyl chloroformate (60 mg, 0.30 mmol) in DCM (0.5 mL) to obtain **equ-6** as a pale-yellow solid (75 mg, 89%). Single crystals were obtained by crystallization from a saturated solution in EtOAc. **^{1}H NMR** (400 MHz, CD_2Cl_2) δ 8.26 (d, *J* = 9.0 Hz, 2H), 7.39 (d, *J* = 9.2 Hz, 2H), 6.17 (ddd, *J* = 16.2, 9.8, 5.9 Hz, 1H), 5.56 (dd, *J* = 16.8, 9.5 Hz, 1H), 5.08 (td, *J* = 9.6, 5.4 Hz, 1H), 4.86 (t, *J* = 4.1 Hz, 1H), 4.41 (dt, *J* = 7.9, 4.1 Hz, 1H), 3.87 – 3.80 (m, 1H), 3.70 (d, *J* = 4.1 Hz, 2H), 2.81 – 2.69 (m, 1H), 2.60 (ddd, *J* = 14.6, 6.0, 3.9 Hz, 1H), 2.44 – 2.32 (m, 1H), 1.85 – 1.75 (m, 1H), 1.69 – 1.56 (m, 2H), 1.11 – 1.03 (m, 21H); **^{13}C NMR** (101 MHz, CD_2Cl_2) δ 156.2, 152.4, 146.0, 133.3, 131.4, 125.8, 122.5, 103.2, 85.8, 81.9, 81.8, 66.5, 33.9, 33.1, 33.0, 18.2, 12.5; **HRMS** $[M+H]^+$ calcd. 522.2518 for $C_{26}H_{41}NO_8Si^+$, found 522.2527.

dcTCO derivatives for click kinetics and stability measurements

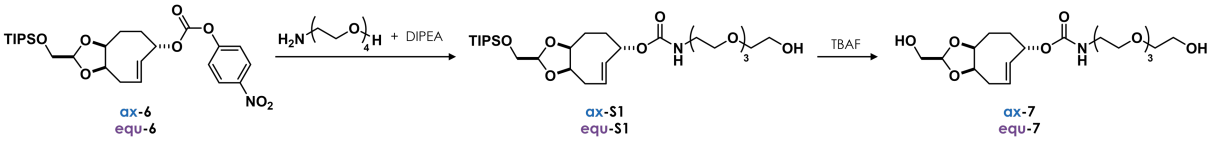

TIPS-dcTCO-PEG$_4$ (ax-S1/equ-S1)

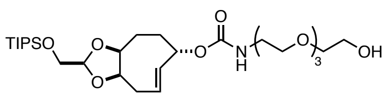

TIPS-dcTCO-PNP (**ax-6**, 76 mg, 0.15 mmol) and amino-dPEG$_4$-OH (40 mg, 0.21 mmol) were dissolved in anhydrous DMF (1.5 mL). DIPEA (38 mg, 51 µL, 0.29 mmol) was added and the mixture was stirred for 90 min at room temperature. The clear yellow solution was concentrated to a third of the volume, acidified by addition of formic acid (20 µL), diluted with water (+0.1% formic acid, 1 mL) and directly subjected to reversed phase preparative HPLC (column: Luna C18, 10 µm, 100 Å, 250 x 10 mm; eluent: 2.5 mM $NH_4^+HCOO^-$ (pH 8.4)/acetonitrile, gradient elution) to obtain **ax-S1** as a colorless oil (50 mg, 59%). **^{1}H NMR** (400 MHz, CD_3CN) δ 5.78 (s, 1H), 5.67 – 5.54 (m, 2H), 5.31 (d, *J* = 11.1 Hz, 1H), 4.86 (t, *J* = 3.9 Hz, 1H), 4.07 (dt, *J* = 10.8, 5.4 Hz, 1H), 3.95 (dd, *J* = 10.8, 5.6 Hz, 1H), 3.68 (d, *J* = 4.2 Hz, 2H), 3.62 – 3.53 (m, 10H), 3.50 (t, *J* = 4.9 Hz, 2H), 3.47 (t, *J* = 5.4 Hz, 2H), 3.22 (q, *J* = 5.6 Hz, 2H), 2.47 – 2.38 (m, 2H), 2.04 – 1.97 (m, 2H), 1.68 – 1.49 (m, 2H), 1.14 – 1.04 (m, 21H); **^{13}C NMR** (101 MHz, CD_3CN) δ 156.8, 135.1, 127.2, 103.1, 84.7, 78.0, 73.3, 72.9, 71.2, 71.1, 70.9, 70.9, 70.6, 65.7, 62.0, 41.4, 32.4, 31.9, 26.2, 18.3, 12.7, **HRMS** $[M+H]^+$ calcd. 576.3562 for $C_{28}H_{55}NO_9Si^+$, found 576.3568.

Accordingly, **equ-S1** was prepared by reacting a solution of **equ-6** (123 mg, 0.236 mmol) in anhydrous DMF (1.5 mL) with NH_2-PEG$_4$-OH (64 mg, 0.33 mmol) and DIPEA (60 mg, 81 µL, 0.46 mmol) obtaining the product as a colorless oil (114 mg, 84%). **^{1}H NMR** (400 MHz, CD_3CN) δ 6.02 (ddd, *J* = 16.1, 9.6, 6.0 Hz, 1H), 5.76 (t, *J* = 5.9 Hz, 1H), 5.46 (dd, *J* = 16.8, 9.5 Hz, 1H), 4.91 (td, *J* = 9.7, 5.3 Hz, 1H), 4.80 (t, *J* = 4.1 Hz, 1H), 4.37 (dt, *J* = 7.3, 4.3 Hz, 1H), 3.80 (ddd, *J* = 10.3, 4.6, 2.3 Hz, 1H), 3.68 (d, *J* = 4.2 Hz, 2H), 3.61 – 3.53 (m, 10H), 3.51 (dd, *J* = 5.7, 3.9 Hz, 2H), 3.47 (t, *J* = 5.5 Hz, 2H), 3.22 (q, *J* = 5.6 Hz, 2H), 2.70 (dddd, *J* = 14.7, 9.0, 7.3, 1.3 Hz, 1H), 2.43 (ddd, *J* = 14.6, 6.0, 4.2 Hz, 1H), 2.17 (dt, *J* = 13.2, 6.6 Hz, 1H), 1.67 (ddd, *J* = 15.0, 7.4, 2.3 Hz, 1H), 1.61 – 1.47 (m, 1H), 1.47 – 1.35 (m, 1H), 1.19 – 1.00 (m, 21H); **^{13}C NMR** (101 MHz, CD_3CN) δ 156.9, 135.7, 129.5, 103.3, 85.8, 82.4, 77.4, 73.2, 71.2, 71.0, 70.9, 70.8, 70.5, 66.8, 61.9, 41.3, 34.4, 33.2, 32.9, 18.2, 12.7; **HRMS** $[M+H]^+$ calcd. 576.3562 for $C_{28}H_{55}NO_9Si^+$, found 576.3563.

dcTCO-PEG$_4$ (ax-7, equ-7)

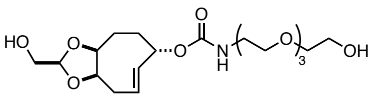

To a solution of **ax-S1** (70 mg, 0.12 mmol) in anhydrous DMSO (0.6 mL) was added TBAF (158 µL, 1.0 M in THF, 0.16 mmol) and the reaction mixture was stirred for 2 h. Subsequently, the solution was concentrated, diluted with buffer (2.5 mM $NH_4^+HCOO^-$, pH 8.4) and purified by reversed phase preparative HPLC (column: Kinetex C18, 5 µm, 100Å; eluent: 2.5 mM $NH_4^+HCOO^-$ (pH 8.4)/acetonitrile, gradient elution) to obtain **ax-7** as a colorless oil (25 mg, 50%). **^{1}H NMR** (600 MHz, DMSO-*d*$_6$) δ 7.16 (t, *J* = 5.8 Hz, 1H), 5.89 – 5.85 (m, 2H), 5.17 – 5.07 (m, 1H), 4.69 (t, *J* = 3.8 Hz, 1H), 4.16 (td, *J* = 5.8, 3.8 Hz, 1H), 4.00 (t, *J* = 6.8 Hz, 1H), 3.53 – 3.46 (m, 14H), 3.42 – 3.37 (m, 4H), 3.11 (q, *J* = 6.0 Hz, 2H), 2.01 (ddd, *J* = 14.6, 11.6, 3.9 Hz, 1H), 1.78 (dtd, *J* = 14.7, 7.1, 1.5 Hz, 1H), 1.71 – 1.64 (m, 2H); **^{13}C NMR** (151 MHz, DMSO-*d*$_6$) δ 155.6, 134.2, 126.0, 102.0, 83.1, 76.3, 72.4, 71.4, 69.9, 69.8, 69.8, 69.6, 69.2, 62.2, 60.2, 40.1, 30.9, 30.7, 25.4; **HRMS** $[M+H]^+$ calcd. 420.2228 for $C_{19}H_{34}NO_9^+$, found 420.2241.

Accordingly, **equ-7** was prepared by reacting a solution of **equ-S1** (61.7 mg, 0.127 mmol) in anhydrous DMSO (0.6 mL) with TBAF (139 µL, 1.0 M in THF, 0.14 mmol) obtaining the product as a colorless oil (19 mg, 42%). **^{1}H NMR** (600 MHz, DMSO-*d*$_6$) δ 7.12 (t, *J* = 5.8 Hz, 1H), 5.99 (ddd, *J* = 16.1, 9.3, 6.2 Hz, 1H), 5.45 (dd, *J* = 16.8, 9.5 Hz, 1H), 4.88 (td, *J* = 9.9, 5.6 Hz, 1H), 4.71 (t, *J* = 4.2 Hz, 1H), 4.34 (dt, *J* = 7.5, 4.6 Hz, 1H), 3.77 (ddd, *J* = 10.5, 4.7, 2.3 Hz, 1H), 3.52 – 3.46 (m, 10H), 3.41 (t, *J* = 5.3 Hz, 2H), 3.38 (t, *J* = 6.0 Hz, 2H), 3.36 (dd, *J* = 4.2, 2.2 Hz, 2H), 3.12 – 3.07 (m, 2H), 2.67 (dt, *J* = 15.7, 8.3 Hz, 1H), 2.32 (ddd, *J* = 14.5, 6.2, 4.7 Hz, 1H), 2.11 (tt, *J* = 11.0, 4.9 Hz, 1H), 1.63 (ddd, *J* = 15.1, 7.7, 2.3 Hz, 1H), 1.50 – 1.41 (m, 1H), 1.39 – 1.30 (m, 1H); **^{13}C NMR** (151 MHz, DMSO) δ 165.6, 155.7, 134.5, 128.4, 102.3, 84.0, 81.0, 76.1, 72.4, 69.8, 69.8, 69.7, 69.6, 69.1, 63.5, 60.2, 33.2, 31.8, 31.8; **HRMS** $[M+H]^+$ calcd. 420.2228 for $C_{19}H_{34}NO_9^+$, found 420.2245.

Release probe

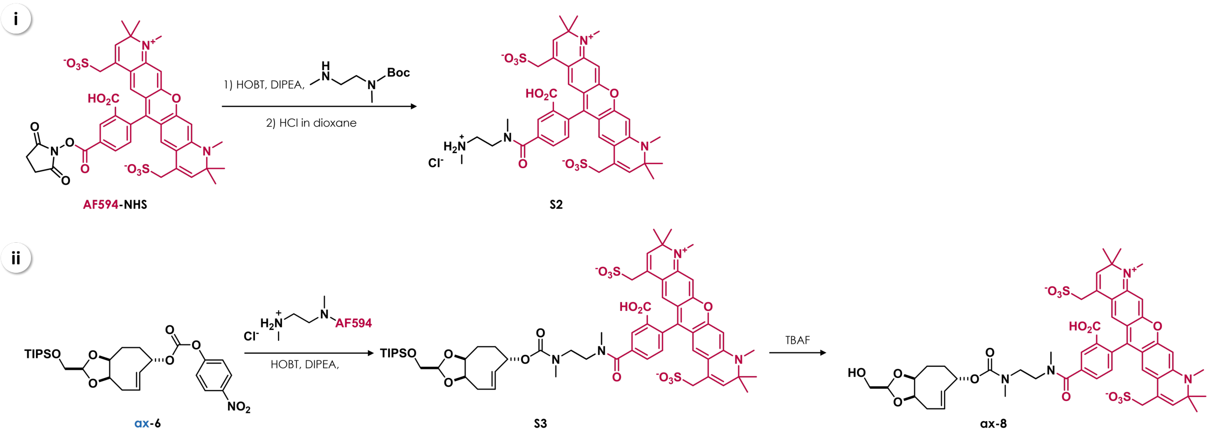

AF594-DMEDA (S2)

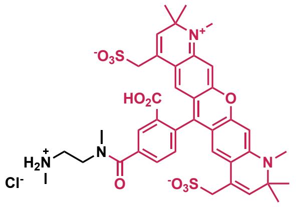

To a solution of Alexa Fluor 594 (AF594)-NHS ester (15 mg, 0.018 mmol) in dry DMSO (1 mL) was added *N*-Boc-*N,N′*-dimethylethylenediamine (10.4 mg, 0.0552 mmol) dissolved in dry DMSO (0.5 mL) followed by the addition of DIPEA (13 µL, 0.070 mmol) and HOBt (2.5 mg, 0.018 mmol). The reaction mixture was stirred at room temperature. LCMS analysis indicated complete conversion after 1 h. Upon addition of formic acid (2.8 µL) the reaction mixture was diluted with MeCN (+ 0.1% formic acid) and directly loaded onto a C18 column (Kinetex C18, 5 µm, 100Å). Reversed phase preparative HPLC (H_2O/acetonitrile + 0.1% formic acid, gradient elution) afforded Boc-protected AF594-DMEDA, which was dissolved in anhydrous DCM (0.5 mL), treated with HCl (76 µL, 0.30 mmol, 4 M in dioxane) and stirred until LCMS indicated complete deprotection. The mixture was concentrated to obtain **S2** (12 mg, 79%) as an HCl salt. **^{1}H NMR** (600 MHz, CD_3OD, mixture of rotamers) δ 8.37 (d, *J* = 83.5 Hz, 1H), 7.97 – 7.82 (m, 1H), 7.52 (s, 1H), 7.31 (d, *J* = 14.7 Hz, 2H), 6.81 (s, 2H), 5.86 (s, 2H), 3.78 – 3.71 (m, 2H), 3.68 – 3.64 (m, 2H), 3.66 (s, 3H), 3.60 – 3.56 (m, 2H), 3.18 (s, 8H), 2.84 – 2.71 (m, 3H), 1.54 (d, *J* = 2.9 Hz, 12H); **^{13}C NMR** (151 MHz, MeOD, mixture of rotamers) δ 173.5, 172.5, 167.7, 163.3, 159.4, 158.4, 158.2, 155.1, 138.9, 138.7, 138.5, 138.2, 136.4, 136.2, 133.3, 132.9, 132.7, 132.3, 132.0, 131.7, 130.4, 125.1, 124.4, 122.9, 115.1, 96.4, 73.6, 72.5, 68.1, 62.2, 61.3, 55.0, 54.6, 47.6, 45.9, 43.7, 39.1, 34.4, 33.4, 29.0, 28.8; **ESI-MS** $[M+H]^+$ calcd. 793.26 for $C_{39}H_{45}N_4O_{10}S_2^+$, found 793.15.

dcTCO-AF594 (ax-8)

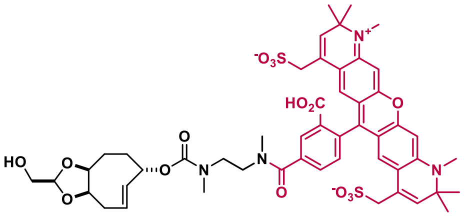

To a solution of **ax-6** (7.0 mg, 0.013 mmol) in anhydrous DMF (0.6 mL) was added AF594-DMEDA (**S2**, 10.1 mg, 0.0128 mmol) in DMSO (600 µL), DIPEA (6.3 mg, 8.5 µL, 0.049 mmol) and HOBt (1.7 mg, 0.012 mmol). After LCMS indicated complete conversion, the reaction mixture was diluted with a 1:1 mixture (v/v) of 500 µL ammonium formate buffer (2.5 mM, pH 8.4) and MeCN. The solution was loaded onto a C18 column (Kinetex C18, 5 µm, 100Å) and purified by reversed phase preparative HPLC (eluent: 2.5 mM $NH_4^+HCOO^-$ (pH 8.4)/acetonitrile, gradient elution). Upon evaporation of the solvent the obtained residue was desalted from $NH_4^+HCOO^-$ on a Biotage Sfär C18 D column by washing with pure water before eluting with 95% MeCN to obtain **S3** as a purple oil (7.7 mg, 54%). **^{1}H NMR** (600 MHz, DMSO-d_6, mixture of rotamers) δ 8.03 – 7.88 (m, 1H), 7.69 – 7.56 (m, 1H), 7.42 – 7.29 (m, 3H), 6.77 – 6.73 (m, 2H), 6.03 –

5.82 (m, 2H), 5.80 – 5.72 (m, 2H), 5.23 – 5.12 (m, 1H), 4.78 – 4.69 (m, 1H), 4.22 – 4.14 (m, 1H), 4.06 – 3.97 (m, 1H), 3.85 – 3.74 (m, 2H), 3.72 – 3.37 (m, 5H), 3.33 – 3.25 (m, 3H), 3.24 – 3.19 (m, 3H), 3.15 – 3.08 (m, 6H), 3.05 – 2.91 (m, 5H), 2.04 – 1.92 (m, 1H), 1.90 – 1.69 (m, 3H), 1.50 – 1.38 (m, 12H), 1.15 – 0.95 (m, 21H); **ESI-MS** $[M]^-$ calcd. 1173.46 for $C_{59}H_{77}N_4O_{15}S_2Si^-$, found 1173.35.

To a solution of **S3** (7.7 mg, 0.0066 mmol) in anhydrous DMSO (0.6 mL) was added TBAF (26 µL, 1.0 M in THF, 0.026 mmol) and the reaction mixture was stirred for 2 h. Subsequently, the solution was diluted with ammonium formate buffer (2.5 mM, pH 8.4) and purified by reversed phase preparative HPLC (column: Kinetex C18, 5 µm, 100Å; eluent: 2.5 mM $NH_4^+HCOO^-$ (pH 8.4)/acetonitrile, gradient elution) followed by desalting on a Biotage Sfär C18 D column (as described for **S3**) to obtain **ax-8** as a purple oil (2.6 mg, 39%). ^{1}**H NMR** (600 MHz, DMSO-d_6, mixture of rotamers) δ 7.96 (d, *J* = 24.8 Hz, 1H), 7.72 – 7.56 (m, 1H), 7.45 – 7.28 (m, 2H), 6.80 – 6.70 (m, 2H), 6.53 (s, 1H), 6.04 – 5.83 (m, 2H), 5.81 – 5.72 (m, 2H), 5.27 – 5.12 (m, 1H), 4.73 – 4.65 (m, 1H), 4.22 – 4.12 (m, 1H), 4.08 – 3.96 (m, 1H), 3.57 – 3.48 (m, 2H), 3.42 – 3.24 (m, 10 H), 3.21 (dd, *J* = 13.9, 6.1 Hz, 2H), 3.15 – 3.08 (m, 6H), 3.05 – 2.91 (m, 4H), 2.13 – 1.94 (m, 1H), 1.91 – 1.69 (m, 3H), 1.55 – 1.32 (m, 12H); **ESI-MS** $[M+2H]^+$ calcd. 1019.34 for $C_{50}H_{59}N_4O_{15}S_2^+$, found 1019.30.

> **Note:** In an initial attempt, without desalting of **S3**, cleavage of the TIPS group required a large excess of TBAF (16 equivalents) and a reaction time of >72 h (presumably due to the formation of an insoluble NH_4F), ultimately resulting in partial isomerization of the TCO (50% CCO).

Synthesis of sulfo-dcTCO-DMEDA-CA4 (15)

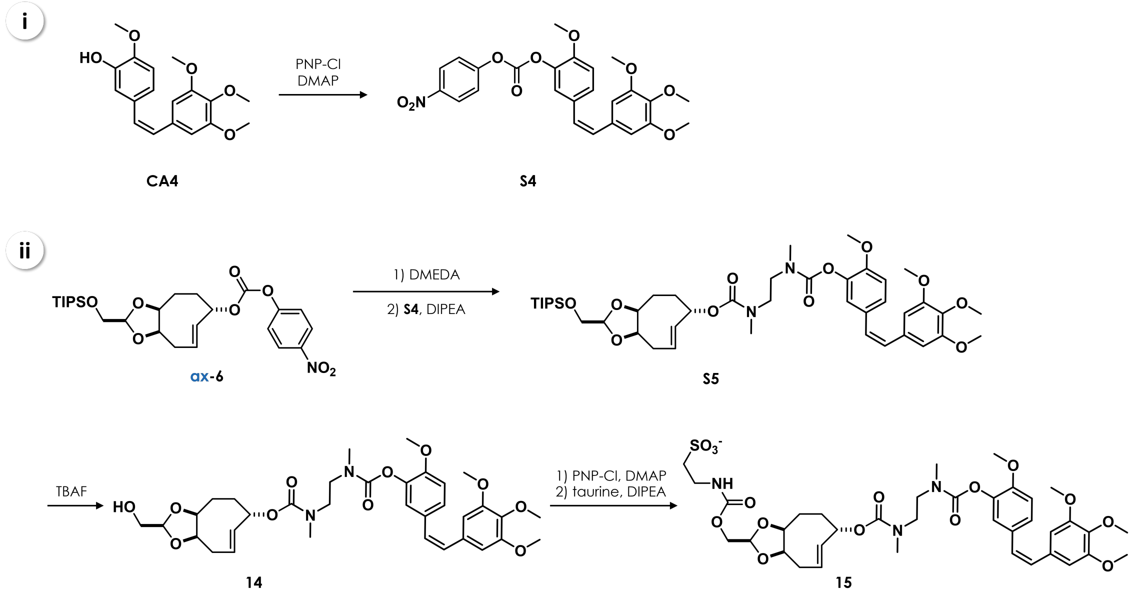

TIPS-dcTCO-DMEDA-CA4 (S5)

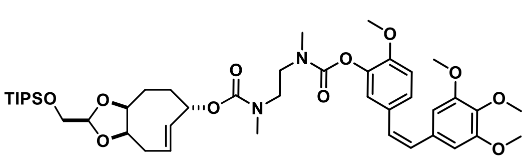

A solution of **ax-6** (18 mg, 0.035 mmol) in anhydrous DCM (4 mL) was slowly added to a solution of DMEDA (30 mg, 0.35 mmol) in a mixture of anhydrous DCM (1.2 mL) and anhydrous DMF (0.6 mL) within 2 hours using a syringe pump. Subsequently, the reaction mixture was stirred for 30 min and then concentrated. Excess DMEDA was removed under vacuum (0.8 mbar, 4 h). The residue was dissolved in anhydrous DMF (1 mL) followed by the addition of

PNP-CA4[5] (**S4**, 29 mg, 0.061 mmol) and DIPEA (24 µL, 0.14 mmol). The reaction mixture was stirred for 2 h at room temperature, concentrated to a third of the volume, acidified by addition of formic acid (20 µL), diluted with water (+0.1% formic acid, 1 mL) and directly subjected to reversed phase preparative HPLC (column: Kinetex C8, 5 µm, 100Å; eluent: H_2O/MeCN + 0.1% formic acid, gradient elution) to obtain **S5** as a colorless oil (10 mg, 35%). **^{1}H NMR** (600 MHz, CD_2Cl_2, mixture of rotamers) δ 7.17 – 7.10 (m, 1H), 7.03 (d, *J* = 11.4 Hz, 1H), 6.85 (d, *J* = 8.5 Hz, 1H), 6.53 (s, 2H), 6.49 – 6.43 (m, 2H), 5.97 – 5.79 (m, 2H), 5.25 (d, *J* = 18.5 Hz, 1H), 4.80 (dt, *J* = 34.2, 4.0 Hz, 1H), 4.19 (dd, *J* = 6.4, 3.6 Hz, 1H), 4.05 (t, *J* = 6.7 Hz, 1H), 3.84 (d, *J* = 3.6 Hz, 2H), 3.80 – 3.77 (m, 3H), 3.75 (s, 3H), 3.68 (s, 6H), 3.58 – 3.39 (m, 4H), 3.08 – 3.04 (m, 1H), 2.99 – 2.90 (m, 5H), 2.63 – 2.56 (m, 1H), 2.51 (tt, *J* = 18.2, 8.9 Hz, 1H), 2.11 – 1.71 (m, 4H), 1.19 – 1.01 (m, 21H); **ESI-MS** $[M+H]^+$ calcd. 813.44 for $C_{43}H_{65}N_2O_{11}Si^+$, found 813.30.

dcTCO-DMEDA-CA4 (14)

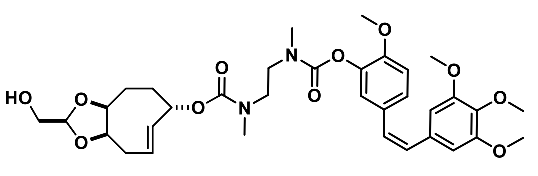

To a solution of **S5** (10 mg, 0,012 mmol) in anhydrous DMSO (0.3 mL) and anhydrous THF (0.1 mL) was added TBAF (25 µL, 1.0 M in THF, 0.025 mmol) and the reaction mixture was stirred for 30 min. Subsequently, the solution was diluted with 1 mL water/MeCN (+0.1% formic acid) and purified by reversed phase preparative HPLC (column: Kinetex C8, 5 µm, 100Å; eluent: H_2O/MeCN + 0.1% formic acid, gradient elution) to obtain **14** as a colorless oil (5 mg, 63%). **^{1}H NMR** (600 MHz, CD_3CN, mixture of rotamers) δ 7.18 – 7.07 (m, 1H), 7.03 – 6.93 (m, 2H), 6.56 (s, 2H), 6.53 – 6.45 (m, 2H), 5.98 – 5.80 (m, 2H), 5.14 (d, *J* = 26.6 Hz, 1H), 4.71 (dt, *J* = 38.3, 3.9 Hz, 1H), 4.18 (dd, *J* = 9.5, 5.0 Hz, 1H), 4.03 (dt, *J* = 13.7, 6.8 Hz, 1H), 3.81 – 3.76 (m, 3H), 3.69 (s, 3H), 3.66 – 3.64 (m, 6H), 3.63 – 3.56 (m, 2H), 3.56 – 3.37 (m, 4H), 3.02 (d, *J* = 12.1 Hz, 1H), 2.96 – 2.83 (m, 5H), 2.58 – 2.40 (m, 2H), 2.05 (td, *J* = 4.7, 3.9, 2.3 Hz, 1H), 1.86 – 1.61 (m, 3H); **^{13}C NMR** (151 MHz, CD_3CN, mixture of rotamers) δ 156.2, 155.9, 155.2, 154.9, 154.8, 154.5, 154.1, 154.0, 152.1, 152.0, 151.9, 141.2, 141.2, 141.1, 138.2, 134.8, 134.7, 133.5, 133.4, 133.4, 130.8, 130.4, 130.3, 129.4, 129.3, 128.1, 128.0, 127.9, 127.3, 127.3, 127.1, 127.0, 124.6, 124.4, 124.4, 113.3, 113.1, 107.1, 107.0, 104.6, 102.7, 102.7, 84.6, 84.5, 78.0, 77.9, 73.8, 73.7, 73.6, 63.6, 63.6, 63.6, 60.8, 60.8, 56.6, 56.5, 56.4, 48.1, 47.9, 47.6, 47.5, 47.3, 47.1, 46.8, 35.6, 35.5, 35.4, 35.3, 35.2, 34.9, 34.7, 32.4, 32.3, 32.2, 31.8, 26.3, 26.3, 26.2, 26.1; **ESI-MS** $[M+H]^+$ calcd. 657.30 for $C_{34}H_{45}N_2O_{11}^+$, found 657.20.

sulfo-dcTCO-DMEDA-CA4 (15)

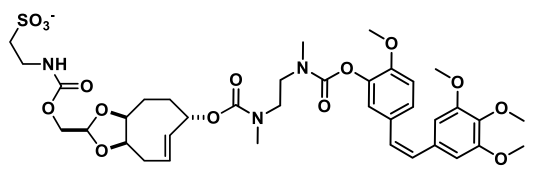

To a solution of **14** (3 mg, 0.005 mmol) in DCM (2 mL) cooled to 0 °C was added DMAP (2.2 mg, 0.018 mmol) dissolved in DCM (0.3 mL), followed by 4-nitrophenyl chloroformate (PNP-Cl, 1.4 mg, 0.0069 mmol) dissolved in DCM (0.3 mL). After stirring at room temperature for 2 h, the reaction mixture was concentrated, and the residue was redissolved in DMF (0.6 mL). After addition of taurine (2.3 mg, 0.018 mmol) and DIPEA (4.8 µL, 0.030 mmol) the reaction mixture was stirred at room temperature overnight. The solution was diluted with 500 µL of a 1:1 mixture (v/v) of ammonium formate buffer (2.5 mM, pH 8.4) and MeCN and directly loaded onto a C18 column (Kinetex C18, 5 µm, 100Å). Reversed phase preparative HPLC (eluent: 2.5 mM $NH_4^+HCOO^-$ (pH 8.4)/acetonitrile, gradient elution) afforded the desired product **15** (0.6 mg, 16%). **^{1}H NMR** (600 MHz, DMSO-d_6, mixture of rotamers) δ 7.17 – 6.94 (m, 4H), 6.60 – 6.53 (m, 2H), 6.53 – 6.28 (m, 2H), 6.01 – 5.69 (m, 2H), 5.32 – 5.02 (m, 1H), 4.99 – 4.67 (m, 1H), 4.26 – 3.83 (m, 4H), 3.83 – 3.59 (m, 12H), 3.59 – 3.35 (m, 4H), 3.30 – 3.19 (m, 2H), 3.14 – 2.72 (m, 6H), 2.59 – 2.52 (m, 2H), 2.47 – 1.35 (m, 6H); **ESI-MS** $[M]^-$ calcd. 806.28 for $C_{37}H_{48}N_3O_{15}S^-$, found 806.25.

Synthesis of rTCO-DMEDA-CA4 (16)

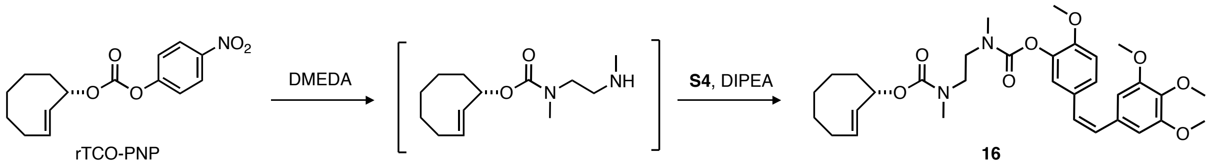

rTCO-DMEDA-CA4 (16)

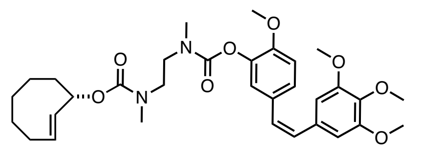

A solution of rTCO-PNP (axial isomer, 9.2 mg, 31.6 µmol) in DCM (1.8 mL) was added via syringe pump (30 µL/min) to a solution of DMEDA (34.1 µL, 27.9 mg, 317 µmol) in CH_2Cl_2 (550 µL) and DMF (180 µL) at 0 °C. After complete addition, the solution was stirred at 0 °C for 1 h. Excess of DMEDA was removed under high vacuum (3 h) to obtain crude rTCO-DMEDA. This intermediate was dissolved in CH_2Cl_2 (480 µL) and added to a solution of PNP-CA4[5] (**S4**, 12.3 mg, 25.5 µmol) in DMF (240 µL) at room temperature. LCMS indicated complete conversion after 1 h. Upon purification by preparative HPLC (hexane/EtOAc gradient elution) **16** was obtained as an off-white solid (9.0 mg, 62 %). ^{1}H NMR (600 MHz, CD_2Cl_2, mixture of rotamers) δ 7.17 – 7.08 (m, 1H), 7.07 – 6.97 (m, 1H), 6.85 (d, *J* = 8.5 Hz, 1H), 6.53 (s, 2H), 6.50 – 6.42 (m, 2H), 5.84 – 5.71 (m, 1H), 5.60 – 5.47 (m, 1H), 5.30 (s, 1H), 3.81 – 3.77 (m, 3H), 3.75 (s, 3H), 3.68 (s, 6H), 3.65 – 3.40 (m, 4H), 3.09 – 2.90 (m, 6H), 2.48 – 2.27 (m, 1H), 2.11 – 1.59 (m, 6H), 1.54 – 1.29 (m, 1H), 1.15 – 0.97 (m, 1H), 0.87 – 0.73 (m, 1H); ^{13}C NMR (151 MHz, CD_2Cl_2, mixture of rotamers) δ 155.88, 155.82, 155.54, 154.65, 154.57, 154.41, 153.46, 151.39, 151.33, 140.74, 140.67, 140.59, 137.67, 132.91, 132.87, 132.05, 131.94, 130.32, 130.29, 129.70, 129.66, 128.96, 127.46, 127.42, 127.37, 127.35, 126.37, 124.25, 124.18, 124.12, 123.99, 112.40, 112.28, 112.19, 106.36, 74.82, 74.71, 74.65, 60.82, 56.39, 56.33, 56.29, 56.23, 56.19, 48.16, 47.91, 47.62, 47.52, 47.14, 46.81, 46.68, 41.49, 41.17, 41.13, 36.42, 36.31, 36.23, 36.19, 35.80, 35.74, 35.55, 35.50, 35.41, 35.12, 34.84, 29.48, 29.43, 24.75, 24.66, 24.60, 24.54; HRMS $[M+H]^+$ calcd. 583.3014 for $C_{32}H_{43}N_2O_8^+$, found 583.3014.

2) Click kinetics

Stock solutions and solvents

Stock solutions of dcTCO-PEG$_4$ (**ax-7**, **equ-7**) and rTCO-PEG$_4$ (**S6**)[6] were prepared in DMSO at a concentration of 100 mM, so that the final concentration of DMSO upon dilution in buffer was never exceeding 1%. The exact concentration was determined by absorbance titration with 2Pyr$_2$ (**13**) (extinction coefficient at 520 nm: 433.2 $M^{-1}cm^{-1}$), quantifying the decrease in tetrazine absorbance upon reaction with TCO. Stock solutions of DMT (**9**),[7] MBA (**10**), PymK (**11**),[8] HBA (**12**), and 2Pyr$_2$ (**13**) were prepared in DMSO at a concentration of 10 mM.

Sample preparation

The initial DMSO stocks of **ax-7**, **equ-7** and **S6** were diluted with PBS (pH 7.4, 10 mM) to a final TCO concentration of 1 mM, whereas the Tz stock solutions were diluted to a concentration of 0.1 mM.

Stopped-flow spectrophotometry

Stopped-flow measurements were performed using an SX20-LED stopped-flow spectrophotometer (Applied Photophysics) equipped with a 535 nm LED (optical pathlength 10 mm, full width half-maximum 34 nm) to monitor the characteristic Tz absorption (520-540 nm). The reagent syringes were loaded with Tz and TCO solutions and the instrument was primed. Measurements were done in triplicates to sextuplicates at 37 °C and recorded automatically at the time of acquisition. Data was analyzed by fitting an exponential decay using Prism 6 (Graphpad) and the resulting pseudo-first-order rate constants were divided by the TCO concentration to calculate the second-order rate constants (Table S1).

Table S1. Second-order rate constants (k, n = 3-6, mean ± SD) determined by stopped-flow spectrophotometry, showing similar IEDDA reactivity of dcTCO and rTCO in PBS at 37°C.

TCO	tetrazine (Tz)	k ($M^{-1}s^{-1}$)
**rTCO-PEG$_4$**	DMT (**9**)	82 ± 9
**equ-7**	DMT (**9**)	2.5 ± 0.1
**ax-7**	DMT (**9**)	74 ± 6
**ax-7**	MeBA (**10**)	370 ± 30
**rTCO-PEG$_4$**	PymK (**11**)	1420 ± 160
**ax-7**	PymK (**11**)	1320 ± 120
**ax-7**	HBA (**12**)	7360 ± 660
**equ-7**	2Pyr$_2$ (**13**)	210 ± 10
**ax-7**	2Pyr$_2$ (**13**)	7940 ± 700

3) Release experiments

Instrument and solvents

All release measurements were performed on a Nexera X2® UHPLC system (Shimadzu) with a temperature-controlled autosampler at 37 °C. For buffered LCMS conditions, the aqueous solvent was prepared by addition of 625 µL of 10 M ammonium formate (BioUltra, Sigma-Aldrich) to 2.5 L of HPLC-grade water followed by adjusting the pH to 8.4 by addition of 100 µL of 25% aqueous ammonia (for HPLC, LiChropur, Merck). The pH of this volatile buffer declines over time and was thus freshly prepared each day. HPLC-grade acetonitrile was used without any additives.

Stock solutions

A stock solution of dcTCO-AF594 (**ax-8**) in DMSO was prepared at a concentration of 5 mM. The exact concentration was determined by absorbance measurements of samples made by serial dilutions of the stock solution in water (extinction coefficient of AF594-amine at 590 nm in PBS: 122 000 $M^{-1}cm^{-1}$). Tz (**9-13**) stock solutions were prepared in DMSO at a concentration of 10 mM.

Endpoint measurements

For endpoint measurements, the dcTCO-AF594 (**ax-8**) stock solution was diluted with PBS (10 mM, pH 7.4) to a concentration of 50 µM in an LCMS sample vial. Tz stock solutions were diluted with PBS to a concentration of 100 µM. To initiate the release reaction the Tz solution was added to the TCO sample to reach final concentrations of 25 µM TCO and 50 µM Tz. The samples were incubated at 37 °C and measured after 48 h. All measurements were conducted in triplicates.

Release kinetics measurements

dcTCO-AF594: The dcTCO-AF594 (**ax-8**) stock solution was diluted with PBS (10 mM, pH 7.4) to a concentration of 50 µM in an LCMS sample vial. Tz stock solutions were diluted with PBS to a concentration of 80 µM. To initiate the release reaction the Tz solution was added to the TCO sample to reach final concentrations of 25 µM TCO and 40 µM Tz. Monitoring of the reaction at 37 °C was performed by serial HPLC measurements in triplicates. The released AF594-amine was detected using an RF-20Axs fluorescence detector and an LCMS-2020 mass spectrometer (ESI). Quantitative analysis of the product was done based on extracted fluorescence chromatograms integrated at 594 nm.

TCO-CA4: Stock solutions of dcTCO-DMEDA-CA4 (**14**) and rTCO-DMEDA-CA4 (**16**) were prepared in DMSO at a concentration of 10 mM. The exact TCO concentration was determined by absorbance titration (535 nm) with a freshly prepared $2Pyr_2$ (**13**) stock solution in DMSO using a Thermo Fisher Scientific NanoDrop One^C Microvolume UV-Vis Spectrophotometer in cuvette mode at 25 °C. The stock solutions of dcTCO-DMEDA-CA4 (**14**) and rTCO-DMEDA-CA4 (**16**) were diluted with PBS (10 mM, pH 7.4) to a concentration of 100 µM in an LCMS sample vial. A DMT (**9**) stock solution (20 mM) was diluted with PBS to a concentration of 160 µM. To initiate the release reaction the Tz solution was added to the TCO sample to reach final concentrations of 50 µM TCO and 80 µM Tz. Monitoring of the reaction at 37 °C was performed by serial HPLC measurements in triplicates. Released **CA4** was detected using a PDA detector and an LCMS-2020 mass spectrometer (ESI), and quantified via external calibration (extracted UV chromatograms at 297 nm). Results are shown in Figure S1.

External CA4 calibration: A **CA4** stock solution (20 mM) was prepared in DMSO and diluted with PBS (+10% DMSO) to a concentration of 100 µm. **CA4** standard solutions (1–75 µM) were prepared by serial dilution using PBS (+10% DMSO). All measurements were conducted in triplicates.

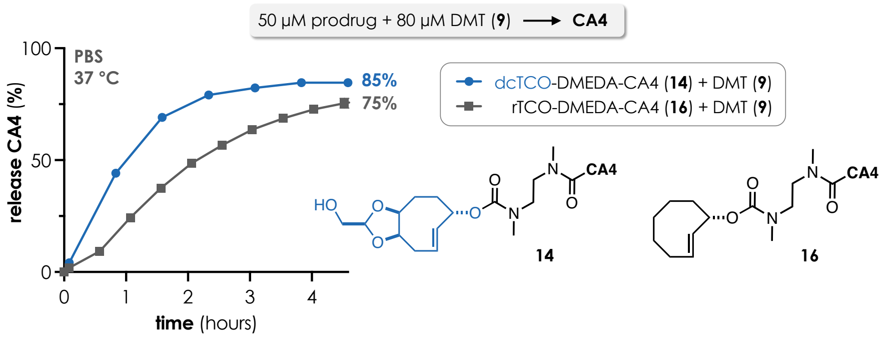

Figure S1. Click-to-release of **CA4** upon reaction of the TCO-caged prodrugs **14** and **16** with DMT (**9**) shows faster elimination of dcTCO in comparison to rTCO.

4) Stability measurements

Stock solutions of $rTCO\text{-}PEG_4$ (**S6**)[6] and $dcTCO\text{-}PEG_4$ (**ax-7**) were prepared in DMSO at a concentration of 200 mM. Exact TCO concentrations were determined by absorbance measurements upon titration with a 15 mM $2Pyr_2$ (**13**) stock solution in DMSO at 520 nm. The stability of the TCOs was evaluated in PBS buffer (10 mM, pH 7.4). To this end, the 200 mM TCO stock solutions were diluted into PBS at a concentration of 200 µM. The blank solution of each medium was spiked with the same amount of DMSO and treated in the same way as the TCO solutions. All samples and controls were incubated at 37 °C in the dark. At each time point (0, 6, 12, 48, and 96 h) three aliquots (each 1 mL) of each solution were taken, spiked with an excess of a 15 mM stock solution of $2Pyr_2$ (**13**) in DMSO and mixed. After an incubation time of 15 minutes in a quartz cuvette, the remaining tetrazine absorbance at 535 nm was determined at 25°C. This procedure was repeated twice (standard addition). The measured absorbances were plotted against the Tz concentration that would be present without any reaction with TCO. Extrapolation of this standard addition plot gives the concentration of the consumed Tz and thus the remaining concentration of the TCO concentration at the intersection with the x-axis. Both compounds showed >90% stability in PBS at 37°C for at least 4 days (dcTCO: 99%, rTCO: 93%, Fig. S2).

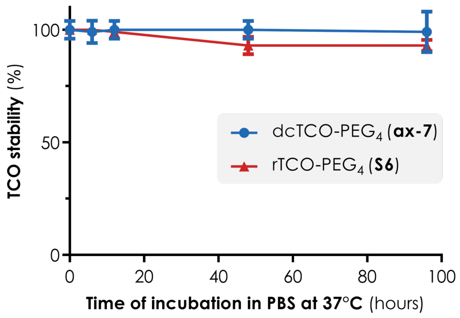

Figure S2. Stability of dcTCO and rTCO in PBS.

5) Log*P* calculations

Log*P* values were calculated at pH 7.4 (*c*Log$P_{7.4}$, Chemicalize, ChemAxon) for a range of dcTCO derivatives including hydrophobic and hydrophilic substituents, along with selected cytotoxic drugs and compared to values calculated for analogous rTCO and cTCO compounds (Fig. S3). As a click-removable tag dcTCO shows an approx. 55-fold reduced lipophilicity in comparison to rTCO with an average ΔcLog$P_{7.4}$ of –1.74 , and an average ΔcLog$P_{7.4}$ of –1.67 compared to cTCO. As a click-cleavable linker still a significantly lower lipophilicity (approx. 4-fold) of dcTCO conjugates compared to cTCO-analogs was found (average ΔcLog$P_{7.4}$ of –0.57, Fig. S3).

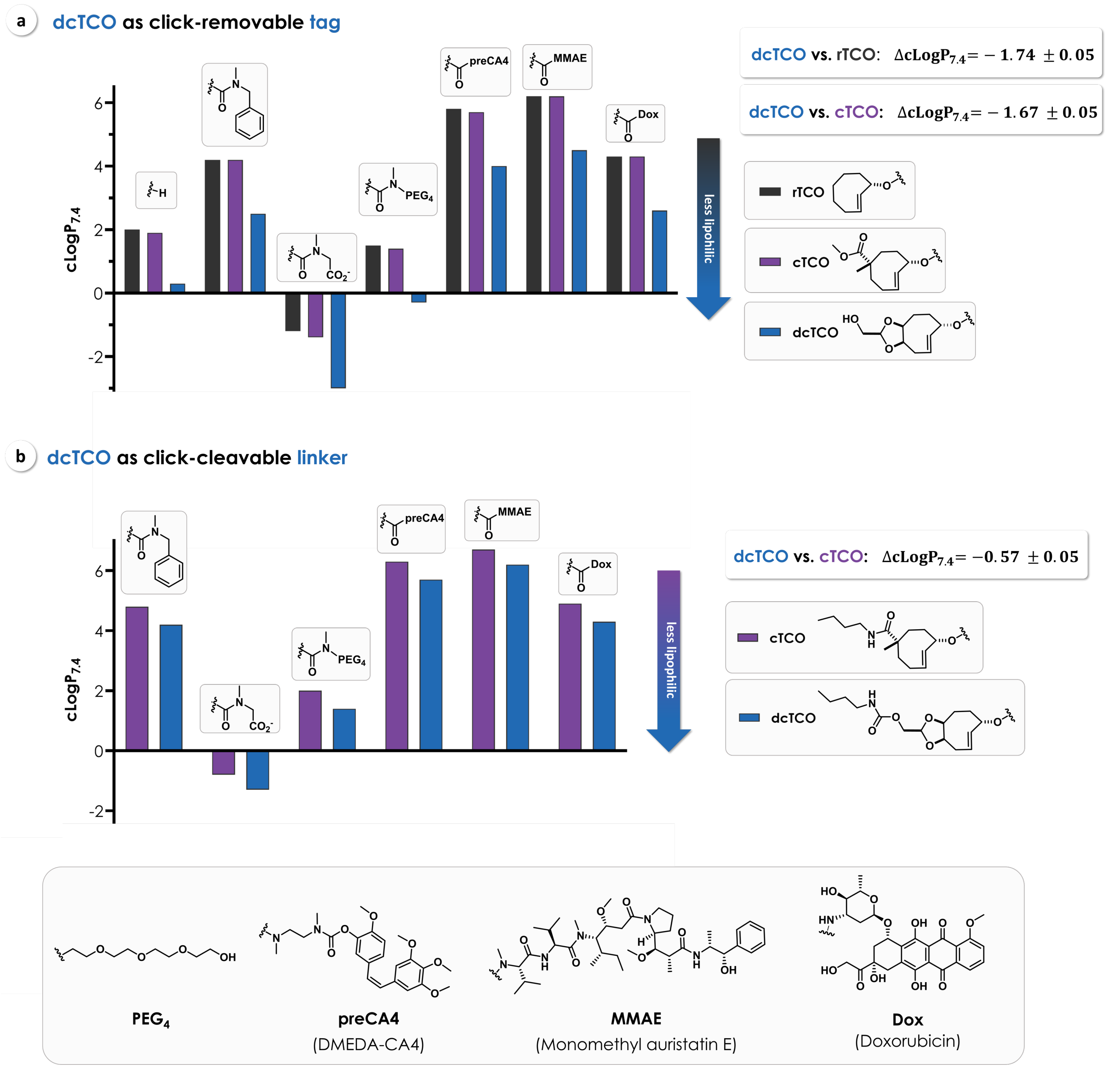

Figure S3. Calculated log*P* values at pH 7.4 for selected compounds (including less and more hydrophilic structures, and the drugs MMAE and doxorubicin) modified with the *trans*-cyclooctenes rTCO, cTCO, and dcTCO, respectively, show (a) a substantially (approx. 55-fold) reduced lipophilicity of dcTCO as a click-removable tag in comparison to rTCO and cTCO, and (b) a less pronounced but still significant (approx. 4-fold) difference in the lipophilicities of dcTCO-conjugates and respective cTCO-analogs.

6) Cell experiments

Cell viability assays

HT1080 human fibrosarcoma cells (ATCC) were cultivated in EMEM (Minimum Essential Medium Eagle, with Earle's salts, L-glutamine and sodium bicarbonate; Sigma Aldrich) supplemented with 10% fetal bovine serum and 1% antibiotic/antimycotic solution (100X, Sigma-Aldrich) at 37 °C and 5% CO_2. HT1080 cells were seeded into 96-well plates (triplicates for each group) at 10.000 cells per well and allowed to grow overnight.

The medium was removed and a dilution series of sulfo-dcTCO-DMEDA-CA4 (**15**) or the parent drug **CA4** in growth medium (10 µM, 2 µM, 0.4 µM, 0.08 µM, 0.016 µM, 0.0032 µM, 0.00064 µM, 0.000128 µM, 0.0000256 µM) was added to the cells (0.1% final DMSO concentration). For release experiments the same concentrations of **15** were used, while a stock solution of DMT (**9**) was added to obtain a final Tz concentration of 10 µM. Incubation was carried out for 72 h.

Cell viability was assessed by replacing the medium with 100 µL of PrestoBlue solution (Invitrogen, 1:9 in growth medium) followed by incubation for 30 minutes at 37 °C. Read-out of the fluorescence signal was carried out using a PerkinElmer EnSpire Multimode Plate Reader and data processing was done in GraphPad Prism.

Following the same procedure, cells were treated with DMT (**9**) or 1,3-dimethylimidazolidin-2-one (= byproduct of the self-immolation process) with concentrations of up to 10 µM, revealing no significant effect on cell viability.

Cell imaging – Fluorescence microscopy

HT1080 cells were seeded into a 96-well plate at 10,000 cells per well and allowed to grow overnight. The medium was removed, and the cells were treated with a 50 nM solution of sulfo-dcTCO-DMEDA-CA4 (**15**) in media. *In situ* click-to-release was initiated by addition of DMT (**9**) at a final concentration of 10 µM. As controls, cells were left untreated or incubated with either the parent drug **CA4** (50 nM) or 10 µM DMT (**9**). After an incubation time of 6 h cells were stained with SiR-tubulin (a fluorogenic, cell permeable and highly specific probe for microtubules).[9] An 11X stock solution of the probe was directly added to the growth medium to obtain a final concentration of 1 µM and incubation was carried out for 1 h. Subsequently, the medium was removed, and cells were stained with Hoechst 33342 nuclear dye (Invitrogen, 5 µM in growth medium) for 10 minutes and washed once with PBS. Multichannel imaging of the cells was carried out in FluoroBrite DMEM medium (Gibco) on an Olympus IX82 microscope (Fig. S4).

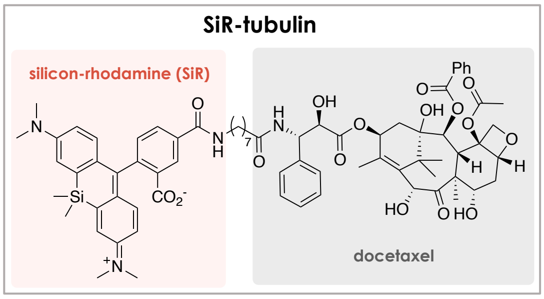

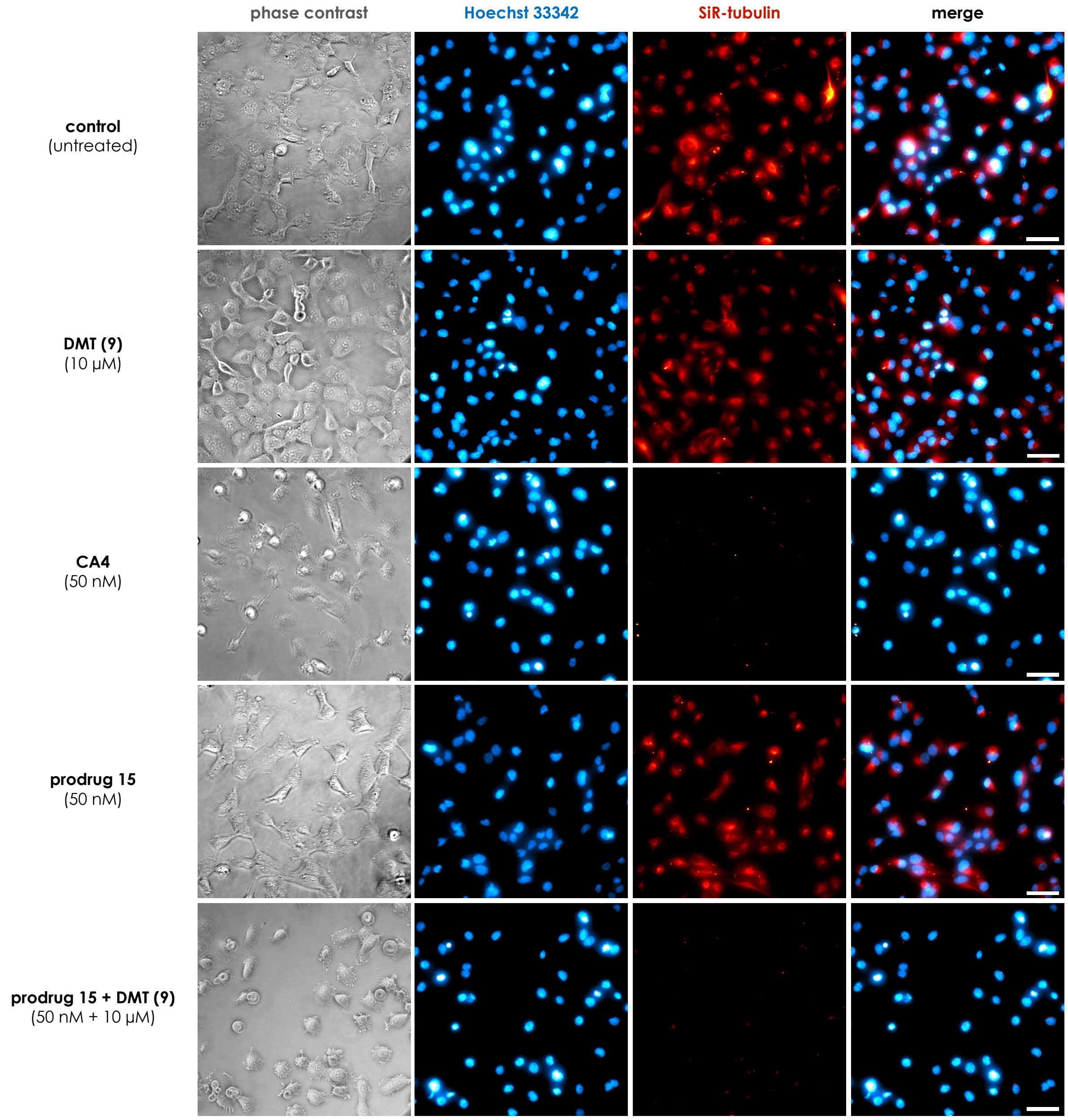

Figure S4. Cell imaging via fluorescence microscopy (scale bars: 50 µm) upon staining with Hoechst 33342 (blue, nuclei) and SiR-tubulin[9] (red, microtubules) shows comparable depletion of tubulin signals after 6 h treatment with **CA4** (50 nM) or bioorthogonal activation of prodrug **15** (50 nM) by *in situ* reaction with DMT (**9**). No significant change (compared to untreated cells) was observed after treatment with DMT (**9**) or prodrug **15**.

7) Single crystal X-ray diffraction

X-ray diffraction data of **5** and **equ-6** (CCDC 2108332, CCDC 2108333) were collected at T = 100 K in a dry stream of nitrogen on a Bruker Kappa APEX II diffractometer system using graphite-monochromatized Mo-$K\alpha$ radiation (λ = 0.71073 Å) and fine sliced φ- and ω-scans. Crystals of **5** were systematically twinned. Two domains related by twofold rotation about **a*** or reflection at (100) were identified using the RLATT tool. The actual twin law could not be determined experimentally owing to negligible resonant scattering and a non-centrosymmetric crystal structure. Data were reduced to intensity values with SAINT and an absorption correction was applied with the multi-scan approach implemented in SADABS or TWINABS (Bruker computer programs: APEX3, RLATT, SAINT, SADABS and TWINABS). The structures were solved by the dual-space approach implemented in SHELXT[10] and refined against F^2 with SHELXL.[11]

dcCCO (**5**) crystallized in space group P21 (enantiomorphic crystal) and **equ-6** crystallized in space group P21/c (racemic crystal). The absolute structure of the enantiomorphic crystal of **5** was not determined owing to a lack of resonant scatterers.

Non-hydrogen atoms were refined with anisotropic displacement parameters. H atoms attached to C were generally placed in calculated positions and thereafter refined as riding on the parent atoms. Difference Fourier maps suggested that the H atoms of the ethylene fragment (CH=CH) in **equ-6** were placed erroneously. These H atoms were therefore refined freely. The H atoms of the OH groups were likewise located from difference Fourier maps and refined freely. Molecular graphics were generated with the program MERCURY.[12] Ellipsoids were plotted at the 50% probability levels (see manuscript, Fig. 2d and Fig. 3b). Detailed CheckCIF-reports are provided in PDF-format as supplementary material.

8) Computational studies

Computational investigations

Density functional theory investigations were performed using Gaussian 16 RevA.03. ωB97X-D[13] was used as the functional combined with the def2-SVP or def2-TZVP basis set[14] for large and small systems, respectively. Solvent effects were included using the SMD model. Conformer searches were performed using CREST.[15] Minima were confirmed by having no imaginary frequencies. A quasi-harmonic correction was applied to entropy calculations by setting all frequencies below 100 cm^{-1} to 100 cm^{-1} using the GoodVibes script.[16] Structures for all conformers are supplied as *.xyz files.

Conformational analysis of compound 2

A conformational search was conducted on structure **2** to determine the ring conformer in DCM solution. The lowest 20 conformers from the CREST conformational search were further optimized using ωB97X-D/def2SVP-SMD(DCM).

Two groups of structures with unique cyclooctene ring conformers could be identified. 16 of the 20 structures belong to a twist-boat-like conformer (group 1) with a Boltzmann-weighted share of 99% in the investigated ensemble. Four structures belong to twist-chair-like conformer (group 2) with a Boltzmann-weighted share of 1%. The lowest energy conformer of this group is 1.6 kcal/mol higher in energy than the lowest energy conformer of the first group. Figure S5 shows the lowest energy conformer for each group and the energy distributions within the different groups relative to the lowest energy conformer.

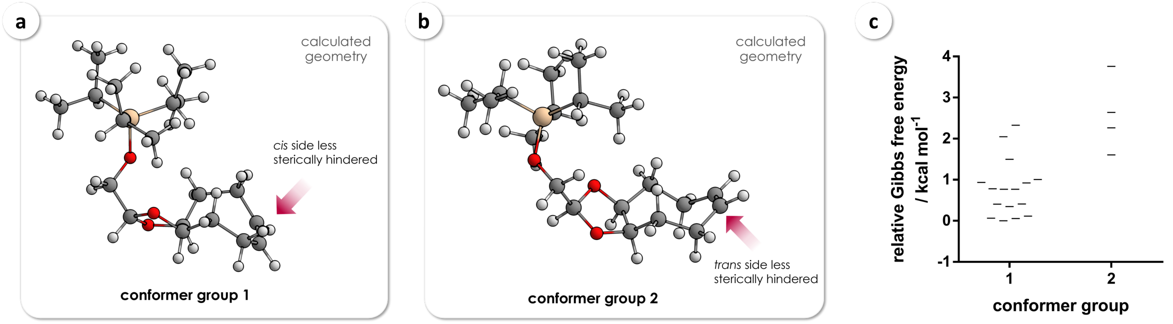

Figure S5. (a) Lowest energy conformer of conformer group 1. (b) Lowest energy conformer of conformer group 2. (c) Relative Gibbs free energies for investigated conformers.

Conformer group 1 only allows a reaction on the double bond *cis* to the dioxolane ring, while for conformer group 2 both *cis* and *trans* are viable with less steric hindrance on the *trans* side. Table S2 lists the energies of all conformers.

Table S2. Energies, relative Gibbs free energies, contribution to the investigated ensemble, and conformer group for all 20 investigated conformers of **2**.

structure	E (hartree)	ZPE (hartree)	H (hartree)	G (hartree)	relative energy (kcal mol-1)	contribution	conformer group
**2_conf01**	-1259.86	0.529845	-1259.31	-1259.386205	0.0	15%	1
**2_conf02**	-1259.86	0.529662	-1259.31	-1259.386116	0.1	13%	1
**2_conf03**	-1259.86	0.529668	-1259.31	-1259.386104	0.1	13%	1
**2_conf04**	-1259.86	0.52945	-1259.31	-1259.386024	0.1	12%	1
**2_conf05**	-1259.86	0.530257	-1259.31	-1259.385649	0.3	8%	1
**2_conf06**	-1259.86	0.52953	-1259.31	-1259.385556	0.4	7%	1
**2_conf07**	-1259.86	0.529141	-1259.31	-1259.385551	0.4	7%	1
**2_conf08**	-1259.86	0.530343	-1259.31	-1259.384979	0.8	4%	1
**2_conf09**	-1259.86	0.529836	-1259.31	-1259.384978	0.8	4%	1
**2_conf10**	-1259.86	0.529848	-1259.31	-1259.384961	0.8	4%	1
**2_conf11**	-1259.86	0.529695	-1259.3	-1259.384733	0.9	3%	1
**2_conf12**	-1259.86	0.530477	-1259.31	-1259.384713	0.9	3%	1
**2_conf13**	-1259.86	0.530182	-1259.31	-1259.384603	1.0	3%	1
**2_conf14**	-1259.86	0.529875	-1259.3	-1259.383819	1.5	1%	1
**2_conf15**	-1259.86	0.529222	-1259.3	-1259.383644	1.6	1%	2
**2_conf16**	-1259.86	0.529991	-1259.3	-1259.382939	2.0	0%	1
**2_conf17**	-1259.86	0.529336	-1259.3	-1259.382593	2.3	0%	2
**2_conf18**	-1259.86	0.530364	-1259.3	-1259.382494	2.3	0%	1
**2_conf19**	-1259.86	0.529368	-1259.3	-1259.381997	2.6	0%	2
**2_conf20**	-1259.86	0.52953	-1259.3	-1259.380212	3.8	0%	2

Conformational analysis of dcTCO

The dcTCO model structures **ax-dcTCO*** and **equ-dcTCO*** (Figure S6) were investigated focusing on the ring conformation in aqueous solution. Therefore, a conformer search was conducted following by optimization using ωB97X-D/def2TZVP-SMD(water).

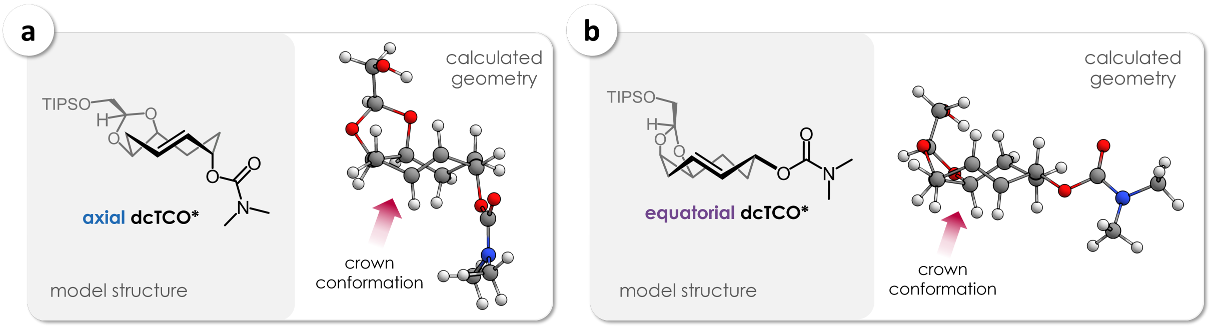

Figure S6. (a) Model structure **ax-dcTCO***and its lowest energy conformer. (b) Model structure **eq-dcTCO*** and its lowest energy conformer.

For **ax-dcTCO*** 15 conformers were investigated. 11 of these conformers, including all low energy structures, show a crown conformation of the *trans*-cyclooctene ring structure. Structures not in crown-conformation are at least 3.3 kcal/mol higher than the lowest energy conformer and do not contribute to the ensemble. The lowest energy conformer is shown in Figure S5a. Table S3 lists the energies of all conformers.

Table S3. Energies, relative Gibbs free energies, contribution to the investigated ensemble, and conformer group for all 15 investigated conformers of **ax-dcTCO***.

structure	E (hartree)	ZPE (hartree)	H (hartree)	G (hartree)	relative energy (kcal mol^{-1})	contribution	crown conformer
**ax-dcTCO_conf01**	-938.956657	0.344651	-938.592178	-938.656808	0.0	35%	yes
**ax-dcTCO_conf02**	-938.955078	0.344298	-938.590771	-938.655735	0.7	11%	yes
**ax-dcTCO_conf03**	-938.955281	0.344478	-938.590863	-938.655718	0.7	11%	yes
**ax-dcTCO_conf04**	-938.953978	0.343858	-938.589882	-938.655403	0.9	8%	yes
**ax-dcTCO_conf05**	-938.955079	0.344640	-938.590558	-938.655311	0.9	7%	yes
**ax-dcTCO_conf06**	-938.954130	0.344211	-938.589794	-938.655131	1.1	6%	yes
**ax-dcTCO_conf07**	-938.954974	0.344771	-938.590323	-938.655020	1.1	5%	yes
**ax-dcTCO_conf08**	-938.953962	0.344122	-938.589701	-938.655011	1.1	5%	yes
**ax-dcTCO_conf09**	-938.954073	0.344221	-938.589760	-938.654976	1.1	5%	yes
**ax-dcTCO_conf10**	-938.953914	0.344301	-938.589529	-938.654603	1.4	3%	yes
**ax-dcTCO_conf11**	-938.953396	0.344140	-938.589134	-938.654414	1.5	3%	yes
**ax-dcTCO_conf12**	-938.949932	0.343798	-938.585705	-938.651509	3.3	0%	no
**ax-dcTCO_conf13**	-938.950767	0.344629	-938.586218	-938.650945	3.7	0%	no
**ax-dcTCO_conf14**	-938.949810	0.344334	-938.585391	-938.650565	3.9	0%	no
**ax-dcTCO_conf15**	-938.941739	0.344206	-938.577313	-938.642908	8.7	0%	no

For **equ-dcTCO*** eleven conformers were investigated. 8 of those conformers, including all low energy structures, show a crown conformation of the *trans*-cyclooctene ring structure. One structure with a relative energy of +1.1 kcal/mol is in a twist-boat-chair like conformation and contributes 5% to the Boltzmann-weighted ensemble. Two high energy conformers at +4.6 and +4.7 kcal mol^{-1}, respectively, are in a twist-chair conformation, but do not contribute in any significant way. The lowest energy conformer is shown in Figure S5b. Table S4 lists the energies of all conformers.

Table S4. Energies, relative Gibbs free energies, contribution to the investigated ensemble, and conformer group for all 11 investigated conformers of **equ-dcTCO***.

structure	E (hartree)	ZPE (hartree)	H (hartree)	G (hartree)	relative energy (kcal mol^{-1})	contribution	crown conformer
**equ-dcTCO_conf01**	-938.958547	0.343881	-938.594561	-938.659942	0.0	36%	yes
**equ-dcTCO_conf02**	-938.958342	0.343995	-938.594241	-938.659650	0.2	26%	yes
**equ-dcTCO_conf03**	-938.956802	0.343319	-938.593110	-938.659195	0.5	16%	yes
**equ-dcTCO_conf04**	-938.957783	0.343896	-938.593765	-938.659151	0.5	16%	yes
**equ-dcTCO_conf05**	-938.957972	0.343325	-938.595920	-938.658132	1.1	5%	no
**equ-dcTCO_conf06**	-938.955165	0.344485	-938.590836	-938.655646	2.7	0%	yes
**equ-dcTCO_conf07**	-938.953857	0.343924	-938.589828	-938.655105	3.0	0%	yes
**equ-dcTCO_conf08**	-938.953622	0.344299	-938.589284	-938.654361	3.5	0%	yes
**equ-dcTCO_conf09**	-938.953073	0.344111	-938.588890	-938.654049	3.7	0%	yes
**equ-dcTCO_conf10**	-938.951539	0.344274	-938.587162	-938.652561	4.6	0%	no
**equ-dcTCO_conf11**	-938.951731	0.344467	-938.587176	-938.652465	4.7	0%	no

9) NMR spectra and chromatograms

Compound **2**, ^{1}H-NMR

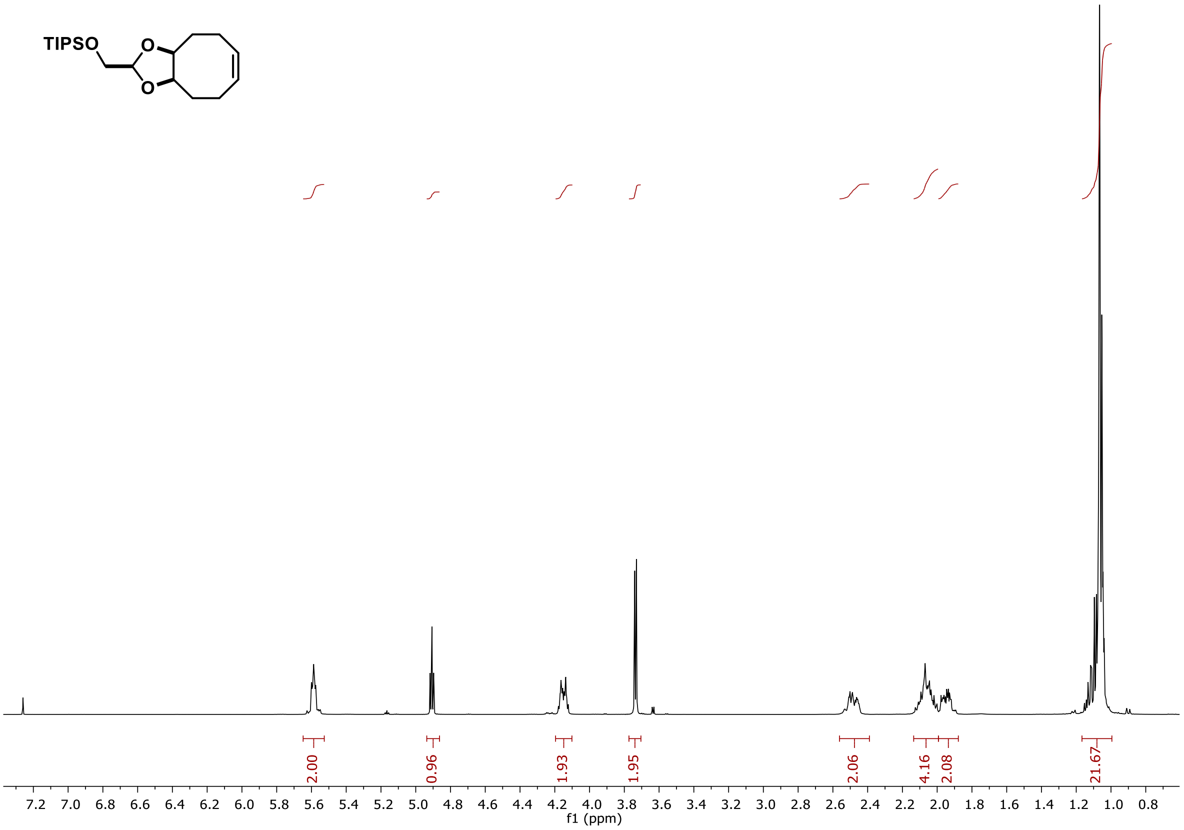

Compound **2**, ^{13}C-NMR (APT)

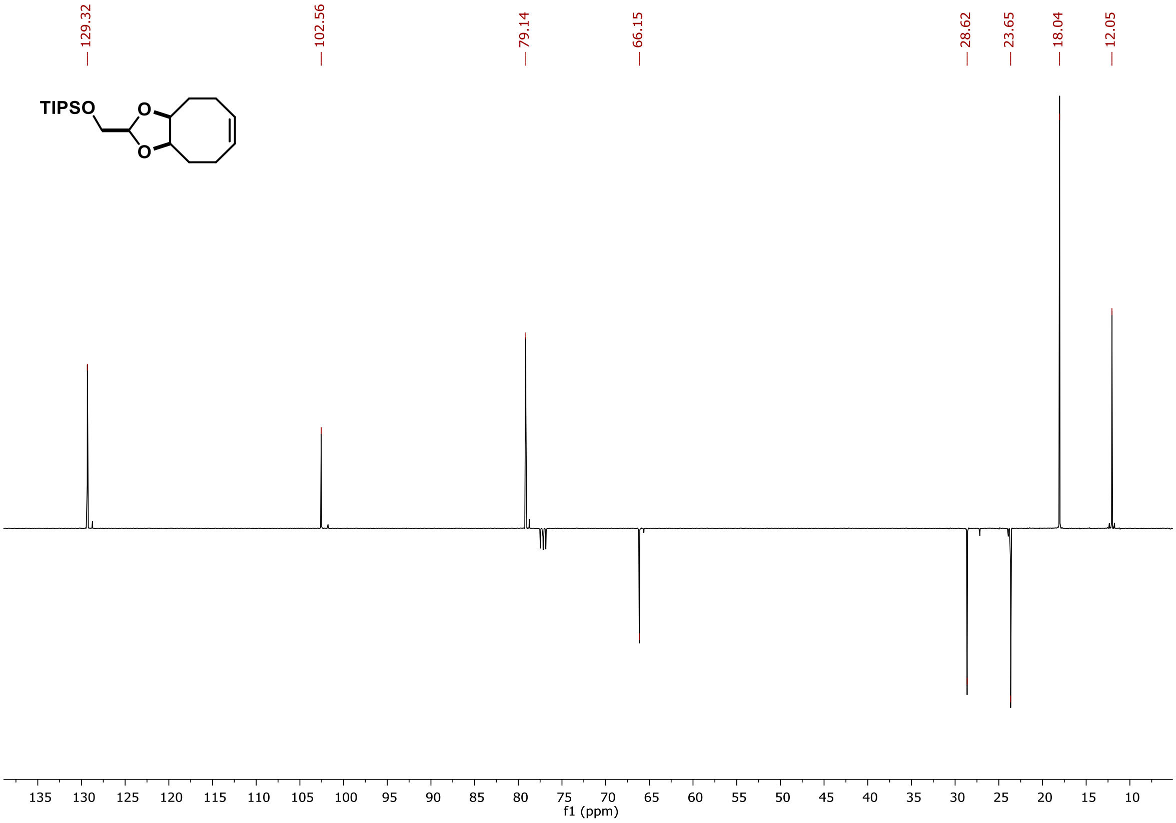

Compound **3**, ^{1}H-NMR

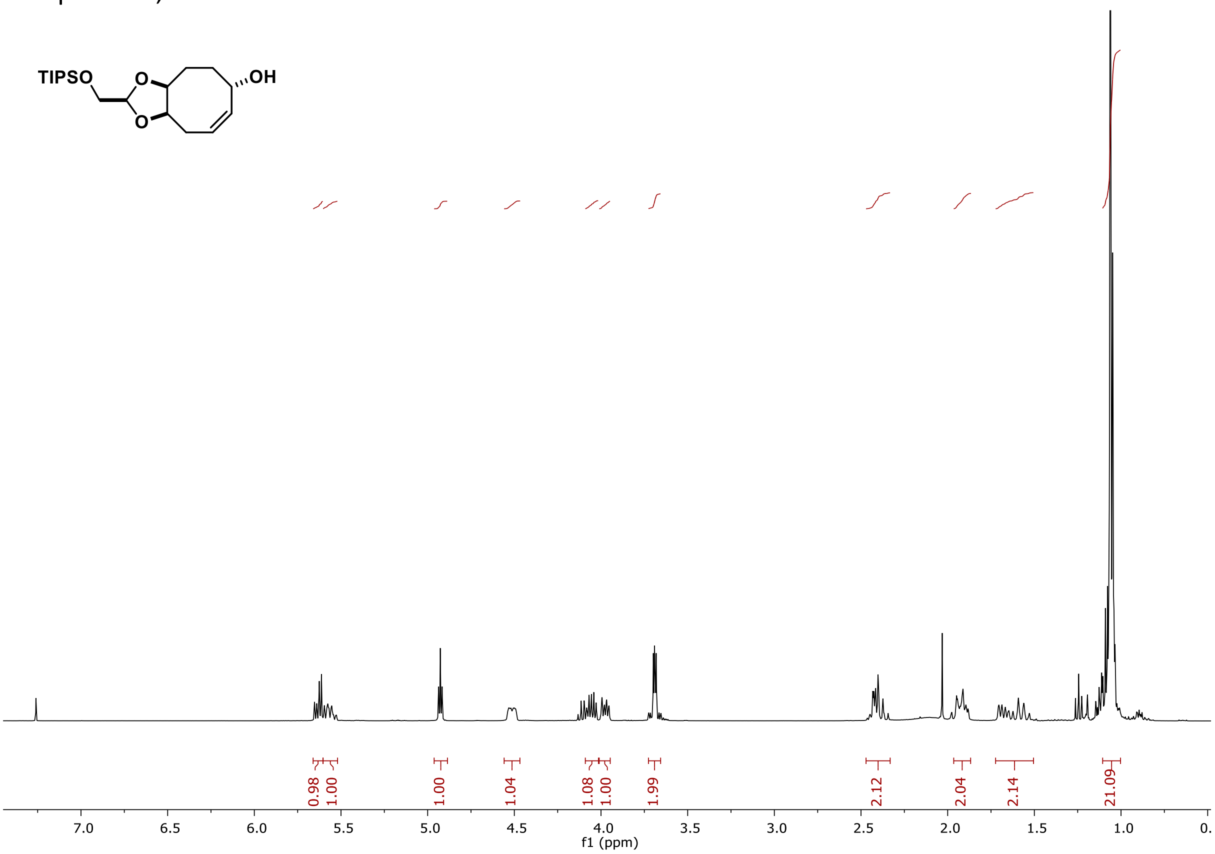

Compound **3**, ^{13}C-NMR (APT)

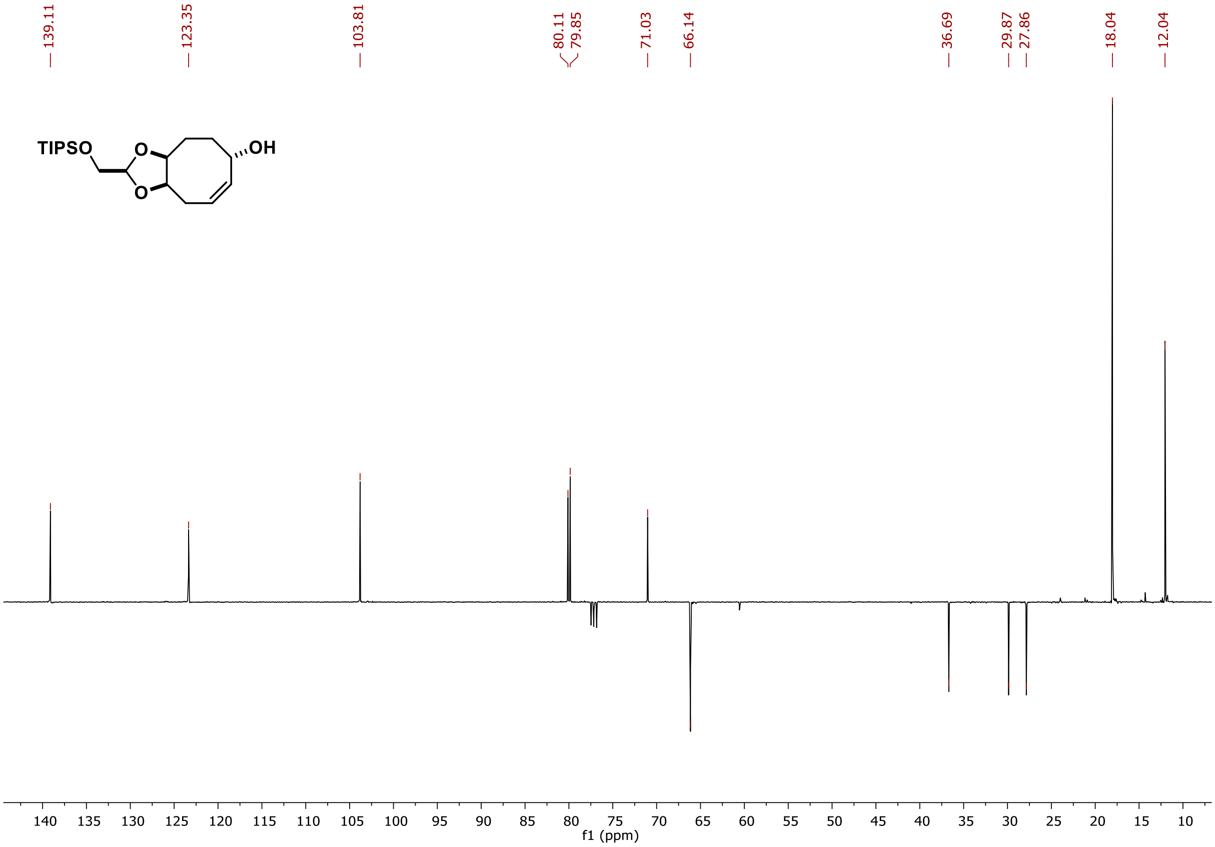

Compound **ax-4**, ^{1}H-NMR

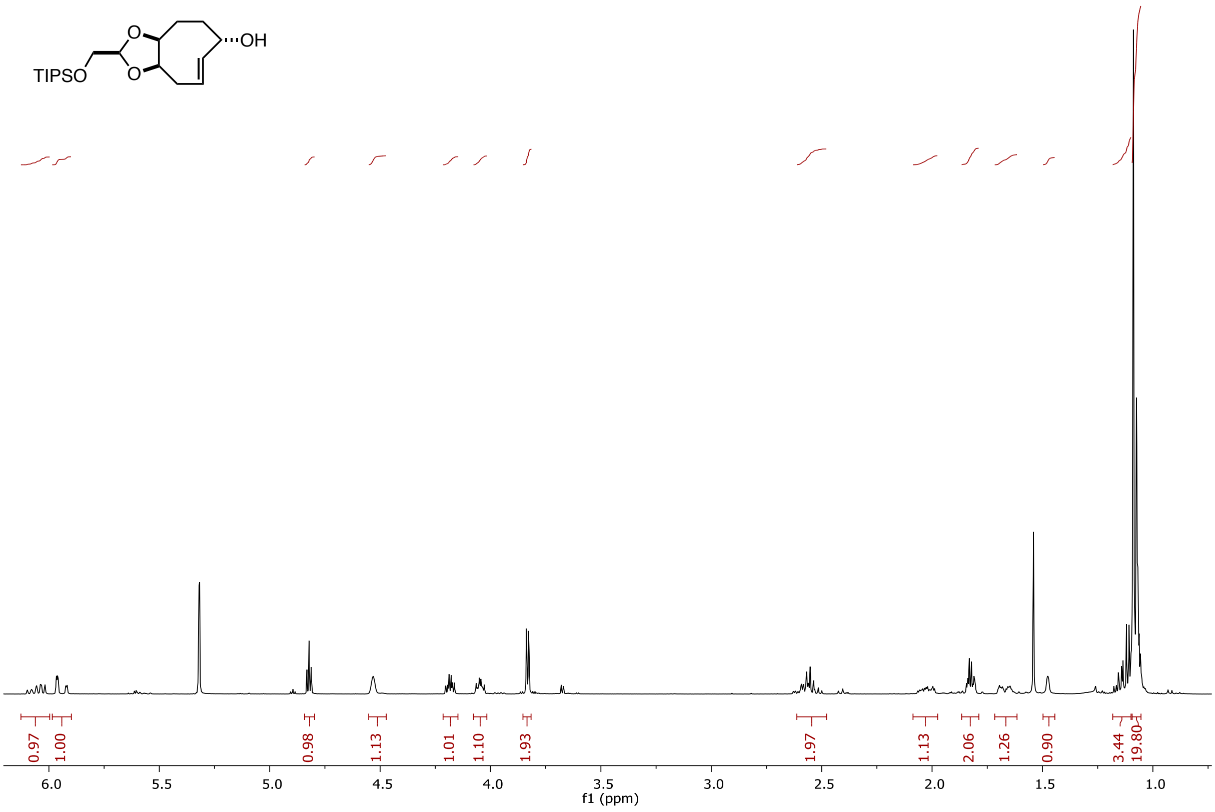

Compound **ax-4**, ^{13}C-NMR (APT)

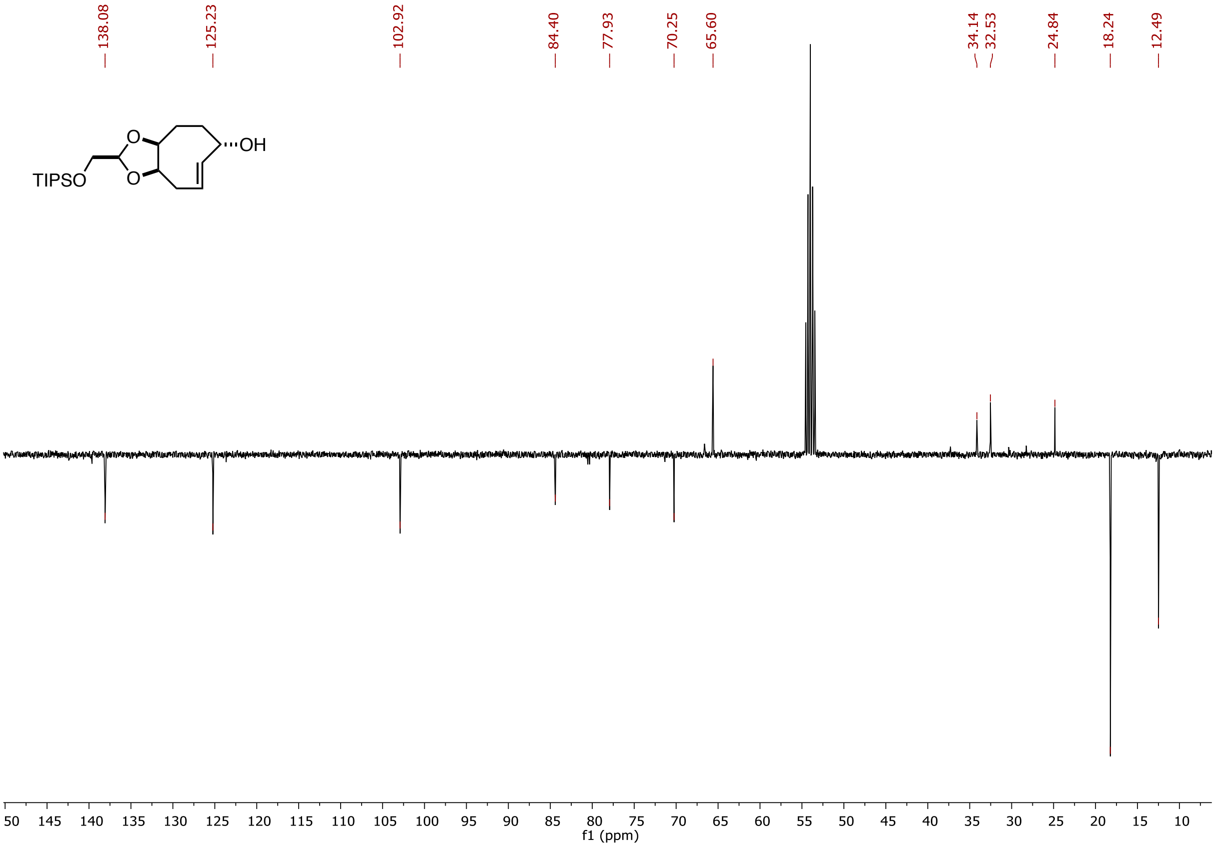

Compound **ax-4**, COSY

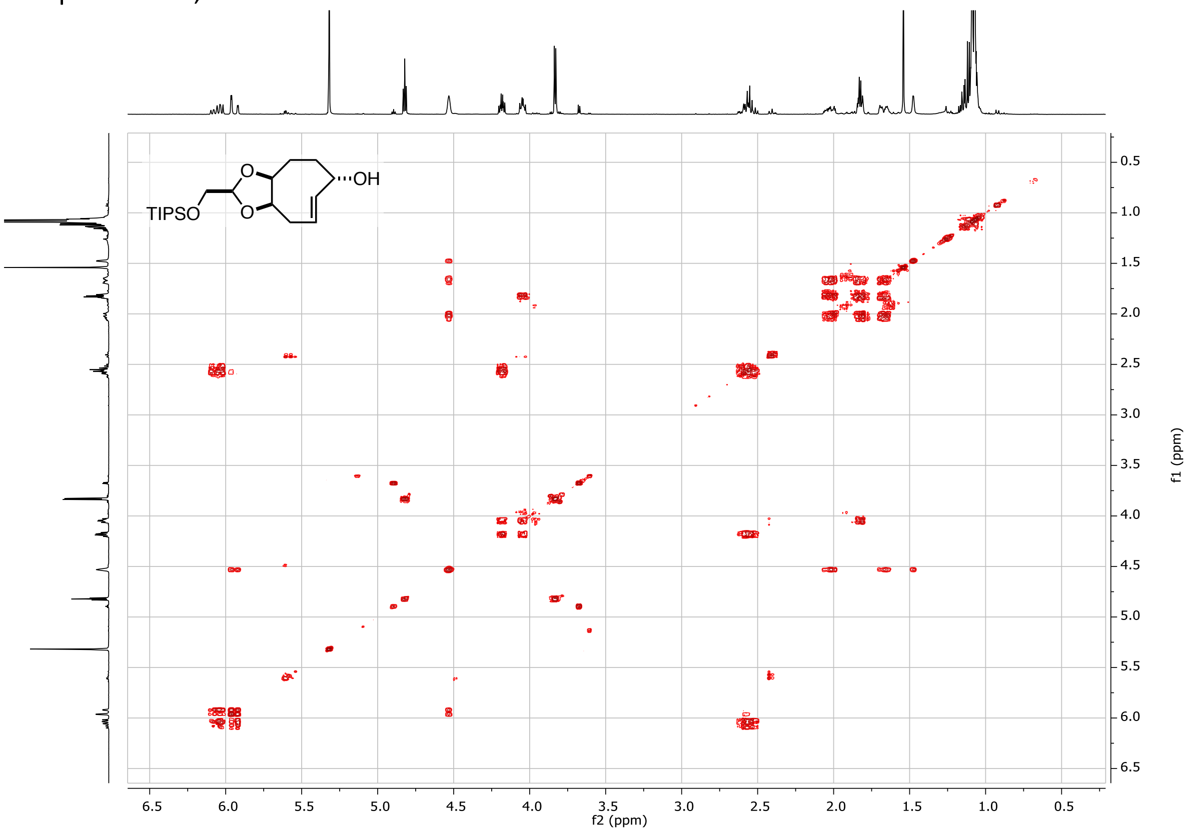

Compound **ax-4**, HSQC

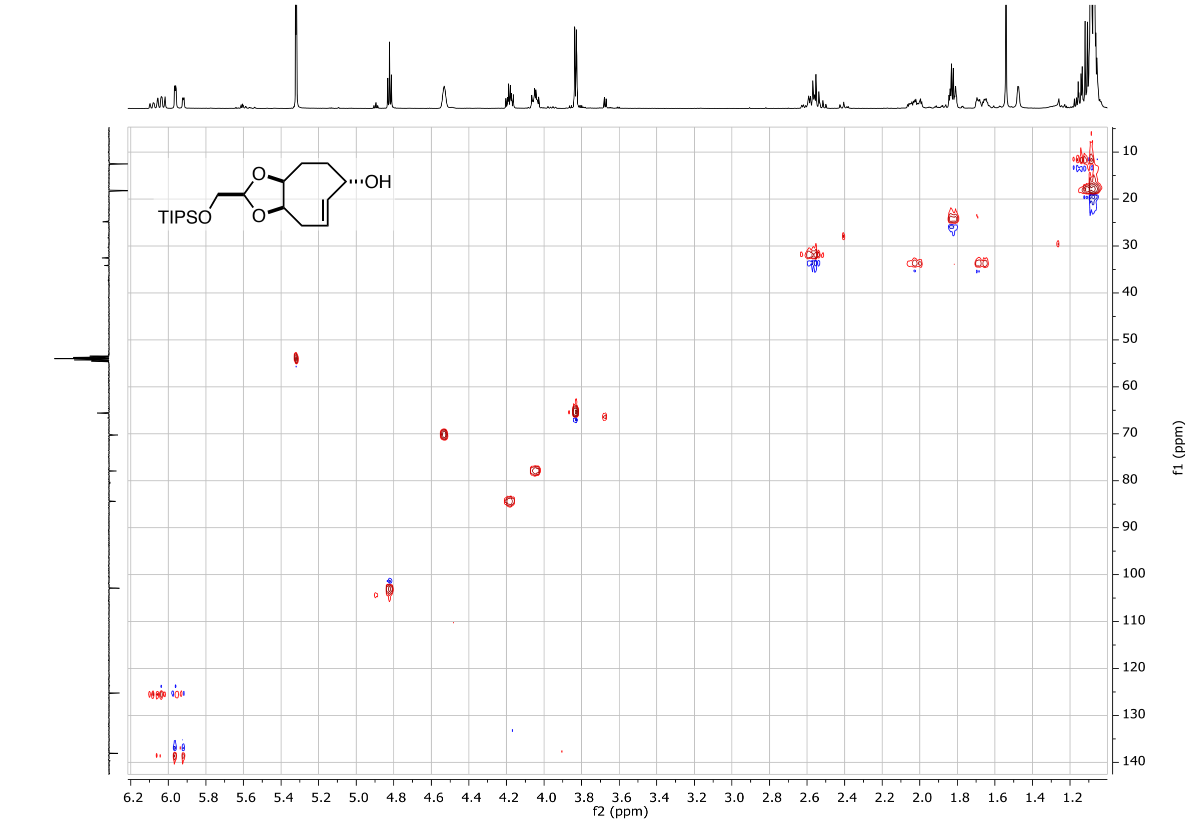

Compound **equ-4**, ^{1}H-NMR

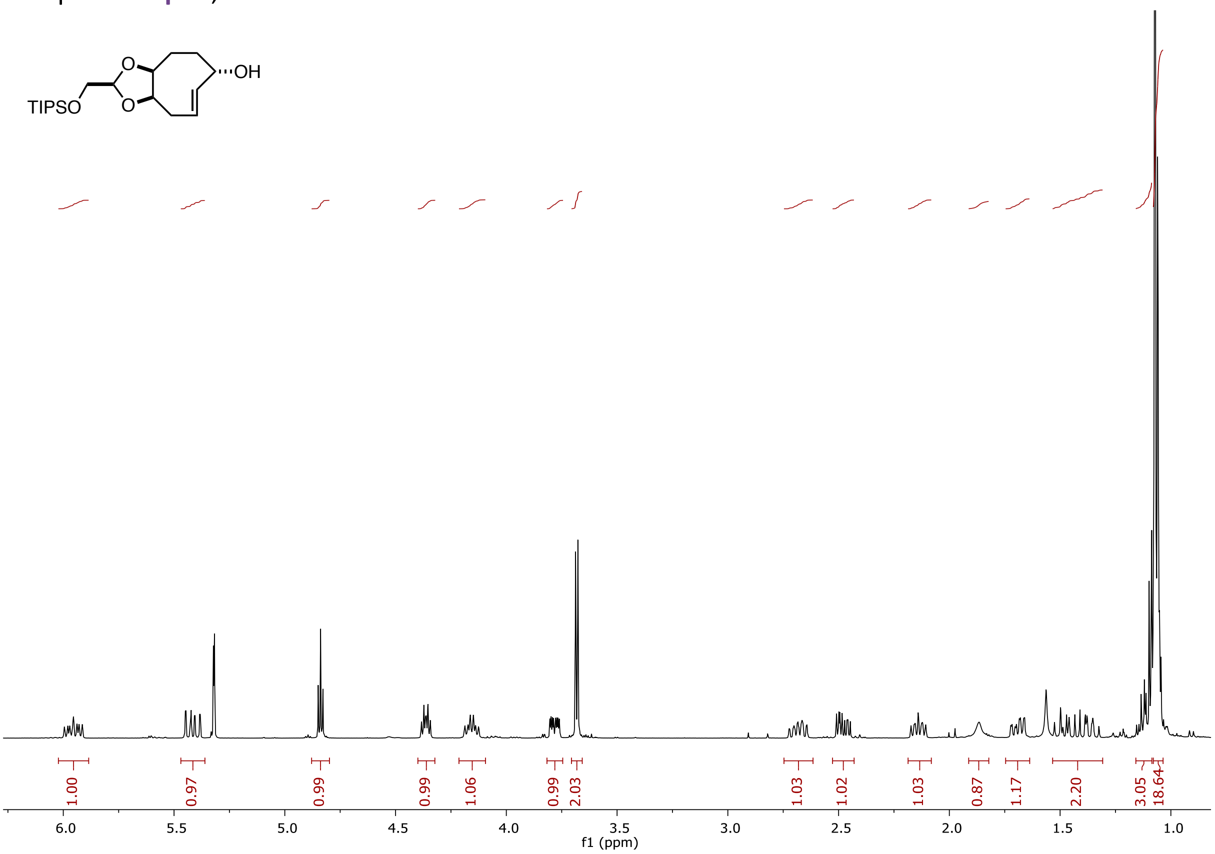

Compound **equ-4**, ^{13}C-NMR (APT)

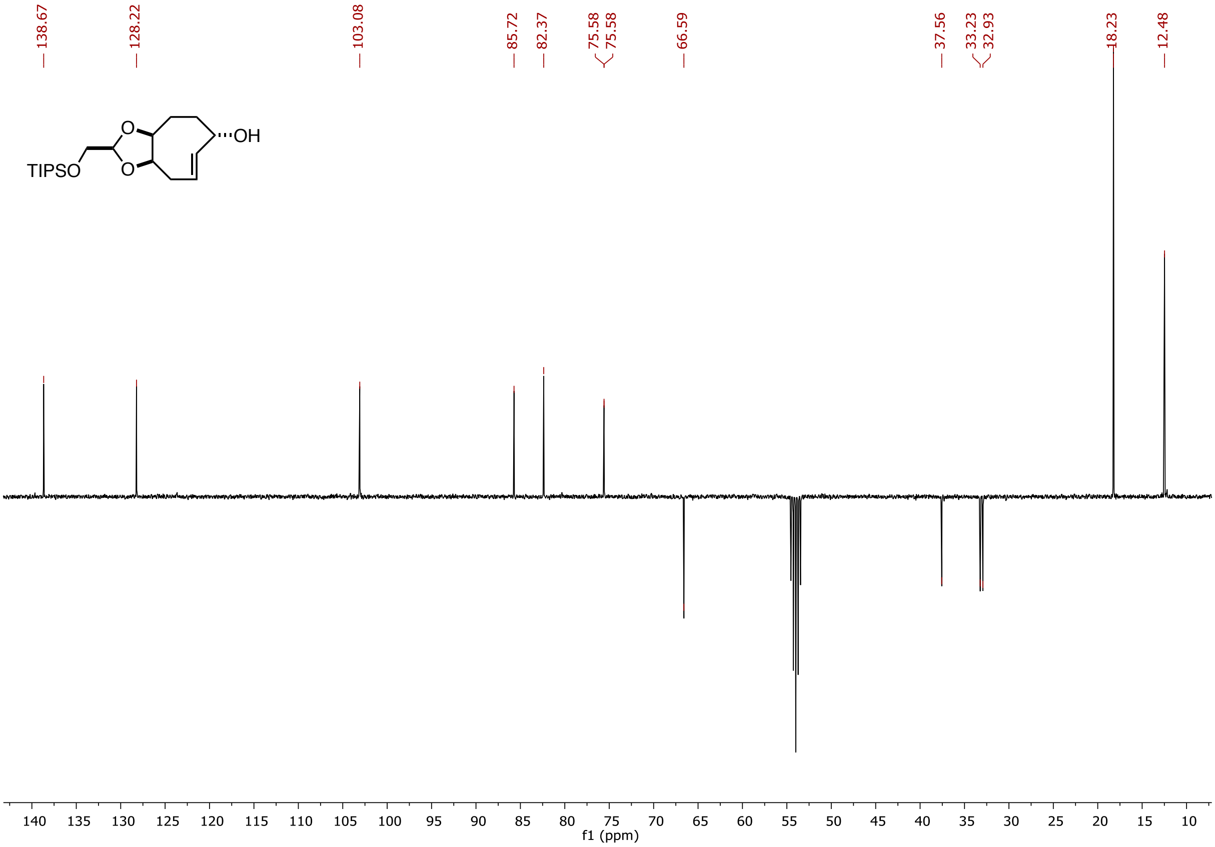

Compound **equ-4**, COSY

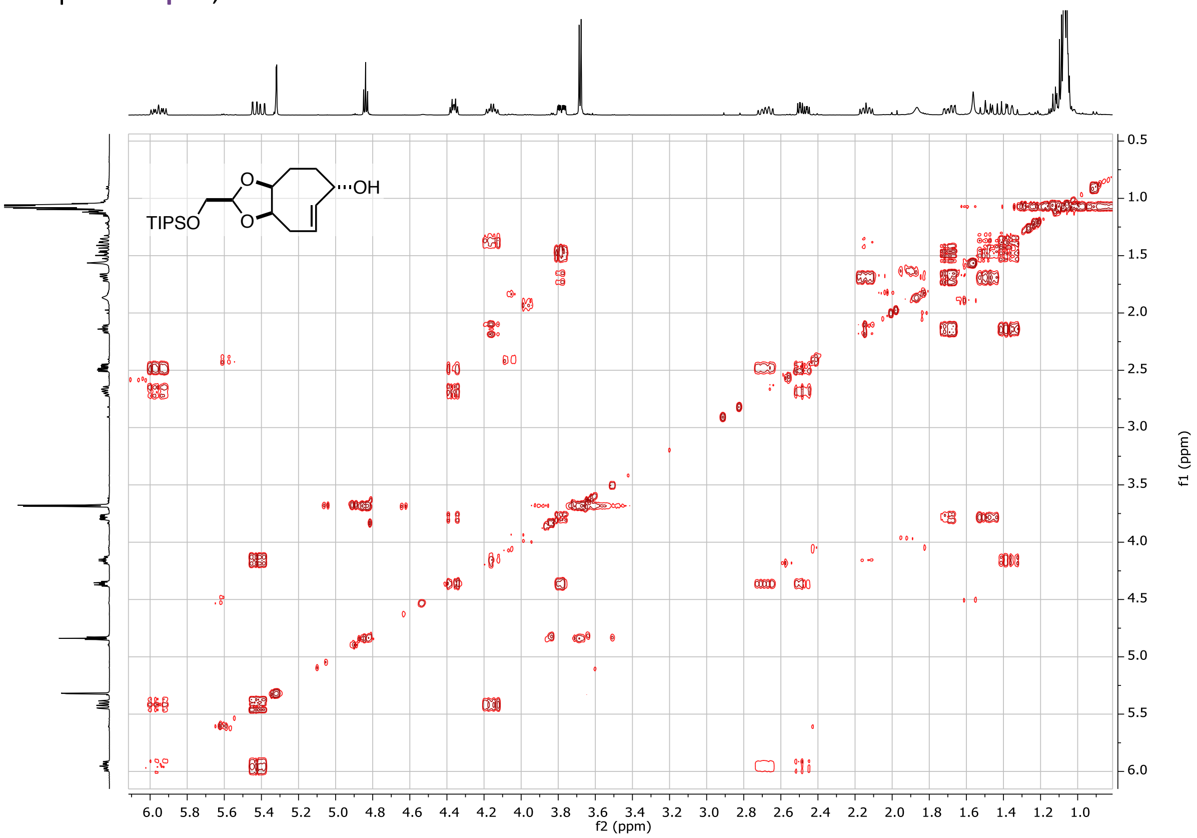

Compound **equ-4**, HSQC

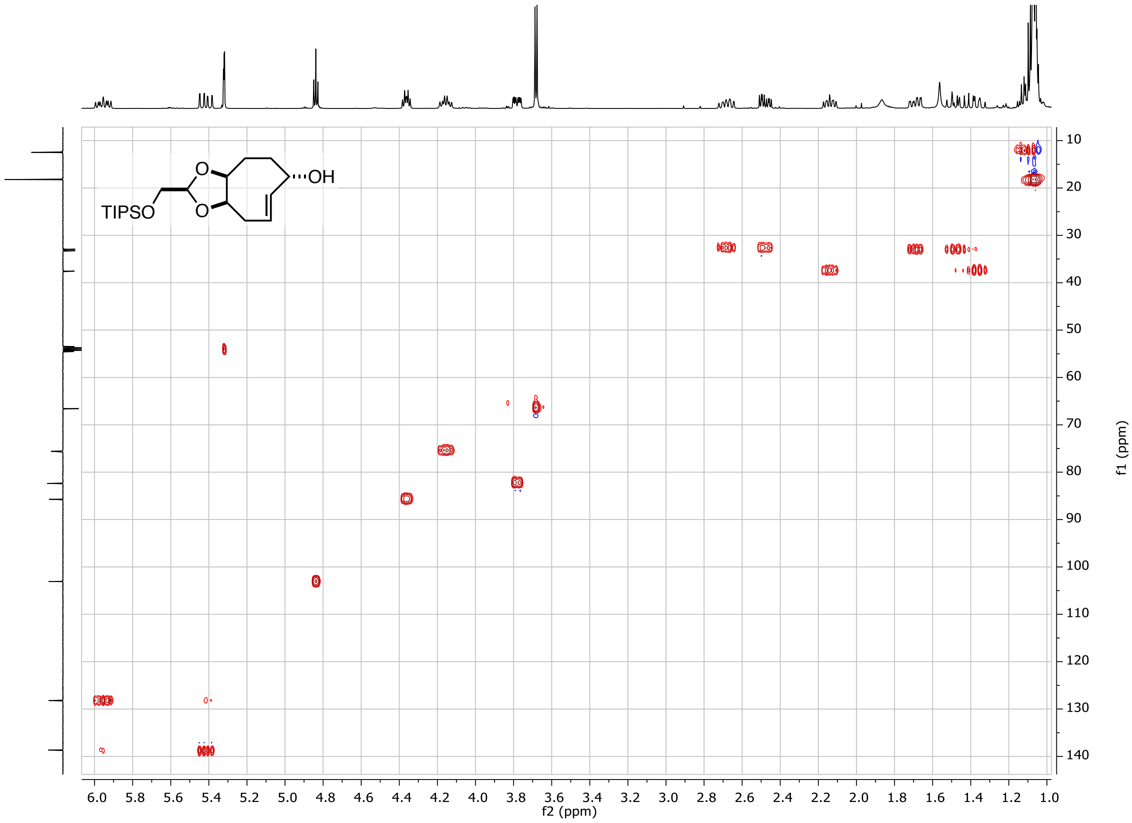

Compound **5**, ^{1}H-NMR

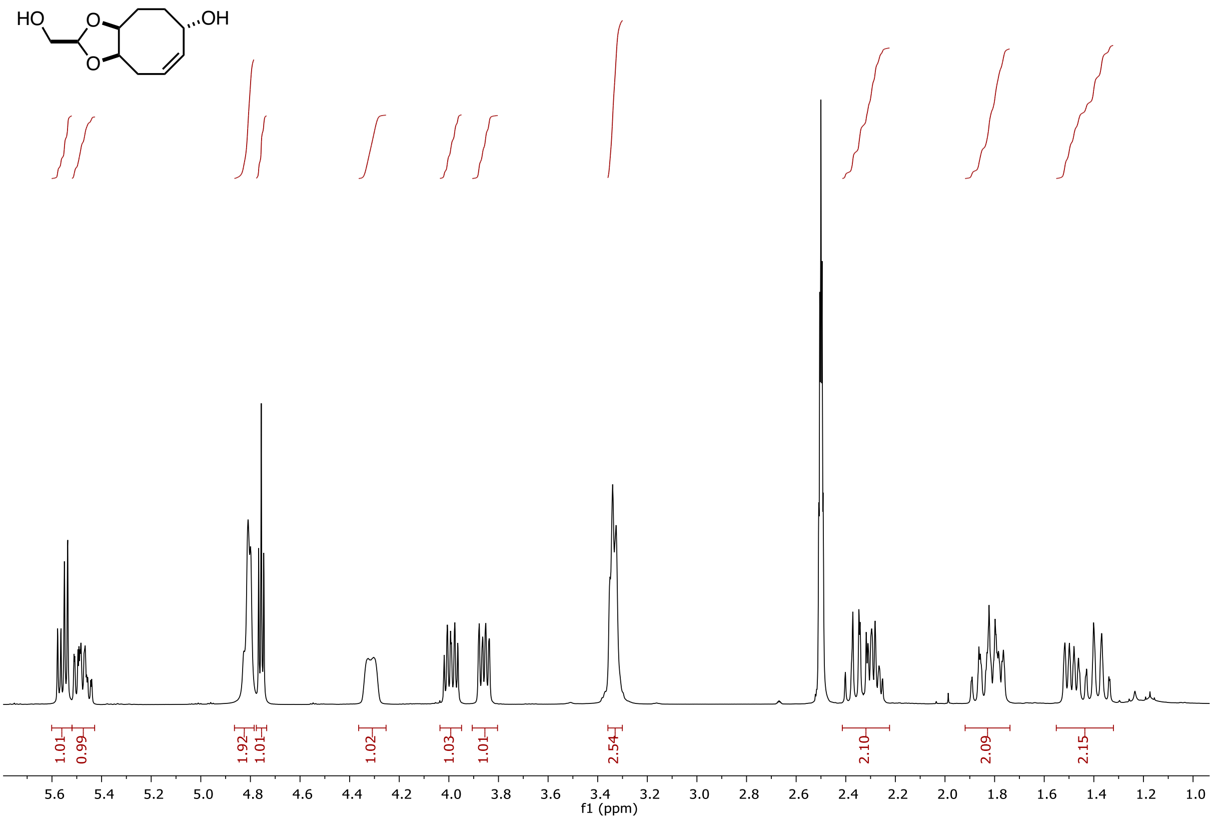

Compound **5**, ^{13}C-NMR

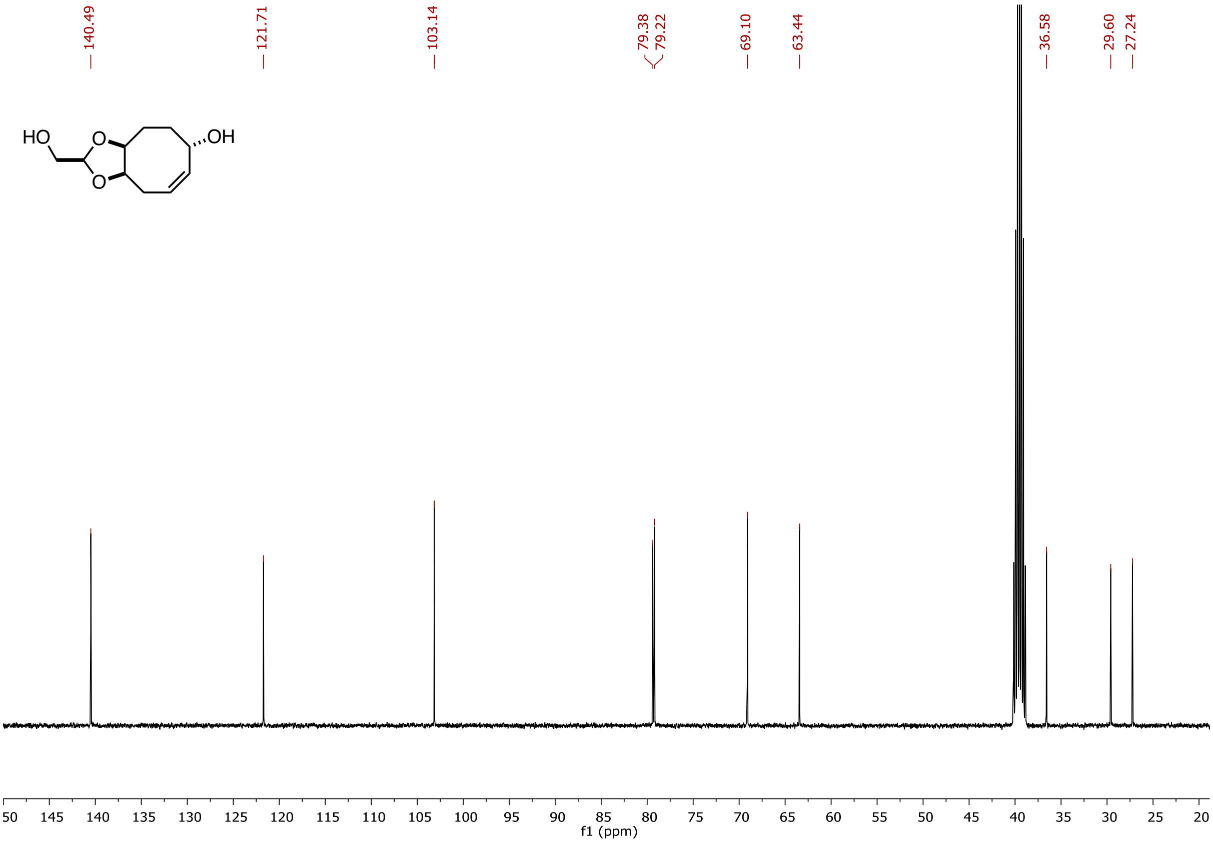

Compound **ax-6**, ^{1}H-NMR

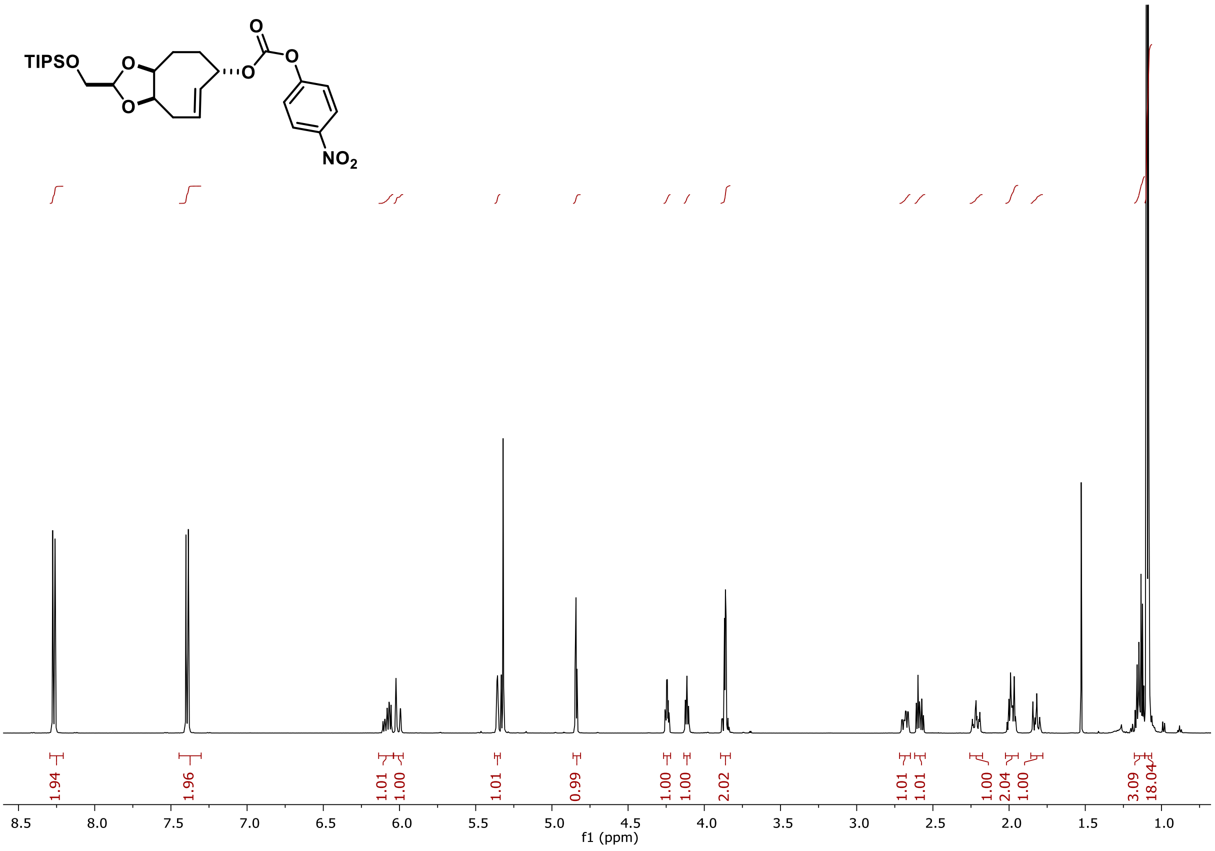

Compound **ax-6**, ^{13}C-NMR

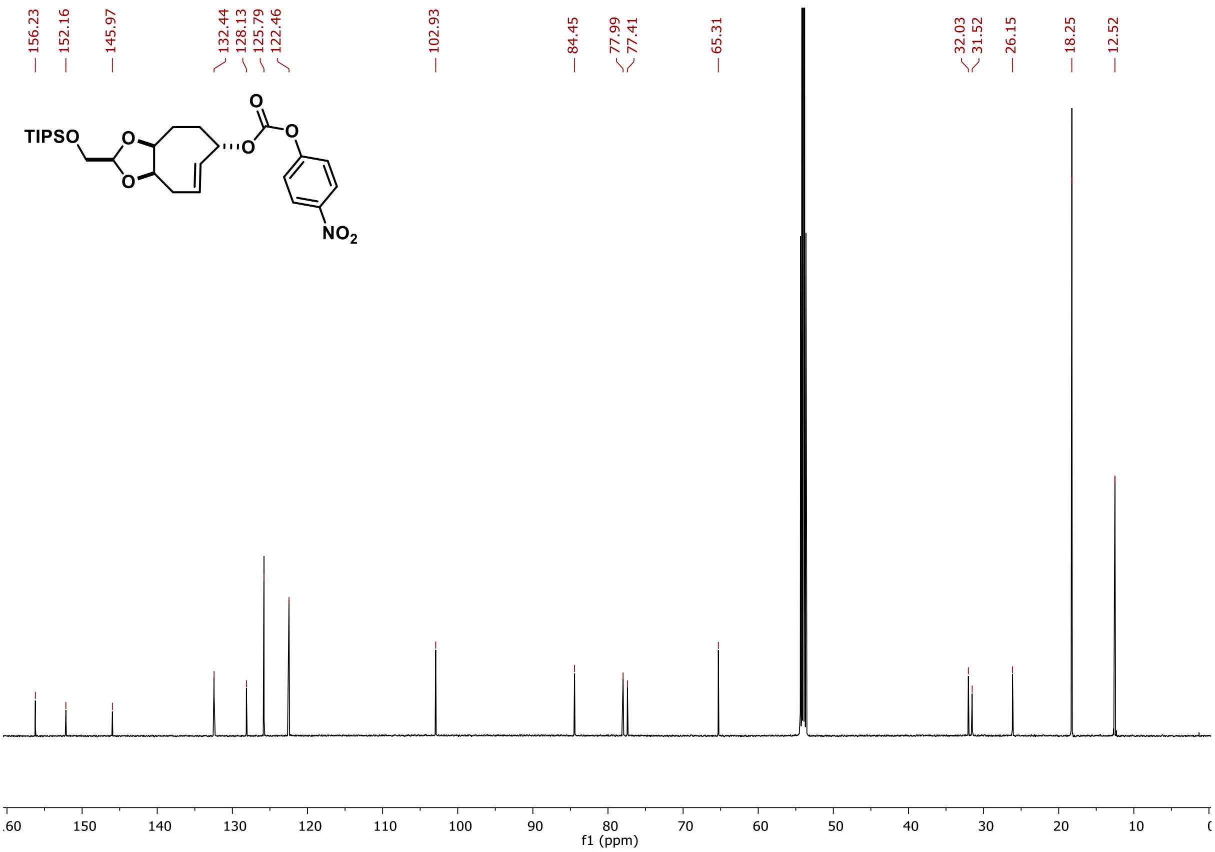

Compound **equ-6**, ^{1}H-NMR

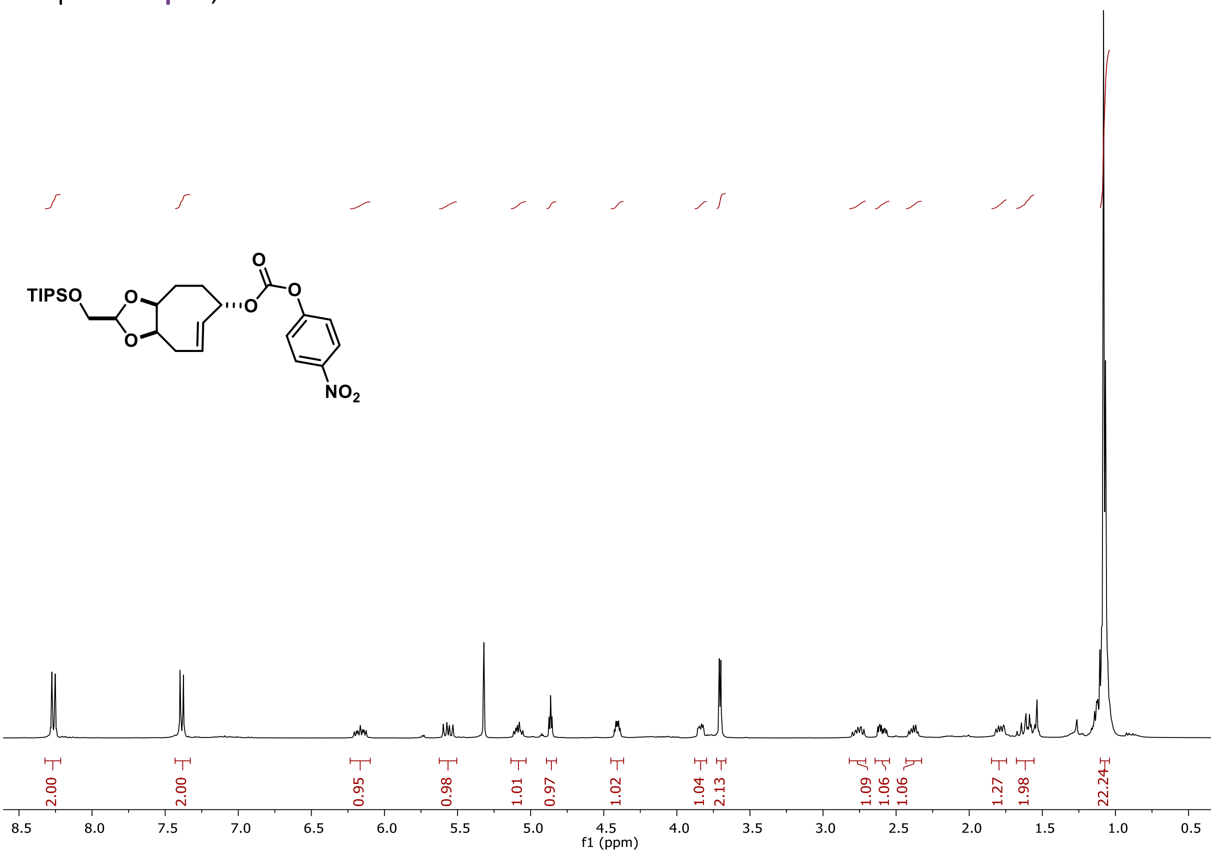

Compound **equ-6**, ^{13}C-NMR (APT)

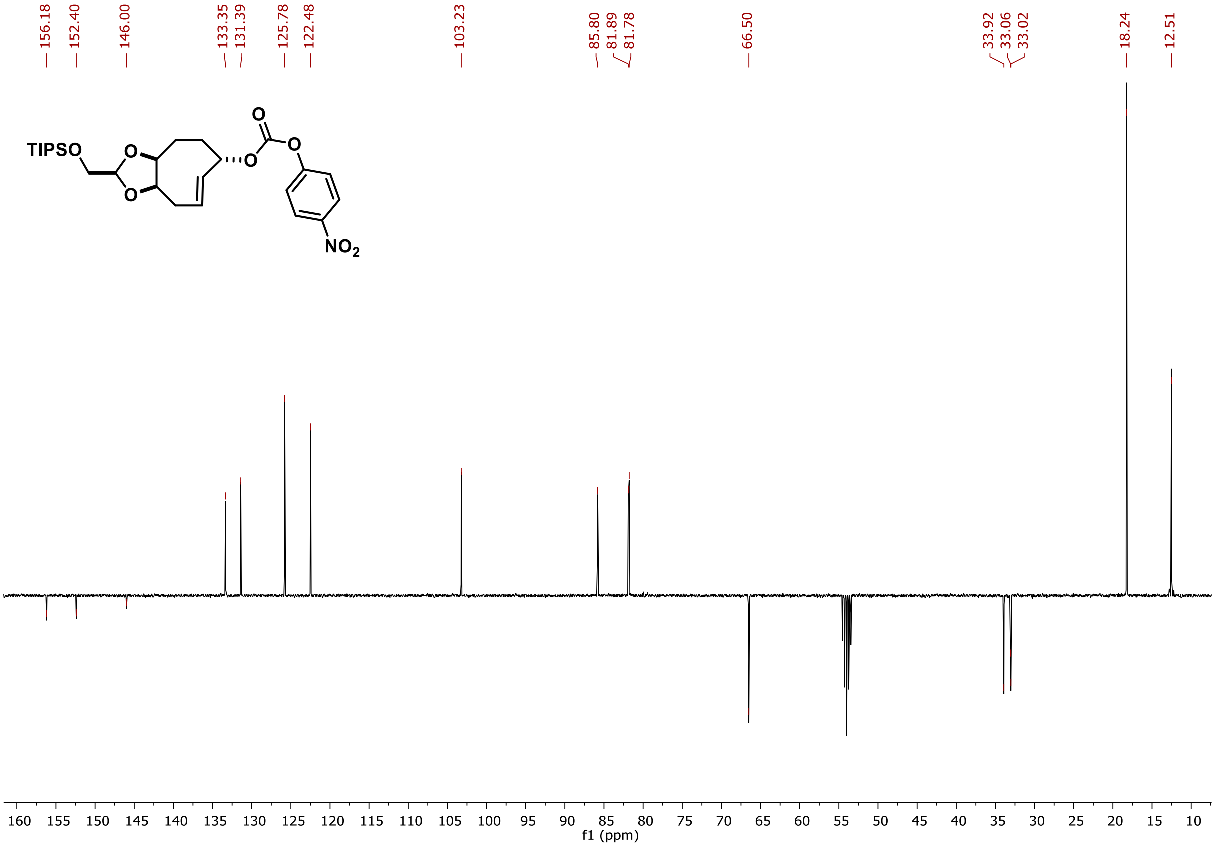

Compound **ax-S1**, ^{1}H-NMR

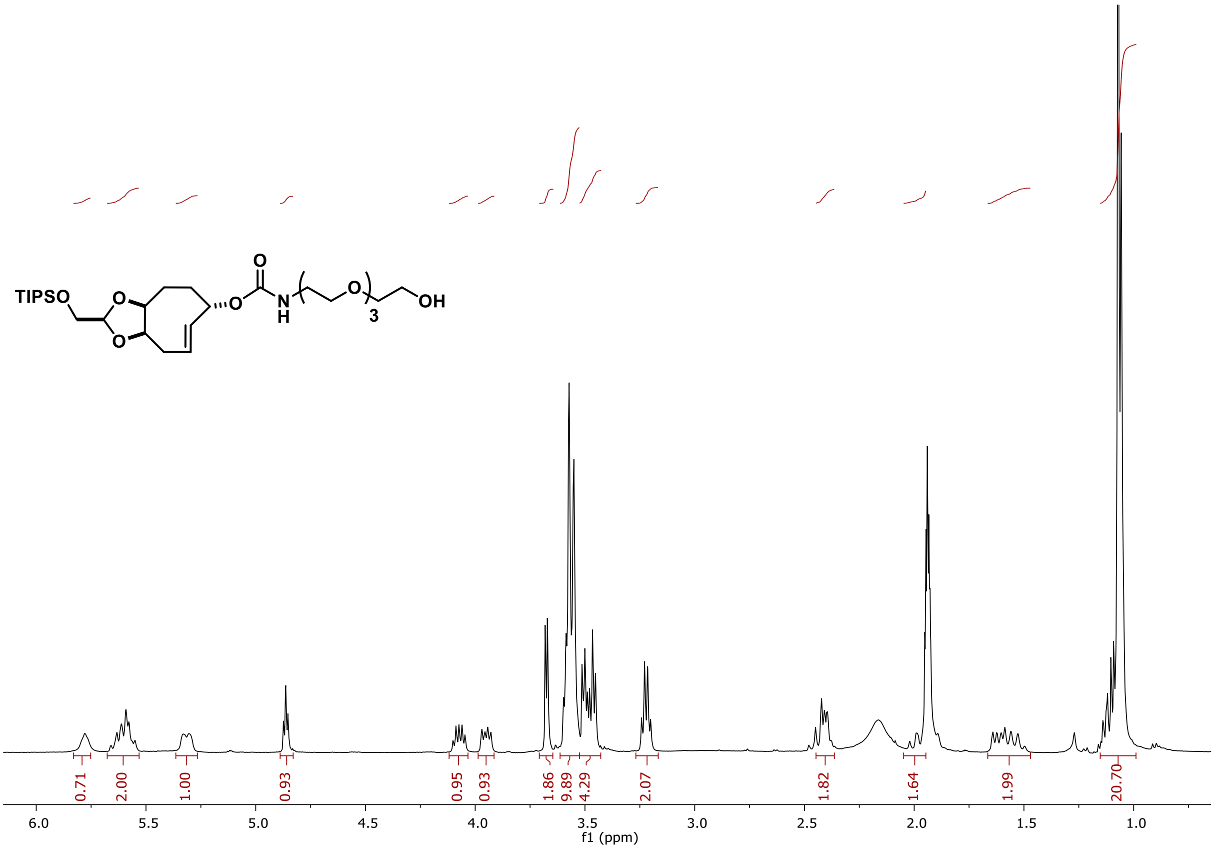

Compound **ax-S1**, ^{13}C-NMR

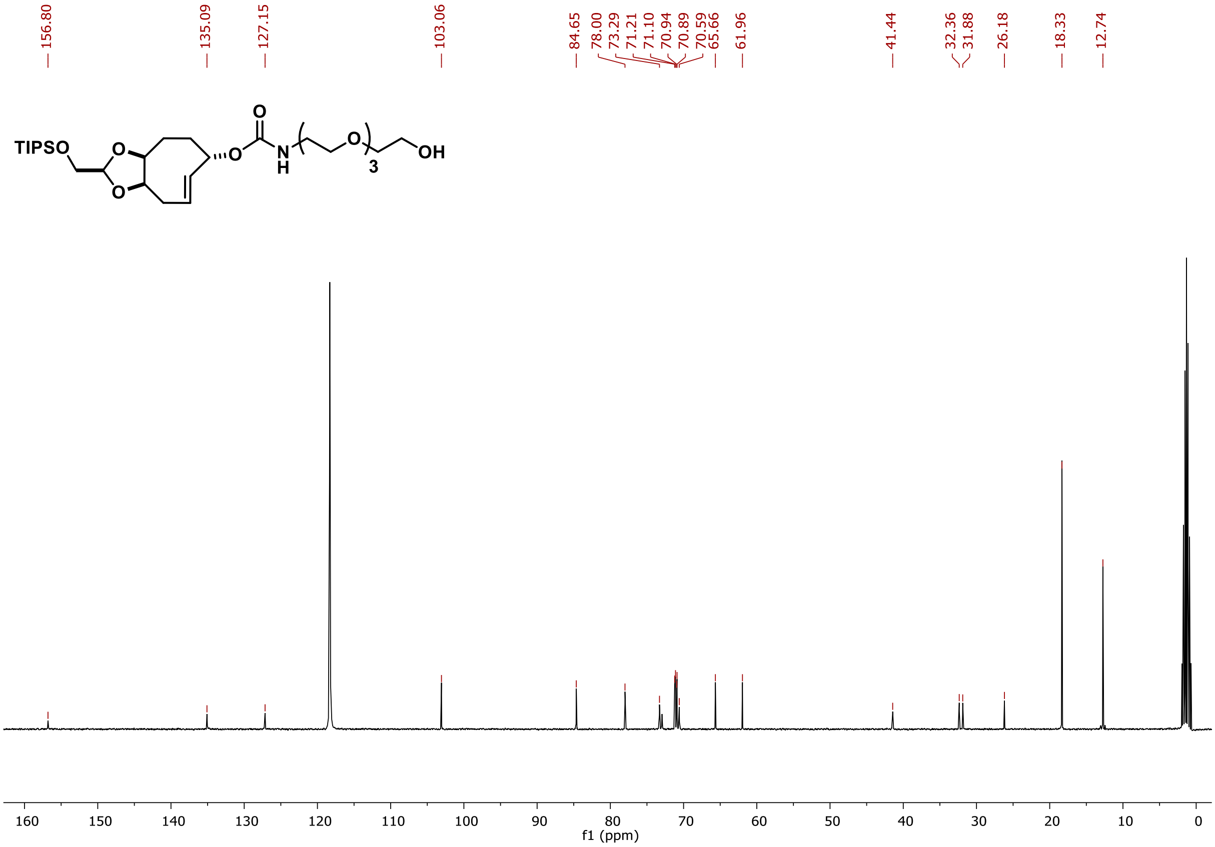

Compound **equ-S1**, ^{1}H-NMR

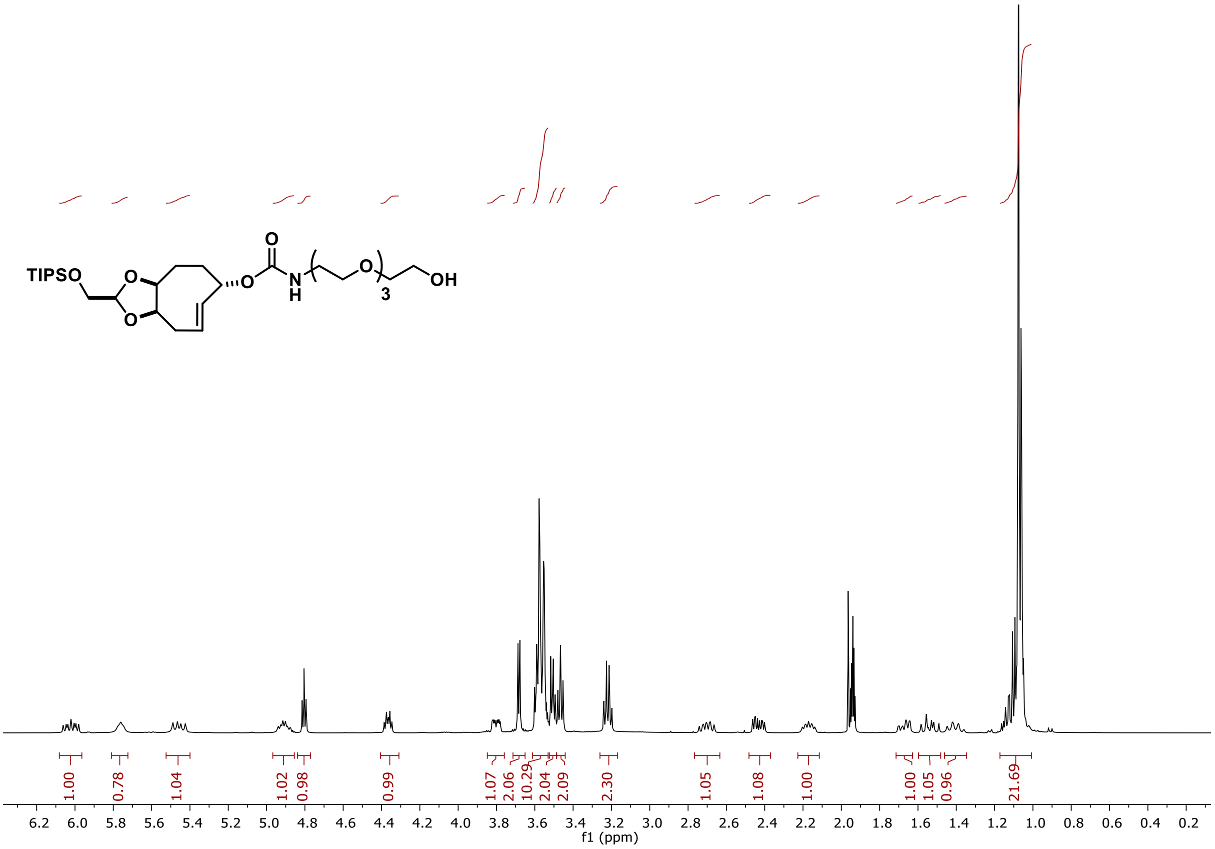

Compound **equ-S1**, ^{13}C-NMR

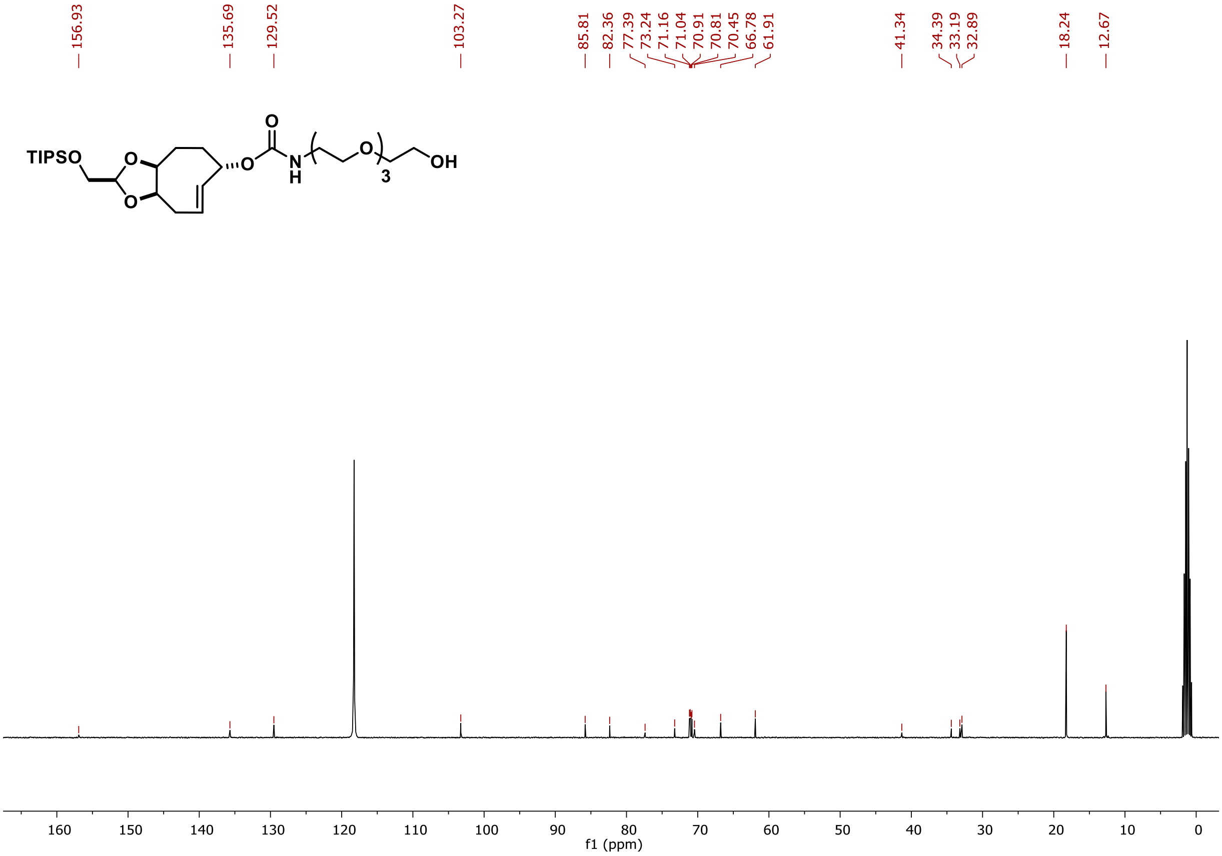

Compound **ax-7**, ^{1}H-NMR

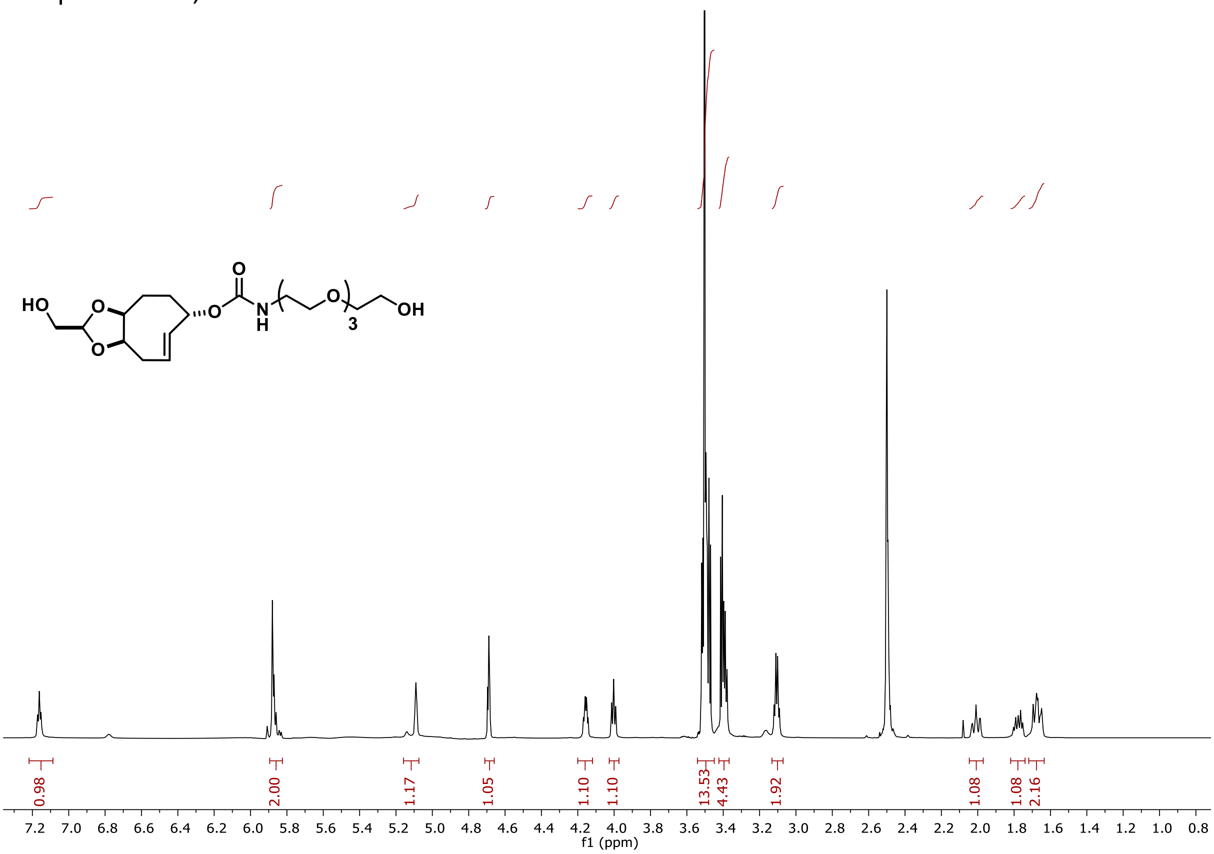

Compound **ax-7**, ^{13}C-NMR

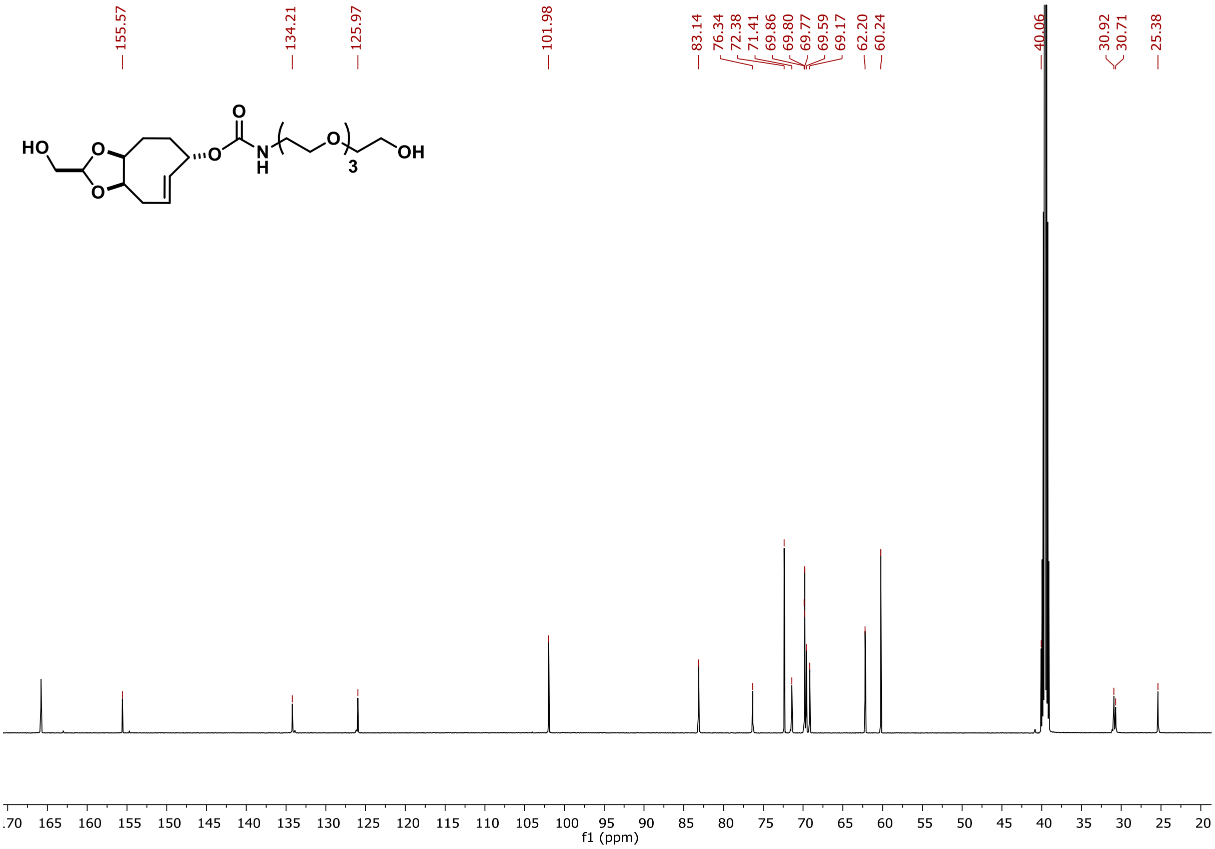

Compound **equ-7**, ^{1}H-NMR

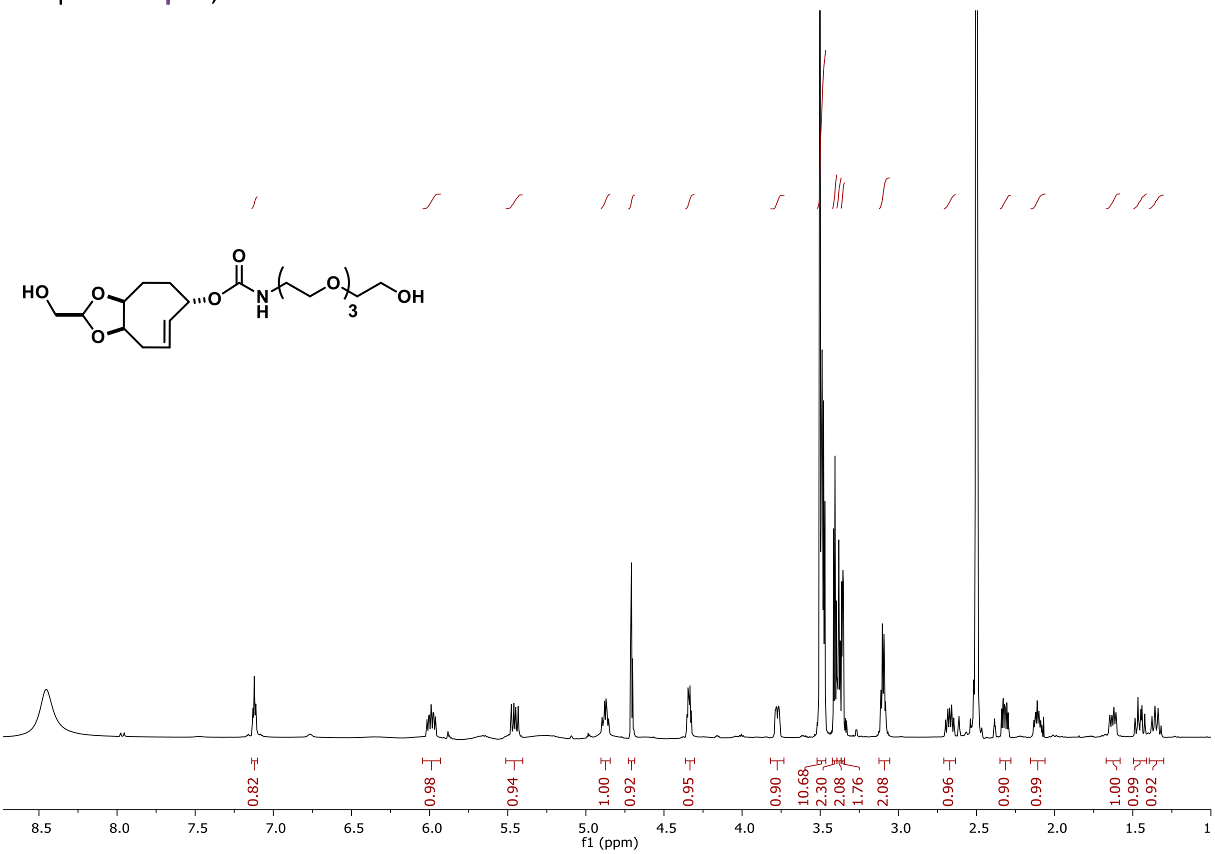

Compound **equ-7**, ^{13}C-NMR

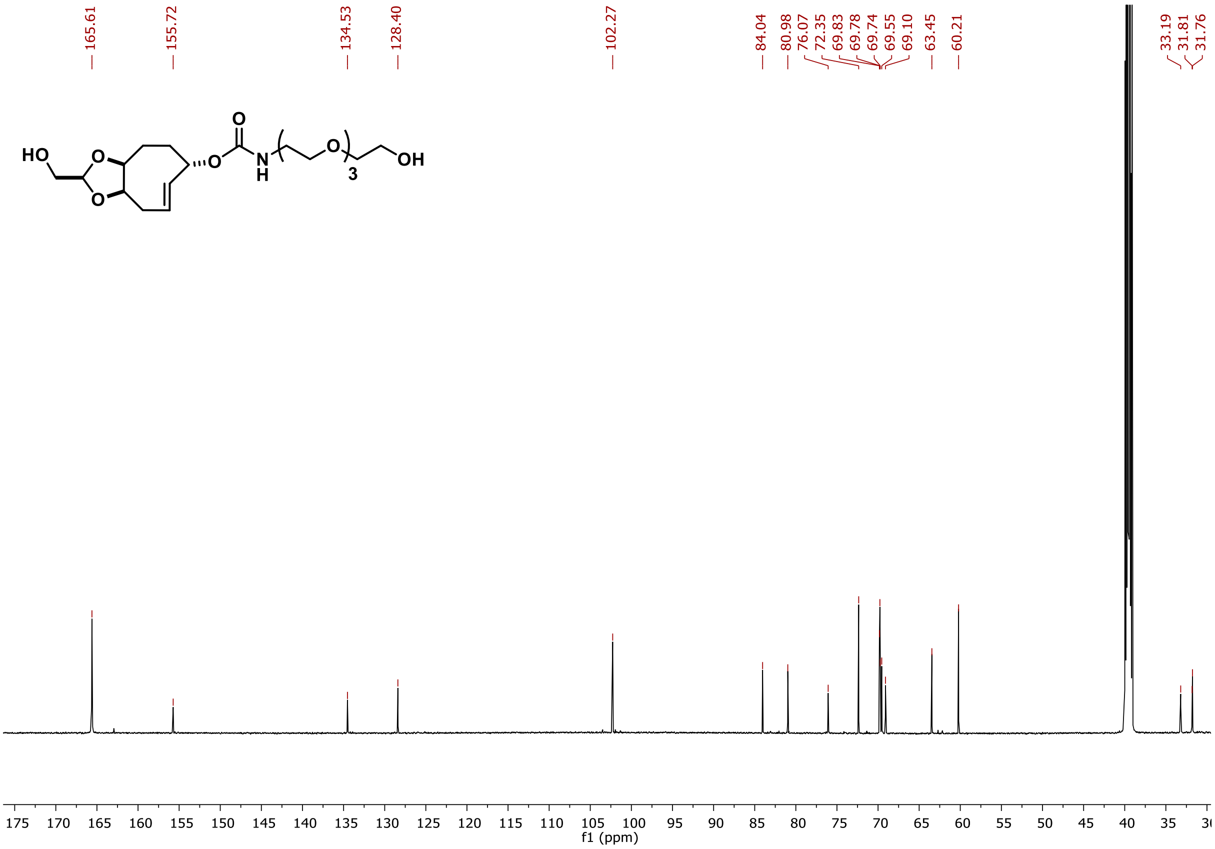

Compound **S2**, ^{1}H-NMR

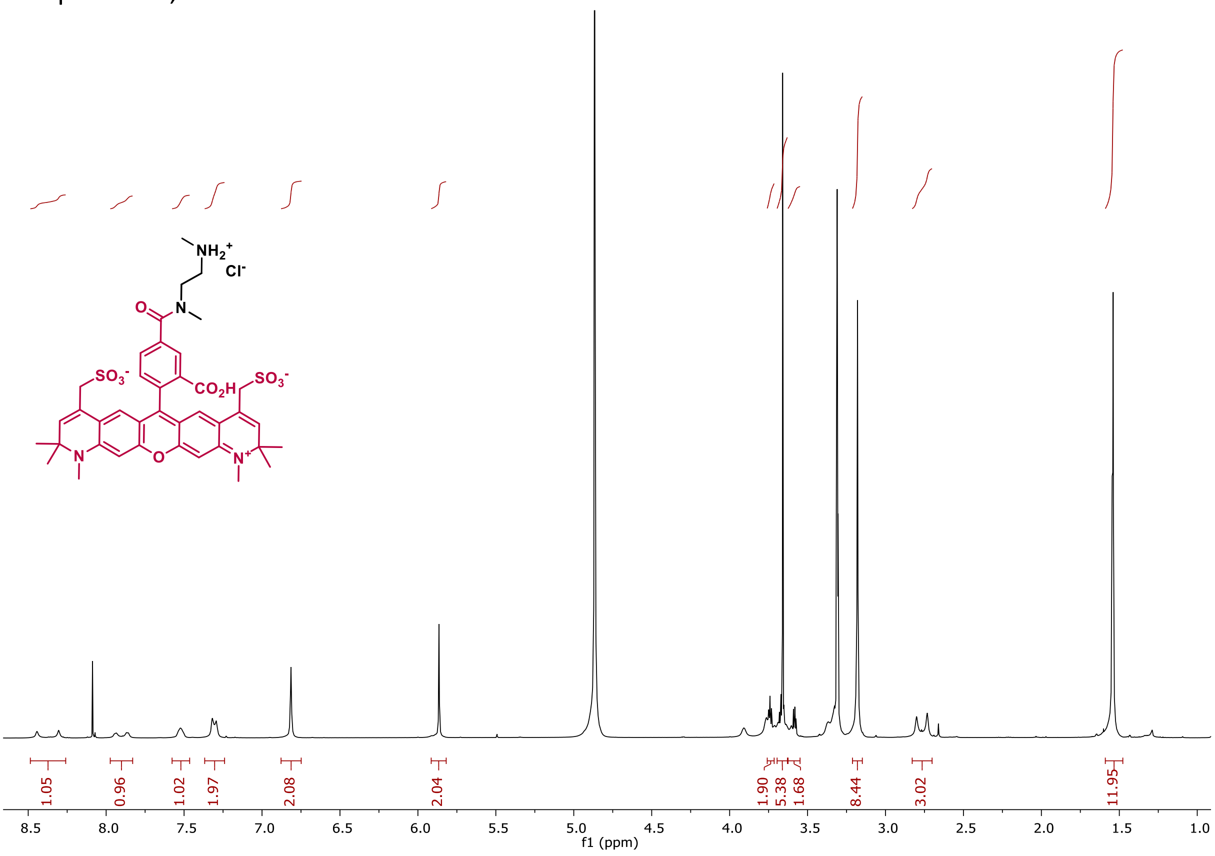

Compound **S2**, ^{13}C-NMR

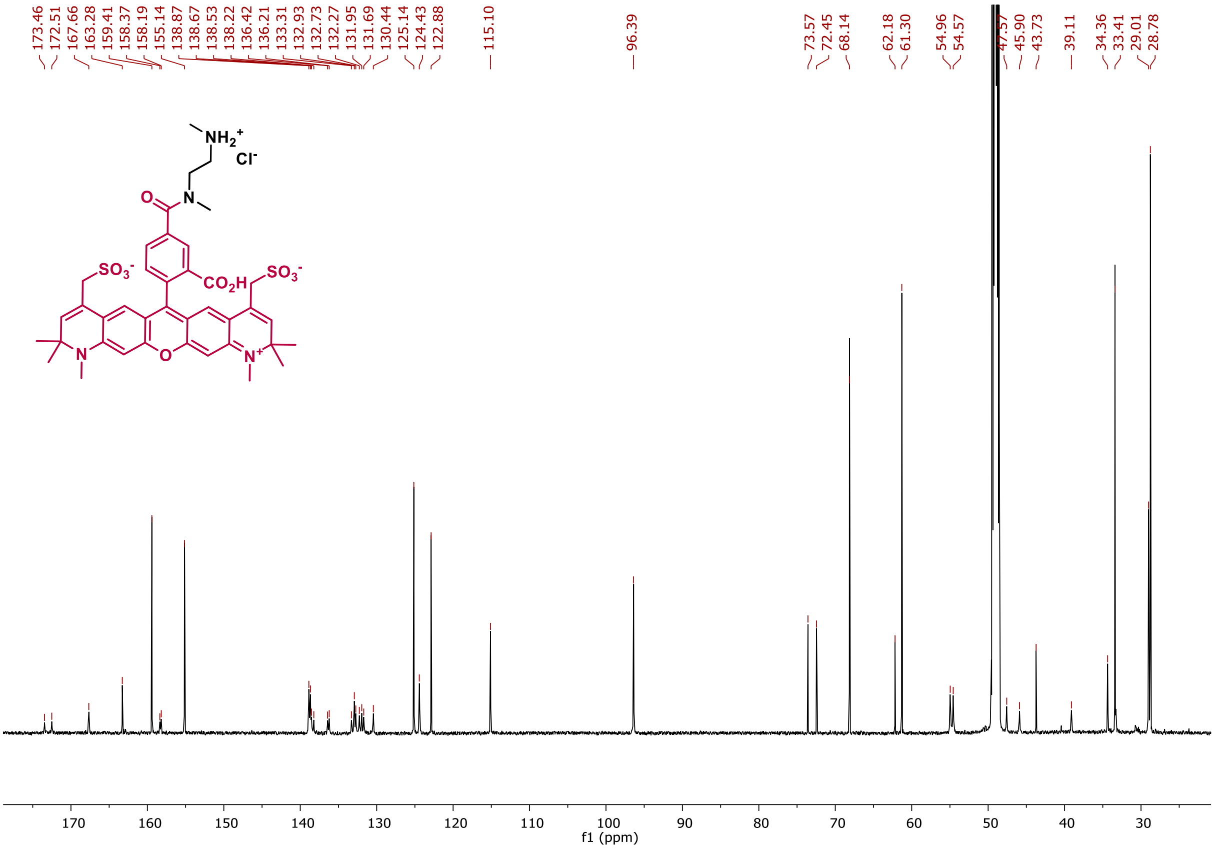

Compound **ax**-**S3**, ^{1}H-NMR

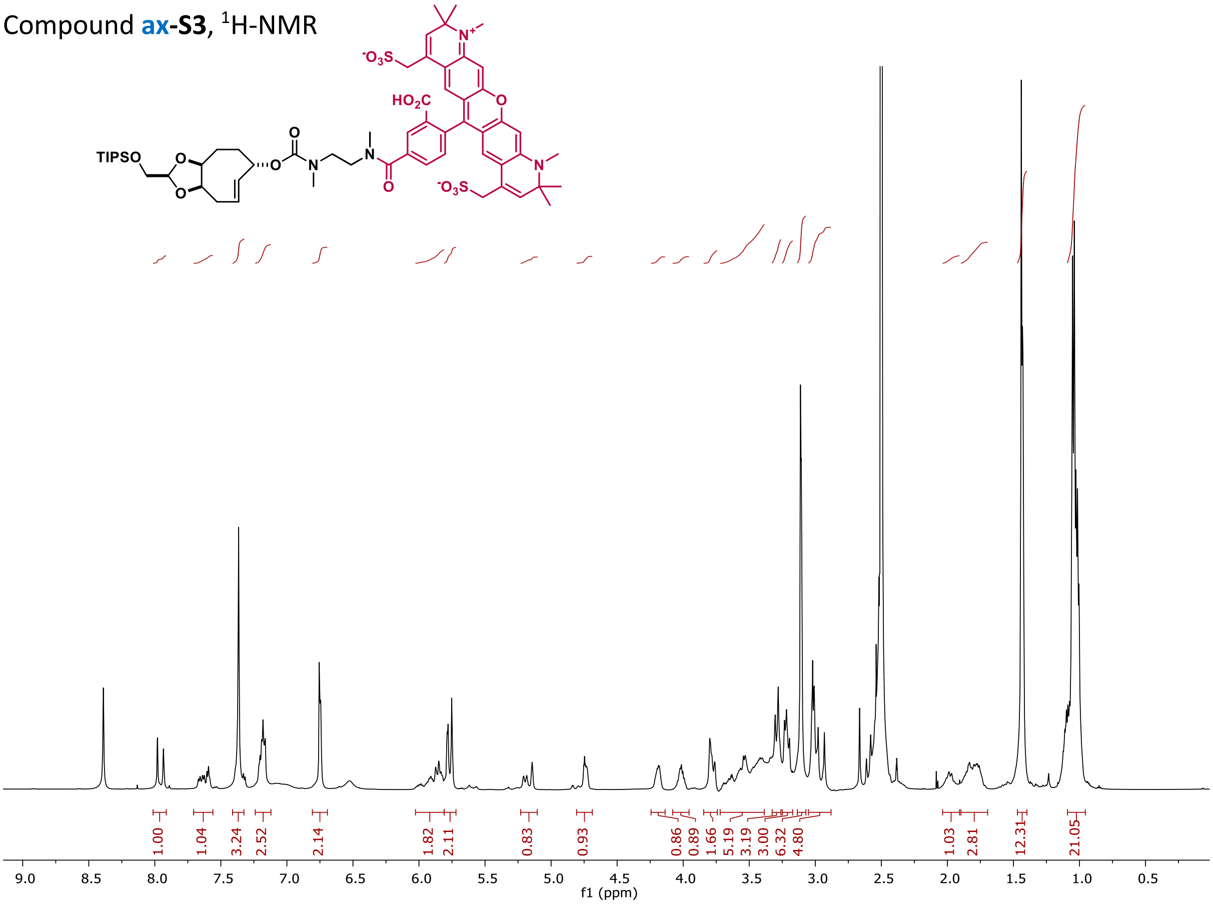

Compound **ax**-**S3**, HPLC (buffered conditions, ammonium formate (pH 8.4)/acetonitrile gradient elution)

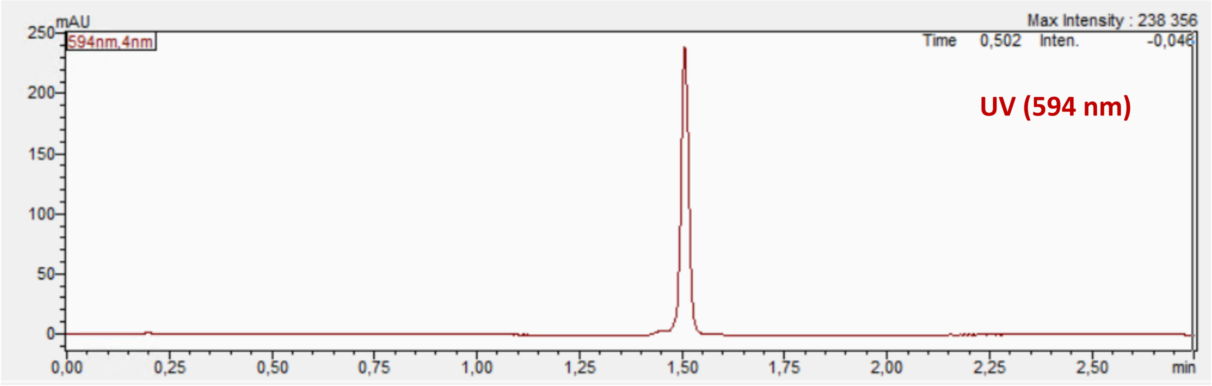

Compound **ax-8**, ^{1}H-NMR

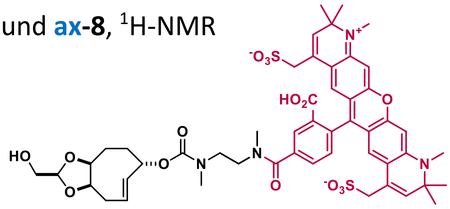

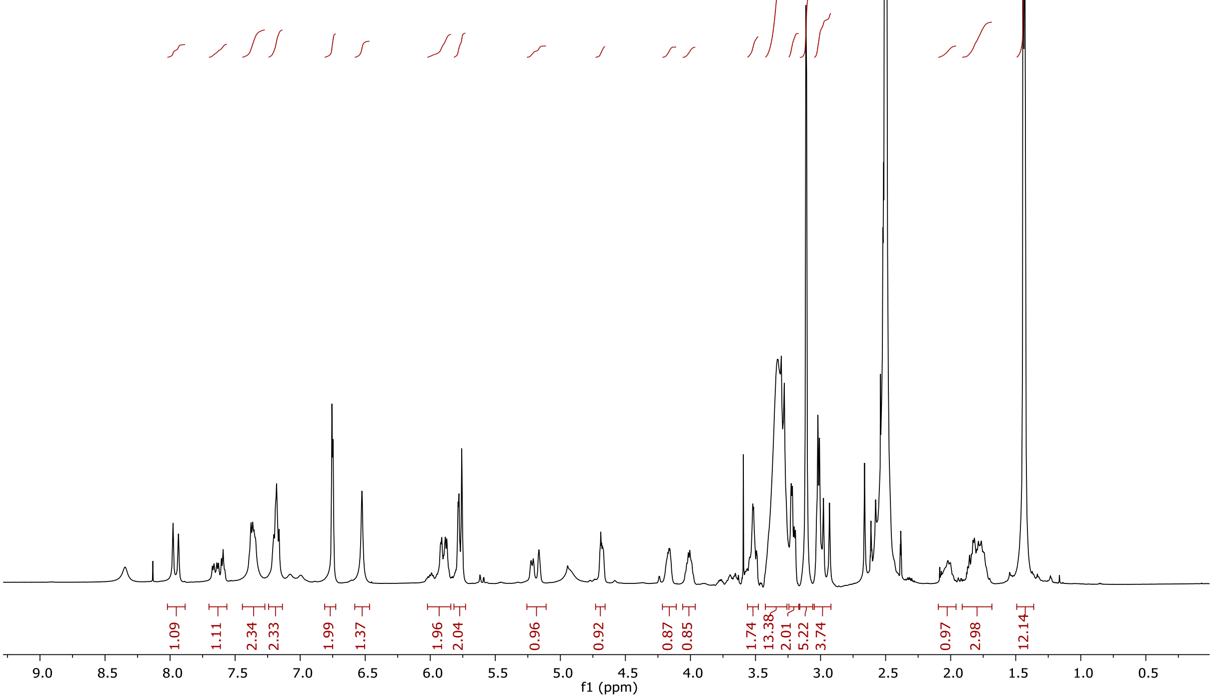

Compound **ax-8**, HPLC (acidic conditions, water/MeCN + 0.1% formic acid, gradient elution)

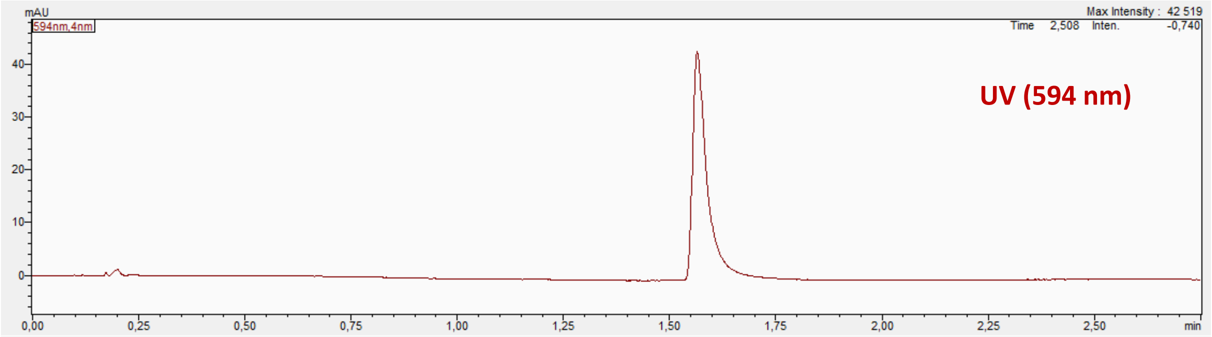

Compound **S5**, ^{1}H-NMR

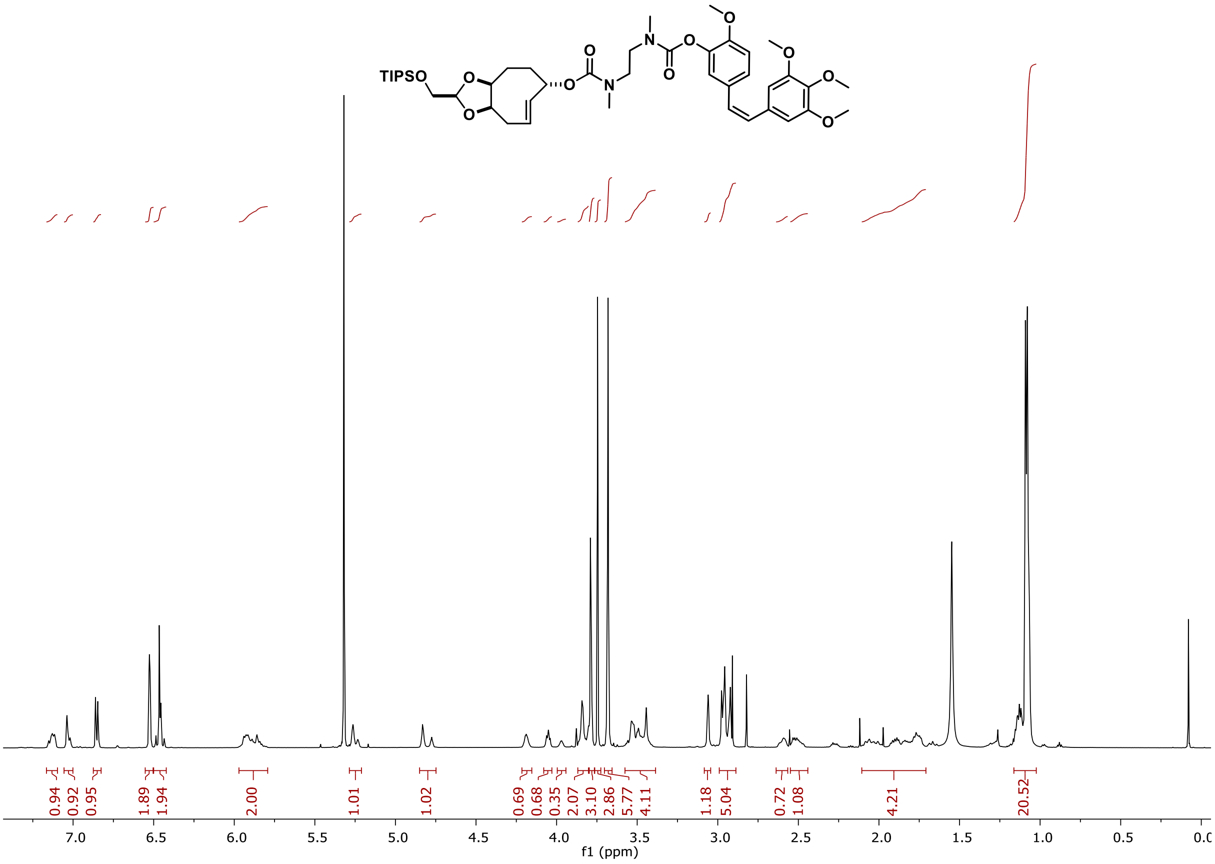

Compound **14,** ^{1}H-NMR

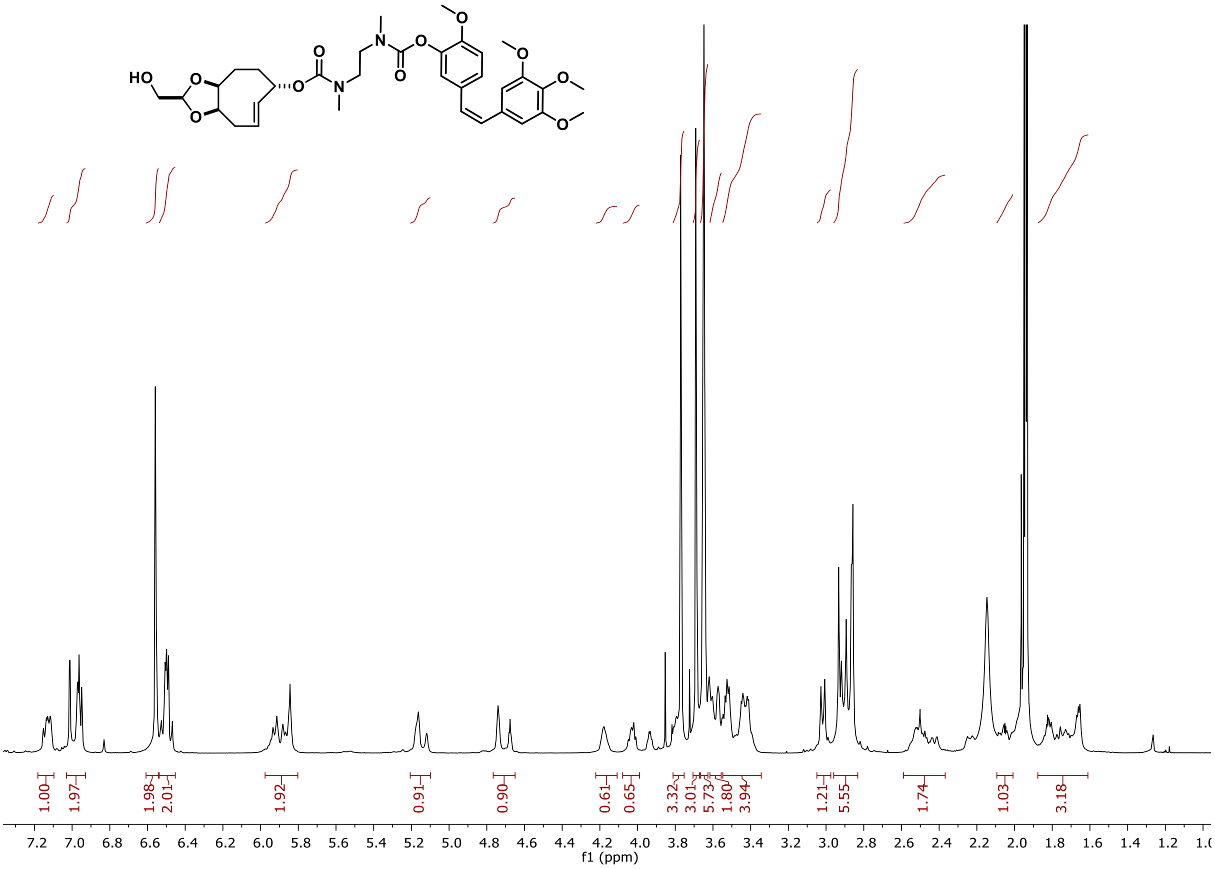

Compound **14,** ^{13}C-NMR

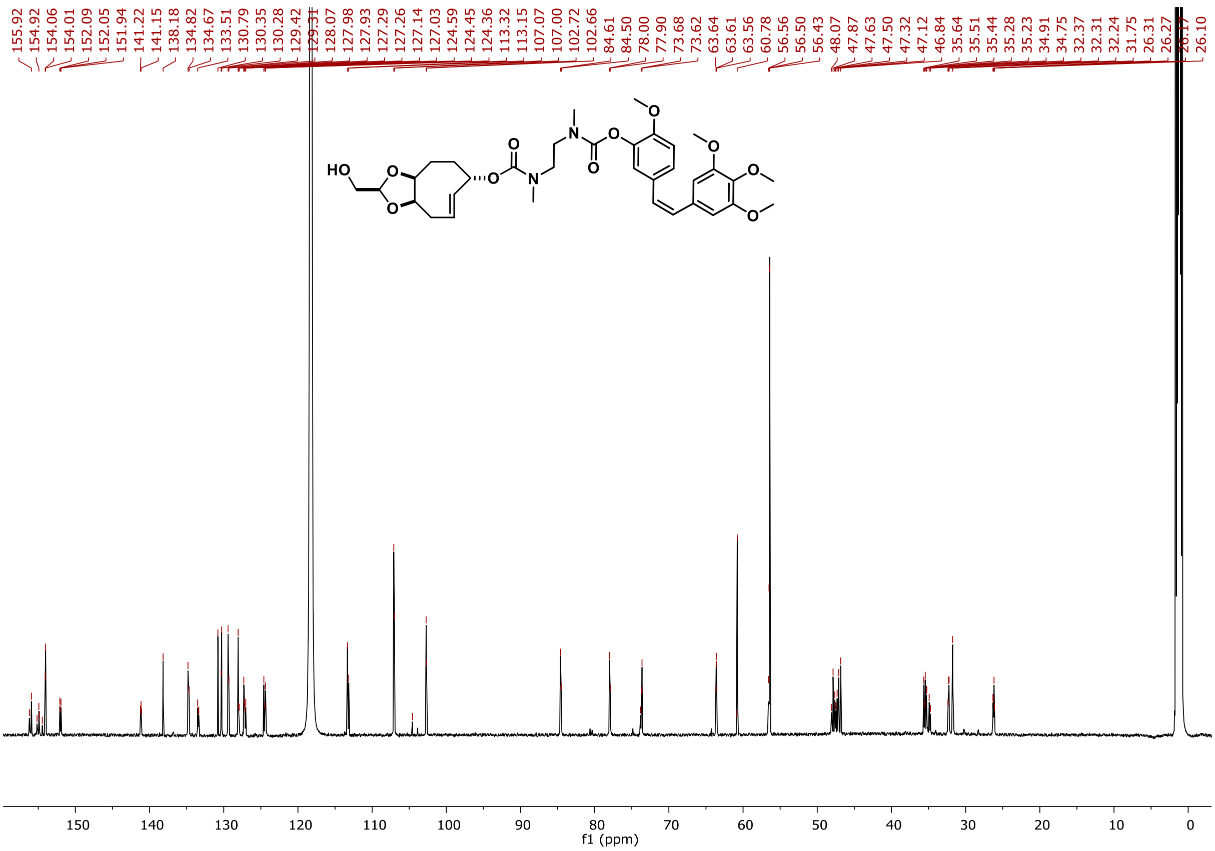

Compound **14**, HPLC (buffered conditions, ammonium formate (pH 8.4)/acetonitrile gradient elution)

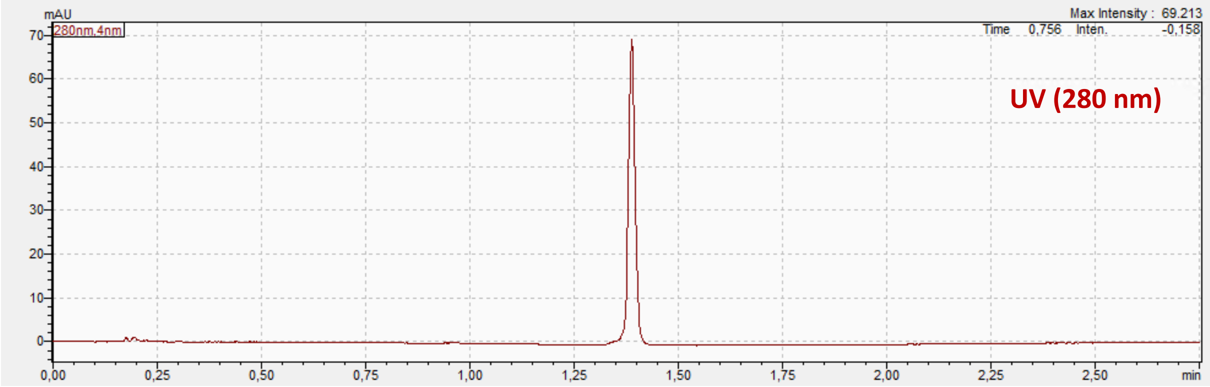

Compound **15,** ^{1}H-NMR

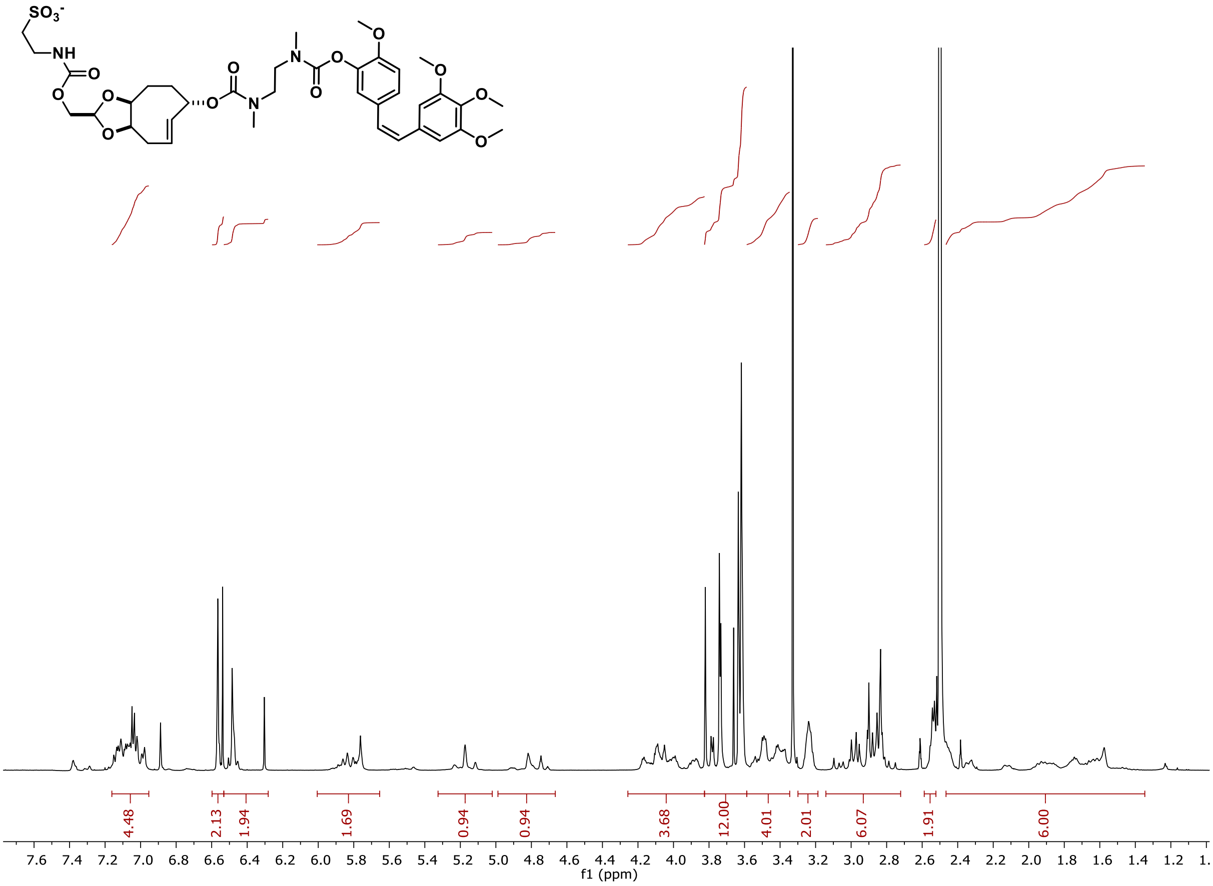

Compound **15,** HPLC (buffered conditions, ammonium formate (pH 8.4)/acetonitrile gradient elution)

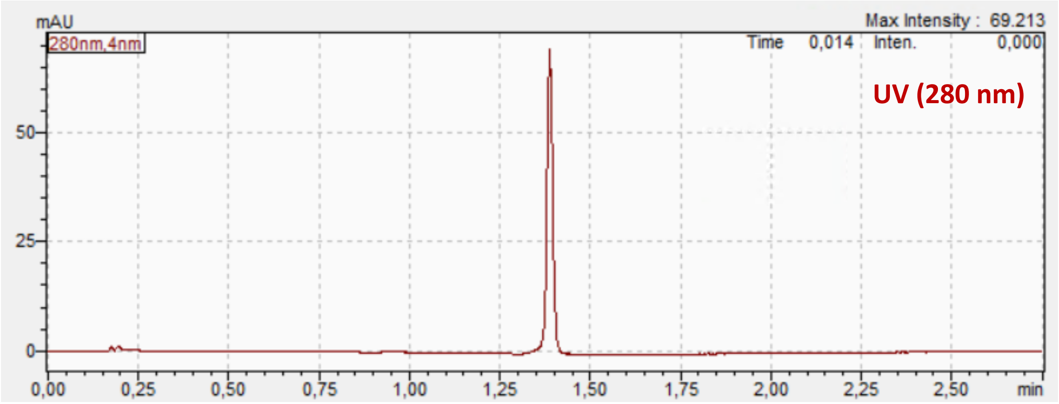

Compound **16,** ^{1}H-NMR

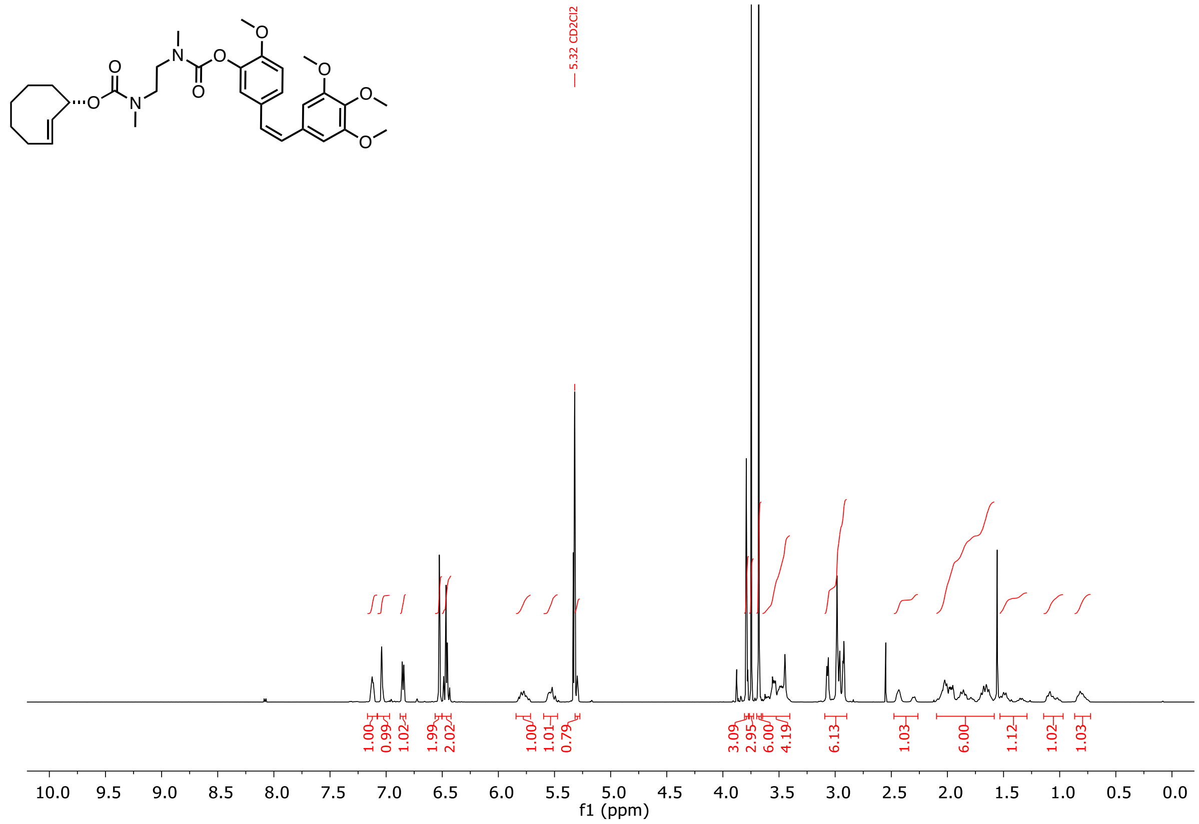

Compound **16,** ^{13}C-NMR

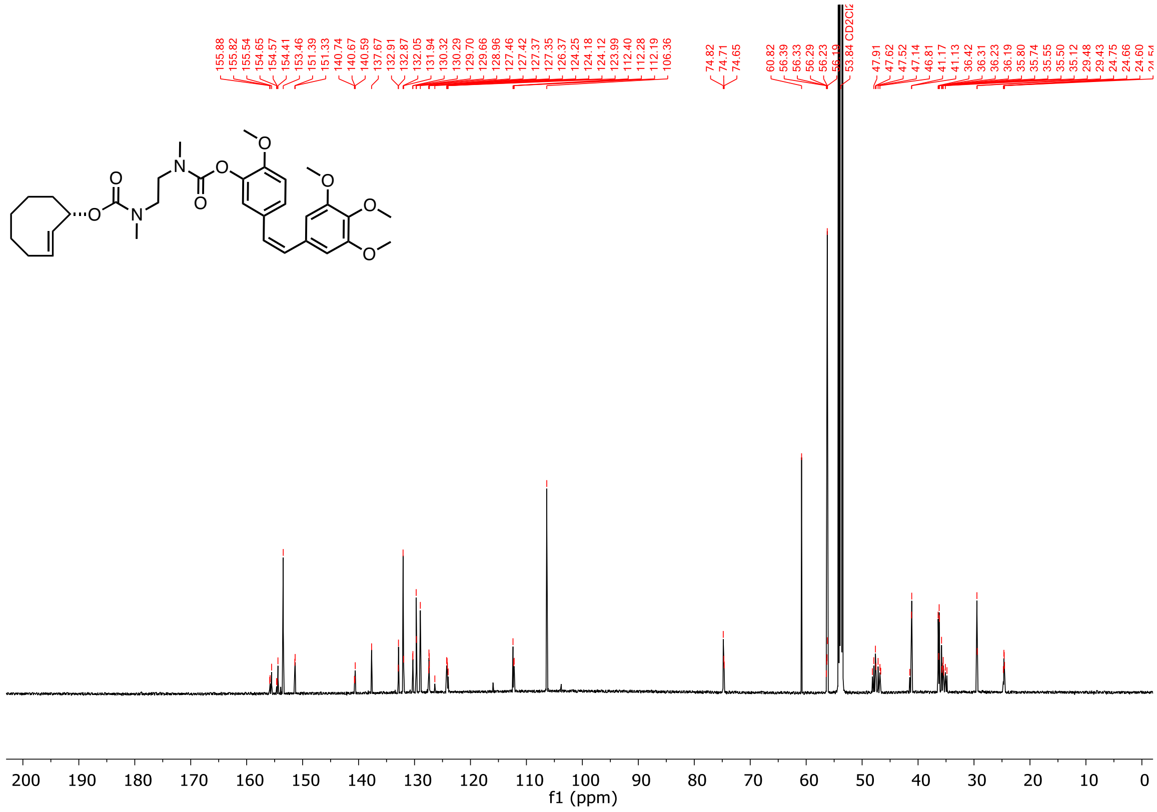

10) References

[1] A. Darko, S. Wallace, O. Dmitrenko, M. M. Machovina, R. A. Mehl, J. W. Chin, J. M. Fox, *Chem. Sci.* **2014**, *5*, 3770-3776.

[2] T. Hori, K. B. Sharpless, *J. Org. Chem.* **1978**, *43*, 1689-1697.

[3] D. Svatunek, C. Denk, V. Rosecker, B. Sohr, C. Hametner, G. Allmaier, J. Fröhlich, H. Mikula, *Monatsh. Chem.* **2016**, *147*, 579-585.

[4] A. Darko, S. J. Boyd, J. M. Fox, *Synthesis* **2018**, *50*, 4875-4882.

[5] P. Keppel, B. Sohr, W. Kuba, P. Skrinjar, J. C. T. Carlson, H. Mikula, *ChemRxiv* **2022**, DOI 10.26434/chemrxiv-2022-nt32f.

[6] J. C. T. Carlson, H. Mikula, R. Weissleder, *J. Am. Chem. Soc.* **2018**, *140*, 3603-3612.

[7] R. M. Versteegen, R. Rossin, W. ten Hoeve, H. M. Janssen, M. S. Robillard, *Angew. Chem. Int. Ed.* **2013**, *52*, 14112-14116.

[8] A. J. C. Sarris, T. Hansen, M. A. R. de Geus, E. Maurits, W. Doelman, H. S. Overkleeft, J. D. C. Codee, D. V. Filippov, S. I. van Kasteren, *Chem. Eur. J.* **2018**, *24*, 18075-18081.

[9] G. Lukinavičius, L. Reymond, E. D'Este, A. Masharina, F. Göttfert, H. Ta, A. Güther, M. Fournier, S. Rizzo, H. Waldmann, C. Blaukopf, C. Sommer, D. W. Gerlich, H.-D. Arndt, S. W. Hell, K. Johnsson, *Nature Methods* **2014**, *11*, 731-733.

[10] G. Sheldrick, *Acta Crystallogr. A* **2015**, *71*, 3-8.

[11] G. Sheldrick, *Acta Crystallogr. C* **2015**, *71*, 3-8.

[12] P. R. Edgington, P. McCabe, C. F. Macrae, E. Pidcock, G. P. Shields, R. Taylor, M. Towler, J. Van De Streek, *J. Appl. Crystallogr.* **2006**, *39*, 453-457.

[13] J.-D. Chai, M. Head-Gordon, *Phys. Chem. Chem. Phys.* **2008**, *10*, 6615-6620.

[14] F. Weigend, R. Ahlrichs, *Phys. Chem. Chem. Phys.* **2005**, *7*, 3297-3305.

[15] P. Pracht, F. Bohle, S. Grimme, *Phys. Chem. Chem. Phys.* **2020**, *22*, 7169-7192.

[16] Luchini, G.; Alegre-Requena; IFunes; Rodríguez-Guerra, J.; Chen, J.; Paton, R. Bobbypaton/ goodvibes: Goodvibes V3.0.0. Zenodo July 23, **2019**. https://doi.org/10.5281/zenodo.3346166.
